# Modeling 3D Facial Shape from DNA

**DOI:** 10.1371/journal.pgen.1004224

**Published:** 2014-03-20

**Authors:** Peter Claes, Denise K. Liberton, Katleen Daniels, Kerri Matthes Rosana, Ellen E. Quillen, Laurel N. Pearson, Brian McEvoy, Marc Bauchet, Arslan A. Zaidi, Wei Yao, Hua Tang, Gregory S. Barsh, Devin M. Absher, David A. Puts, Jorge Rocha, Sandra Beleza, Rinaldo W. Pereira, Gareth Baynam, Paul Suetens, Dirk Vandermeulen, Jennifer K. Wagner, James S. Boster, Mark D. Shriver

**Affiliations:** 1 Medical Image Computing, ESAT/PSI, Department of Electrical Engineering, KU Leuven, Medical Imaging Research Center, KU Leuven & UZ Leuven, iMinds-KU Leuven Future Health Department, Leuven, Belgium; 2 Department of Anthropology, Penn State University, University Park, Pennsylvania, United States of America; 3 Smurfit Institute of Genetics, Dublin, Ireland; 4 Department of Genetics, Stanford University, Palo Alto, California, United States of America; 5 HudsonAlpha Institute for Biotechnology, Huntsville, Alabama, United States of America; 6 CIBIO: Centro de Investigação em Biodiversidade e Recursos Genéticos, Universidade do Porto, Porto, Portugal; 7 Departamento de Biologia, Faculdade de Ciências, Universidade do Porto, Porto, Portugal; 8 IPATIMUP: Instituto de Patologia e Imunologia Molecular da Universidade do Porto, Porto, Portugal; 9 Programa de Pós-Graduação em Ciências Genômicas e Biotecnologia, Universidade Católica de Brasília, Brasilia, Brasil; 10 School of Paediatrics and Child Health, University of Western Australia, Perth, Australia; 11 Institute for Immunology and Infectious Diseases, Murdoch University, Perth, Australia; 12 Genetic Services of Western Australia, King Edward Memorial Hospital, Perth, Australia; 13 Center for the Integration of Genetic Healthcare Technologies, University of Pennsylvania, Philadelphia, Pennsylvania, United States of America; 14 Department of Anthropology, University of Connecticut, Storrs, Connecticut, United States of America; Seattle Children's Research Institute, United States of America

## Abstract

Human facial diversity is substantial, complex, and largely scientifically unexplained. We used spatially dense quasi-landmarks to measure face shape in population samples with mixed West African and European ancestry from three locations (United States, Brazil, and Cape Verde). Using bootstrapped response-based imputation modeling (BRIM), we uncover the relationships between facial variation and the effects of sex, genomic ancestry, and a subset of craniofacial candidate genes. The facial effects of these variables are summarized as response-based imputed predictor (RIP) variables, which are validated using self-reported sex, genomic ancestry, and observer-based facial ratings (femininity and proportional ancestry) and judgments (sex and population group). By jointly modeling sex, genomic ancestry, and genotype, the independent effects of particular alleles on facial features can be uncovered. Results on a set of 20 genes showing significant effects on facial features provide support for this approach as a novel means to identify genes affecting normal-range facial features and for approximating the appearance of a face from genetic markers.

## Author Summary

The face is perhaps the most inherently fascinating and aesthetic feature of the human body. It is a principle subject of art throughout human history and across cultures and populations. It provides the most significant means by which we communicate our emotions and intentions in addition to health, sex, and age. And yet features such as the strength of the brow ridge, the spacing between the eyes, the width of the nose, and the shape of the philtrum are largely scientifically unexplained. Here, we use a novel method to measure face shape in population samples with mixed West African and European ancestry from three locations (United States, Brazil, and Cape Verde). We show that facial variation with regard to sex, ancestry, and genes can be systematically studied with our methods, allowing us to lay the foundation for predictive modeling of faces. Such predictive modeling could be forensically useful; for example, DNA left at crime scenes could be tested and faces predicted in order to help to narrow the pool of potential suspects. Further, our methods could be used to predict the facial features of descendants, deceased ancestors, and even extinct human species. In addition, these methods could prove to be useful diagnostic tools.

## Introduction

The craniofacial complex is initially modulated by precisely-timed embryonic gene expression and molecular interactions mediated through complex pathways [1]. As humans grow, hormones and biomechanical factors also affect many parts of the face [2], [3]. The inability to systematically summarize facial variation has impeded the discovery of the determinants and correlates of face shape. In contrast to genomic technologies, systematic and comprehensive phenotyping has lagged. This is especially so in the context of multipartite traits such as the human face. In typical genome-wide association studies (GWAS) today phenotypes are summarized as univariate variables, which is inherently limiting for multivariate traits, which, by definition cannot be expressed with single variables. Current state-of-the-art genetic association studies for facial traits are limited in their description of facial morphology [4]–[7]. These analyses start from a sparse set of anatomical landmarks (these being defined as “a point of correspondence on an object that matches between and within populations”), which overlooks salient features of facial shape. Subsequently, either a set of conventional morphometric measurements such as distances and angles are extracted, which drastically oversimplify facial shape, or a set of principal components (PCs) are extracted using principal components analysis (PCA) on the shape-space obtained with superimposition techniques, where each PC is assumed to represent a distinct morphological trait. Here we describe a novel method that facilitates the compounding of all PCs into a single scalar variable customized to relevant independent variables including, sex, genomic ancestry, and genes. Our approach combines placing spatially dense quasi-landmarks on 3D images [8], [9], principal component analysis (PCA), and a new partial least squares regression (PLSR, [10]) derived method we call “bootstrapped response-based imputation modeling” (BRIM) to measure and model facial shape variation (Text S1, Figures S1, S2, S3).

Given the multivariate nature of the face and the large number of genes likely affecting variation in the face, we chose to focus attention on the between-population variation with a genetic admixture approach using research participants from three West African/European admixed populations. Ancestry informative markers (AIMs) can be used to estimate individual genomic ancestry from DNA [11], which can be used to investigate population differences and map genes for genetically determined traits that vary between populations. Non-random mating and continuous gene flow in admixed populations results in admixture stratification or variation in individual ancestry [12], [13]. The process of admixture also results in admixture linkage disequilibrium or the non-random association among both AIMs and traits that vary between the parental populations. These characteristics make admixed populations uniquely suited to investigations into the genetics of such traits [14]–[16]. By simultaneously modeling facial shape variation as a function of sex and genomic ancestry along with genetic markers in craniofacial candidate genes, the effects of sex and ancestry can be removed from the model thereby providing the ability to extract the effects of individual genes.

## Results/Discussion

**Figure 1:**
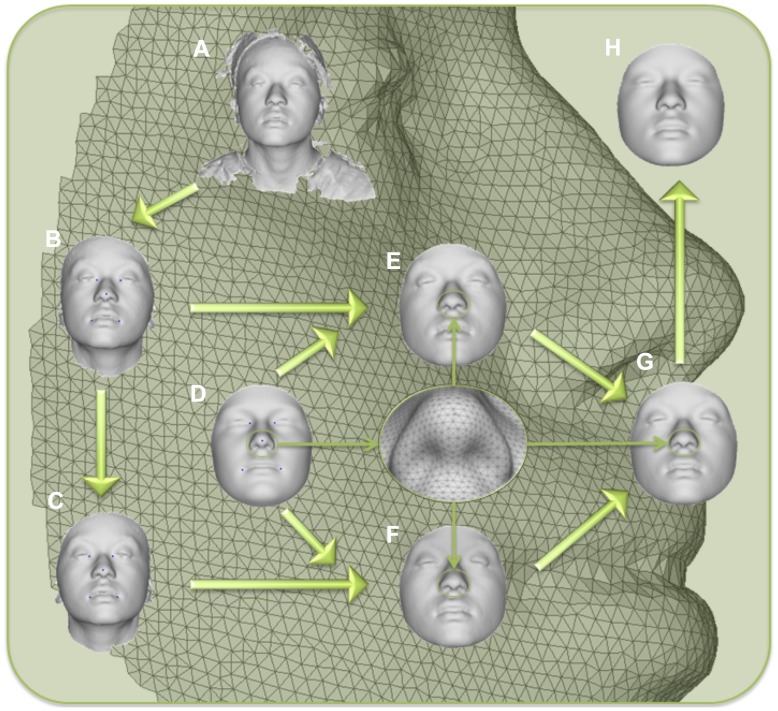
Workflow for 3D face scan processing. A) original surface, B) trimmed to exclude non-face parts, C) reflected to make mirror image, D) anthropometric mask of quasi-landmarks, E) remapped, F) reflected remapped, G) symmetrized, H) reconstructed.

**Figure 2:**
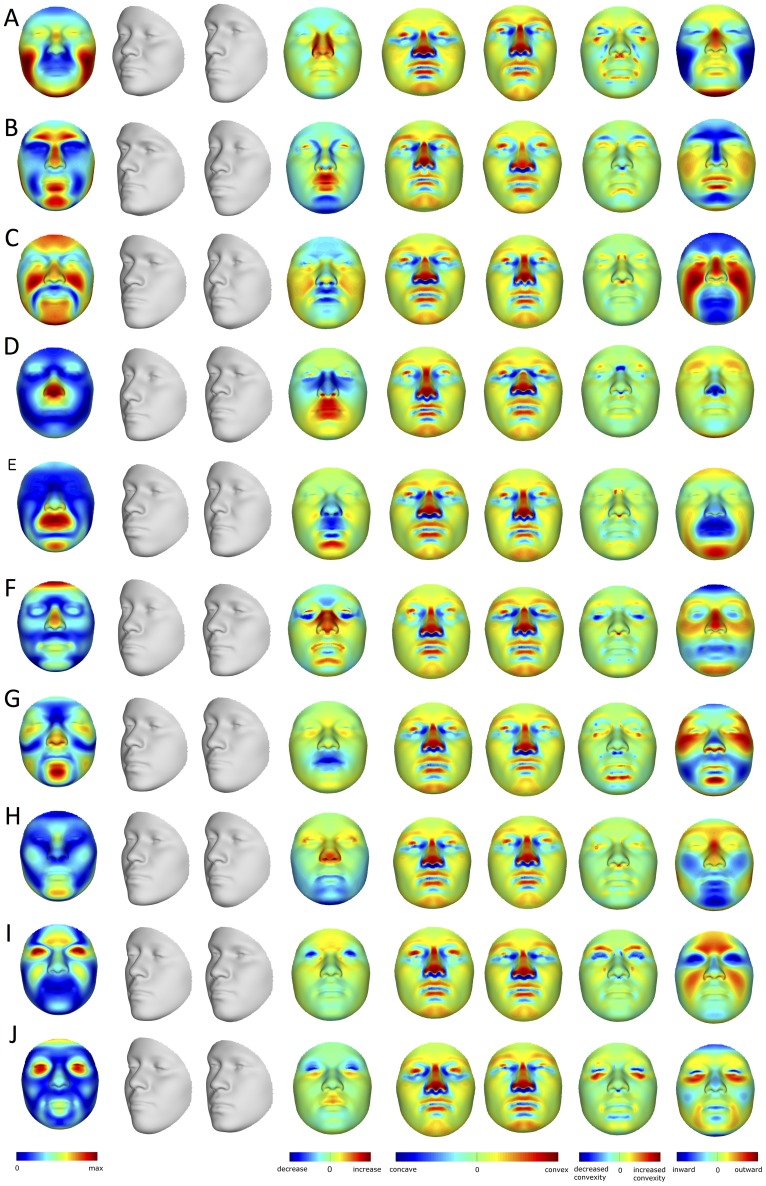
PCA effects on facial morphology. The effects of the first 10 PCs (A–J) on face shape change parameters (FSCPs). The effect as a magnitude of each quasi-landmark displacement is shown first, followed by the alternate transformations (grey faces), the area ratio between both, the curvatures on the transformations, the curvature ratio between both, and finally the normal displacement between both, which is the signed magnitude of the displacement of one quasi-landmark in the direction normal to the surface of the first transformation (left gray faces).

A spatially dense mesh of 7,150 quasi-landmarks was used to map 3D images of participants' faces onto a common coordinate system (Figure 1). Quasi-landmarks are defined here as largely homologous vertices in this mapped mesh. The mesh is applied automatically, eliminating the difficult and error-prone procedure of manually indicating facial landmarks [8], [9], [17]. Deviations from bilateral symmetry were removed by averaging each face with its mirror image [18], [19]. PCA on the symmetrized 21,450 quasi-landmark 3D coordinates (X, Y, and Z for each of the 7,150 quasi-landmarks) using all 592 participants produces 44 principal components (PCs) that together summarize 98% of the variation in face shape and define a multidimensional face space. The effects of the first 10 PCs are illustrated in Figure 2. Some of these PCs (*e.g.*, PC4, PC5) capture the effects of changes in only particular parts of the face. However, many PCs (*e.g.*, PC1, PC2, PC3) capture effects in multiple parts of the face. Moreover, although the PCs are statistically independent, any particular part of the face is affected by several PCs. As such, it is likely incorrect to assume that each PC represents a distinct morphological trait resulting from the action of specific genes. Our use of BRIM to combine the independent effects of PCs is agnostic about their biological meaning, if any, and provides for the compounding of the information from any or all of the PCs together into a single variable that is customized to the predictor variable being modeled. In this way, BRIM also overcomes the problem of multiple testing inherent to other methods for summarizing facial variation. In other words, the hypothesis, *does this gene have significant effects on facial shape*, can be addressed with a single statistical test (Text S1).

BRIM is an extension of existing relationship modeling techniques that uses response variables to refine and, in some cases, to transform one or more initial predictor variables. In other words and in contrast to alternate techniques, BRIM uses a multivariate matrix of response variables in a leave-one-out forced imputation setup to update the initial predictor variable values, creating a new type of variable – the response-based imputed predictor (RIP) variable (Figure S2). The BRIM process is bootstrapped, and estimator improvement over successive iterations can be monitored (Figures S5, S6, S7, S8, S9). BRIM also functions to correct observation error, misspecification of predictor values, and other sources of statistical confounding (Text S1). Within the iterative bootstrapping scheme, a nested leave-one-out approach is used to avoid model over-fitting and to allow hypothesis testing using standard statistical techniques, such as correlation analysis, ANOVA, and receiver operating characteristic (ROC) curve analysis [20], to test the significance of the association between the predictors and RIP variables. Likewise, the relationships between the RIP variables and the response variables, *e.g.*, the 21,450 facial parameters, allows for the visualization and quantitation of their effects on face shape.

RIP variables modeling sex (RIP-S) and genomic ancestry (RIP-A), as well as those modeling the effects of particular genetic markers (RIP-Gs), can be visualized using two primary methods – shape transformations and heat maps. We used three summary statistics (area ratio, normal displacement, and curvature difference), which can be illustrated using heat maps, to quantify the particular changes to the face that result. These measures of facial change, along with particular inter-landmark distances, angles, and spatial relationships, can together be termed *face shape change parameters* (FSCPs). FSCPs provide a means of translating face shape changes from the abstract face space into both visual representations into words. Such terms are used in clinical and anthropological descriptions of faces and by doing so we can compare these to the BRIM results (*e.g.*, Figures S28, S29, S30, S31, S32, S33, S36, S37, S38, and Table S1). The statistical significance of these and related FSCPs can be tested using permutation.

**Figure 3:**
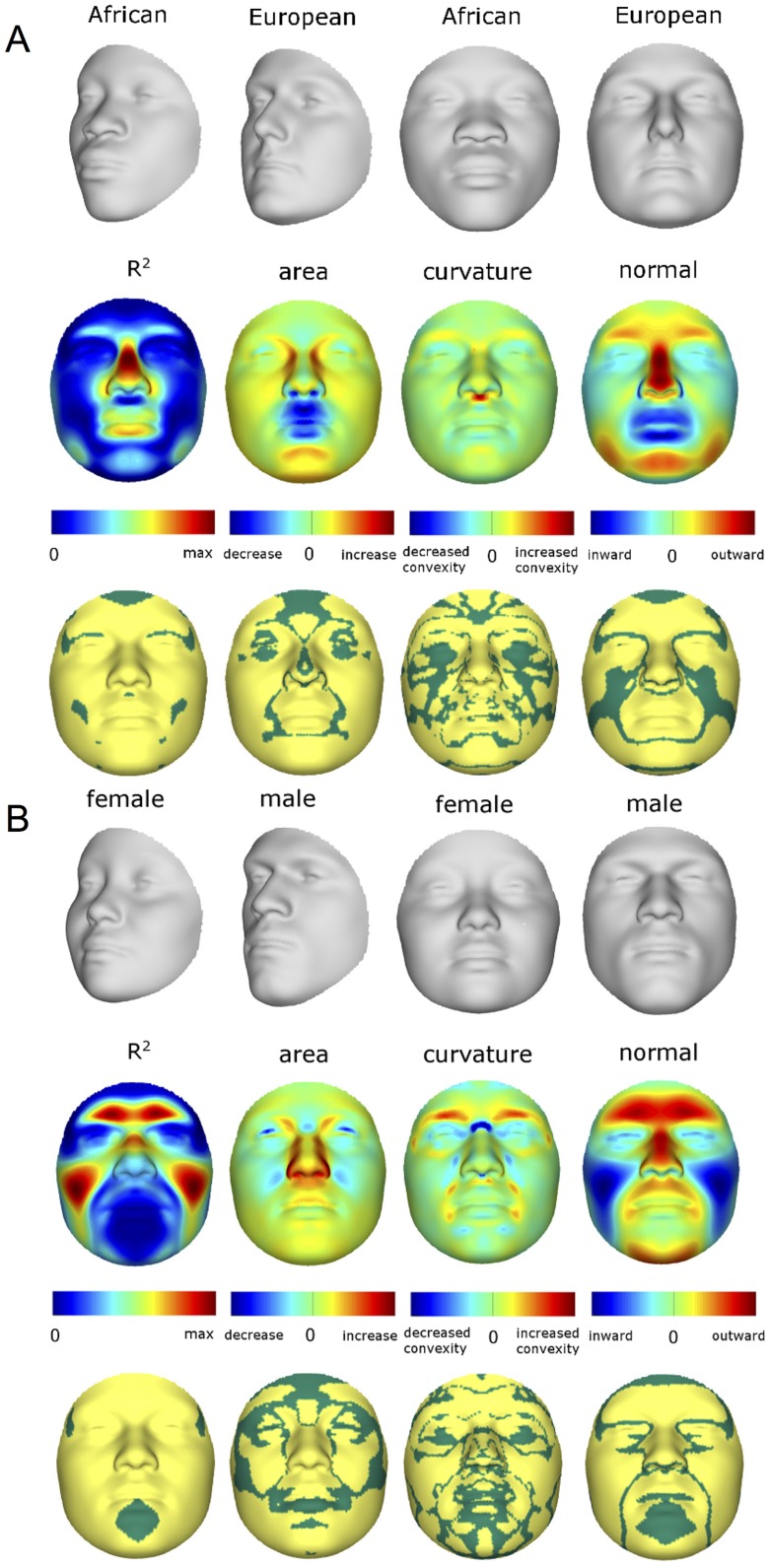
Transformations and heat maps showing how face shape is affected by (A) RIP-A and (B) RIP-S. The top row of each panel shows the shape transformations three standard deviations below and above the mean of the RIPs in this sample. The second row shows the R^2^ (proportion of the total variation in each quasi-landmark) and the three primary facial shape change parameters: area ratio, curvature difference, and normal displacement. The bottom row shows in yellow the regions of the face that are statistically significantly different (*p*<0.001) between the two transformations. The max R^2^ values for RIP-A and RIP-S are 40.83% and 38.21%, respectively.

As expected, many parts of the face are affected by both ancestry and sex. Figure 3 illustrates the partial effects of RIP-A and RIP-S on facial shape using transformations and heat maps for effect size (R^2^) and the three primary FSCPs. Facial regions that are statistically significant (*p*<0.001) for effect size and the FSCPs are shown in Figure 3 as the yellow (not green regions in the bottom panels). The RIP-A and RIP-S shape transformations shown are set to the points three standard deviations plus and minus the mean RIP-A and RIP-S levels in these samples. As seen in the effect-size (R^2^) panels in Figure 3, the proportion of the total variance in particular facial features explained by RIP-A and RIP-S can be substantial. In general, up to a third of the variance in several parts of the face is explained by these two variables. RIP-A primarily affects the nose and lips and, to lesser extents, the roundness of the face, the mandible, and supraorbital ridges. Sex has a much larger effect than ancestry on the supraorbital ridges and cheeks, and smaller effects on the nose and under the eyes. The FSCPs help to illustrate the specific ways in which particular RIP variables affect the face. For example, the area ratio shows increased surface area for the medial canthus, sides of the nose, and front of the chin on the European end of RIP-A and a greater surface area for the nostrils and lips on the West African end of RIP-A. The curvature difference highlights the top of the philtrum as a facial feature that is highly convex on the European end and highly concave on the West African end of RIP-A. Regions showing curvature differences for RIP-A are also seen in the nasal bridge, supraorbital ridges, and chin. RIP-S shows greatest effects on the supraorbital ridges, nasal bridge, nasal ridge, zygomatics, and cheeks. The nose, lips, medial canthus, and mandible are also affected by RIP-S. The largest differences in facial curvature related to changes in RIP-S are on the supraorbital ridges and the nasal bridge.

**Figure 4:**
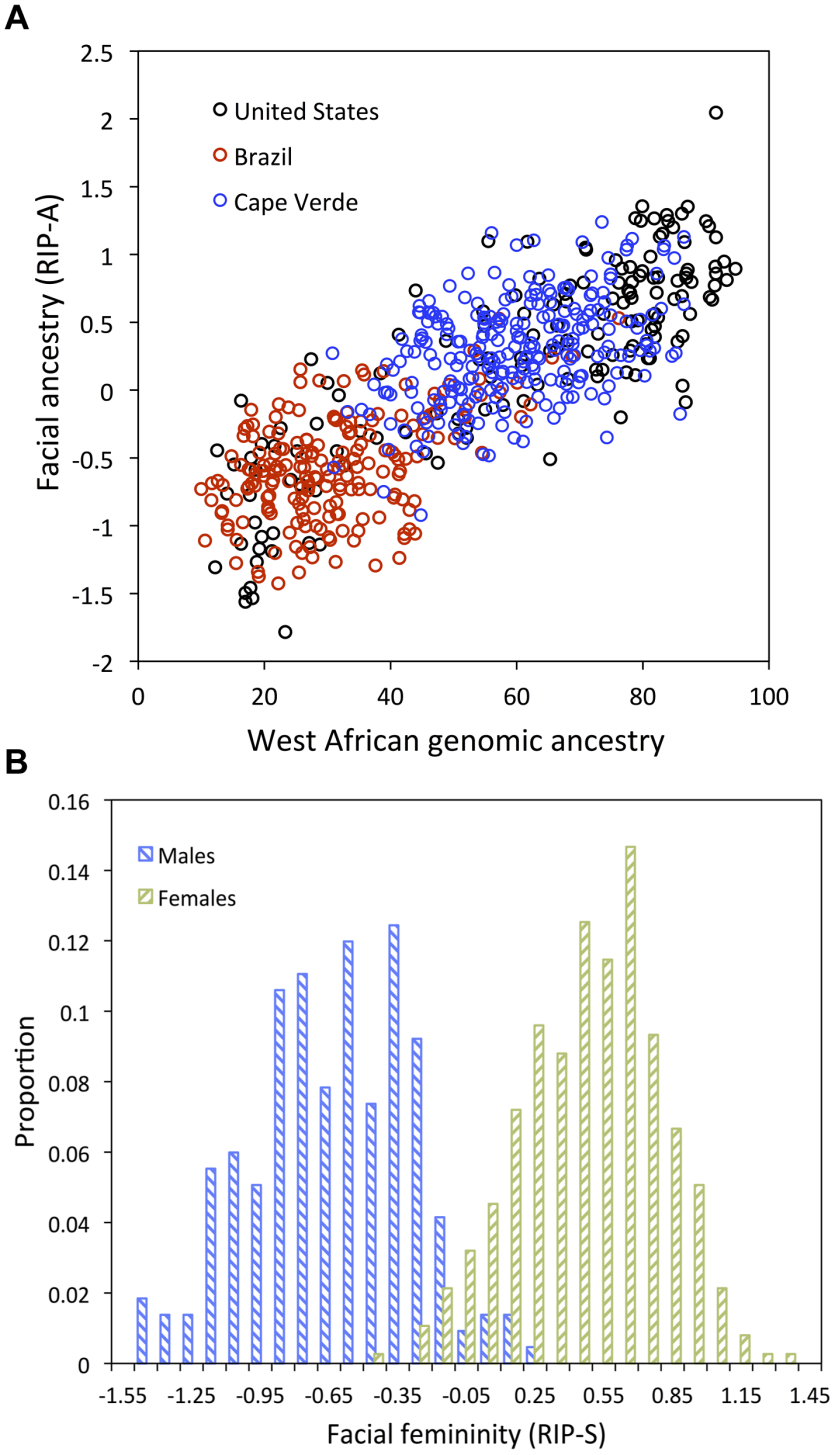
Relationships between the ancestry and sex RIP variables and their initial predictor variables. (A) RIP-A with genomic ancestry; genomic ancestry is calculated using the core panel of 68 AIMs and RIP-A is calculated using this ancestry estimate on the set of three populations combined (N = 592). Populations are indicated as shown in the legend with United States participants shown with black circles, Brazilians with red circles, and Cape Verdeans with blue circles. (B) Histograms of RIP-S by self-reported sex.

Despite the complex ways in which faces are affected by RIP-A and RIP-S, these variables are useful summaries of the degree to which particular faces are more or less ancestry-typical and sex-typical, respectively. This is evident in the strong relationship observed between RIP-A and genomic ancestry as measured with a panel of 68 AIMs (r = 0.81, *p*<0.001; Figure 4A). Approximately two thirds of the variation in RIP-A across these three West African/European admixed populations is explained by genomic ancestry. Likewise, as seen in Figure 4B, RIP-S is very distinctive between the sexes. ROC analyses (Figure S32) show that the AUC for RIP-S on sex is 0.994 (*p*<0.001). Genomic ancestry, independently from sex, explains 9.6% of the total facial variation, while sex independently from ancestry explains 12.9% of the total facial variation (Table S3). Most facial variation, like human genetic variation in general, is shared among different human populations and by members of both sexes.

We used alternate subsets of AIMs and alternate population samples to test the robustness of the facial ancestry (RIP-A) estimation. RIP-A values were derived using different initial predictor variables and compared. The pairwise correlations of RIP-A estimates are high (R^2^>0.99), showing that very similar estimates of facial ancestry result from different panels of AIMs (Figure S9) and alternate population samples (Figures S10, S11). The robustness of RIP-A estimates to both marker panel and population sample substantiates the generality and, thus, practical usefulness of these models.

We also see that RIP-A estimates generated using AIMs panels with lower ancestry-information content show stronger correlations with more accurate genomic ancestry estimates than with the genomic ancestry estimates that were used to generate them (Figure S9). To further evaluate the performance of BRIM when less information is available, we performed noise injection experiments by adding or subtracting randomly defined quantities from the estimates of genomic ancestry and misclassifying the sex of persons in the sample (Figures S4, S5, S6, S7, S8 and Figures S12, S13, S14, respectively). These experiments demonstrate the same patterns noted above using alternate panels of AIMs: Accurate RIP variables for these two traits are possible with incorrect coding of sex and imprecise estimates of genomic ancestry. The initial predictor variable values of both sex and ancestry can be reduced in precision by as much as 30% (*i.e.*, r^2^ = 0.7 between the original predictor variable and the noise predictor injected variable) and still show correlation coefficients of about r = 0.95 between the RIP measures generated with these noisy estimates and RIP measures generated with the original estimates (Figure S8 and Figure S14). BRIM is efficient in using the latent covariance structure of the facial PCs to discover the paths through face space that reflect sex and ancestry and can accurately summarize the relative positions of individual faces on these paths as RIP-S and RIP-A, respectively.

**Figure 5:**
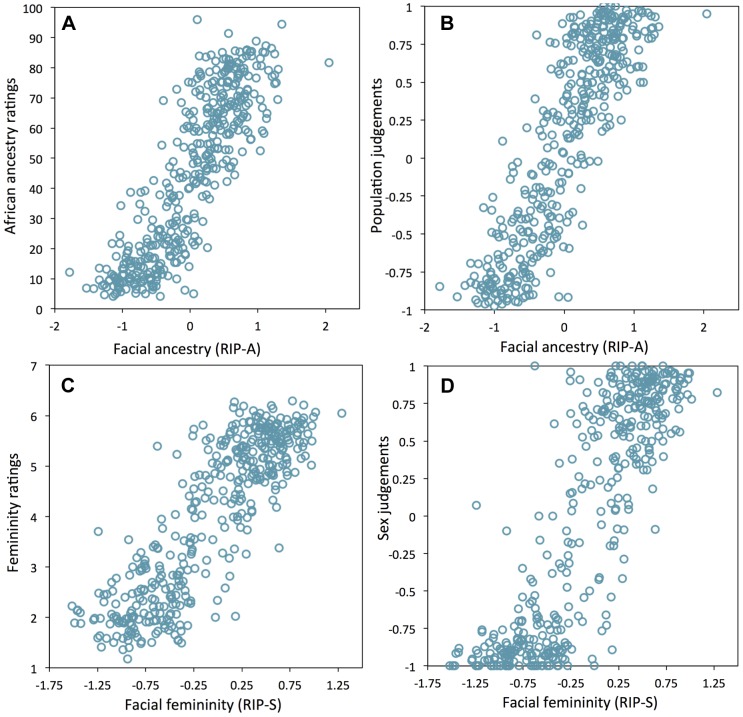
Relationships between human observer rating and judgments of facial ancestry and sex. (A) RIP-A and proportional ancestry ratings (r = 0.854, *p*<0.0001), (B) RIP-A and ancestry judgments (r = 0.859, *p*<0.0001), (C) RIP-S and femininity ratings (r = 0.860, *p*<0.0001), (D) RIP-S and sex judgments (r = 0.856, *p*<0.0001).

Humans are also very adept at observing faces and can infer many aspects of the variability among faces [21], [22]. Given this, we attempted to test whether the human observer might provide a means of validating the RIP-A and RIP-S variables. Observers were shown false-colored 3D animated GIF images of research participants' faces and asked to rate the proportion of West African ancestry (from 0% to 100%) and the femininity (using a Likert scale from 1 to 7). Observers were also asked to judge the sex and the population group. As shown in Figures 5A and 5B, the correlations between RIP-A and observer ratings of proportional facial ancestry and judgments of facial population are strong (all r>0.85 and *p*<0.0001). Similarly, RIP-S and observer ratings of facial femininity and judgments of facial sex are also highly correlated (r>0.85 and *p*<0.0001; Figures 5C and 5D). These findings provide additional validation that RIP-A and RIP-S are informative summary statistics representing the relative levels of facial ancestry and facial femininity.

Like sex and genomic ancestry, SNP genotypes can be used as initial predictor variables in BRIM resulting in one RIP-G variable per SNP. We performed a partial BRIM analysis modeling genotype effects independent of sex and ancestry for each of 76 West African/European ancestry-informative SNPs located in 46 craniofacial candidate genes. These 46 genes were selected primarily from a set of 50 craniofacial genes that also showed genomic signatures of accelerated evolution in a survey of 199 genes (Table S2). Since properly conditioned tests of genetic association in admixed populations are an efficient approach to discover genes affecting traits that differ between populations and since RIP-A is an efficient means of summarizing overall facial ancestry, it is perhaps somewhat counterintuitive that RIP-A conditioning is superior to genomic ancestry conditioning in our partial BRIM modeling (Figures S15, S16, S17, S18, S19, S20 and S27). Likewise, RIP-S proved to be a better conditioning variable than sex in the partial BRIM analyses to estimate RIP-G (Figures S21, S22, S23, S24, S25, S26). We performed ANOVAs to test for average differences in RIP-G by genotype category (*e.g.*, CC, CT, and TT coded as −1, 0, and 1 assuming additive allelic effects). Given the substantial *a priori* evidence, *viz.*, that these genes show evidence of accelerated evolution in one or both of the parental populations and that mutations in these genes can cause overt murine or human craniofacial dysmorphology, we consider our analysis of each gene to be a separate statistical test and, as such, do not require adjustments for multiple testing. Twenty-four of 76 RIP-G variables (in 20 different genes) show *p*<0.1 (Table S2). The relatively low threshold was motivated by the strong *a priori* evidence for each gene noted above, the single trait summary provided by RIP-G, and an expected small effect of single genes on normal-range variation across the whole face. Additionally, given the general finding that clinically relevant genes can also affect subclinical and normal-range variation (e.g., [23]), we performed detailed *post hoc* descriptions of the effects of these RIP-Gs using FSCPs (Figures S34, S35, Figures S39, S40, S41, S42, S43, S44 and Table S4).

**Figure 6:**
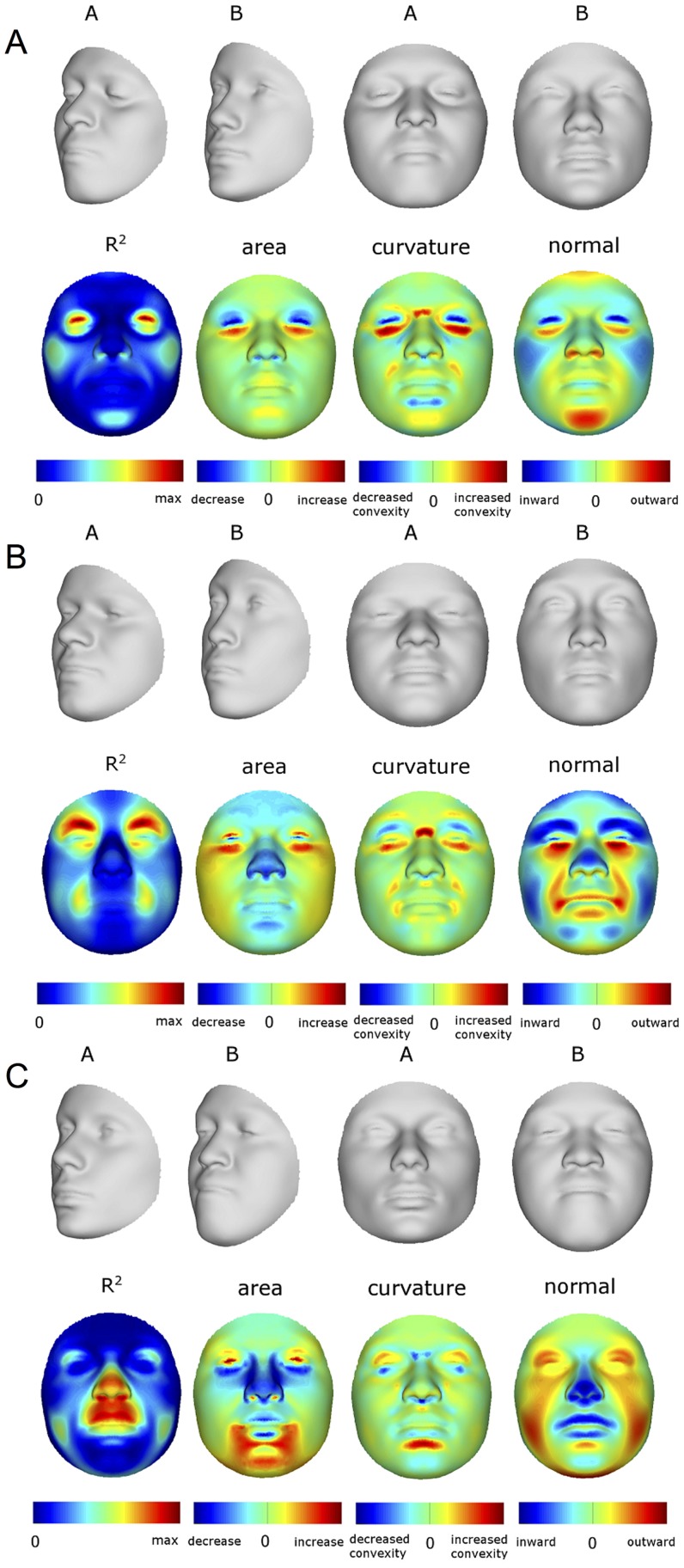
Transformations and heat maps showing how face shape is affected by three particular RIP-G variables. The initial predictor variables are SNPs in the genes (A) *SLC35D1* (B) *FGFR1*, and (C) *LRP6*. The top row of each panel shows the shape transformations near the extreme values of the particular RIP-G shown. The second row shows the R^2^ (proportion of the facial total variation), the three primary facial shape change parameters: area ratio, curvature difference, and normal displacement. The max R^2^ values for A, B, and C are 11.68%, 15.16% and 10.10%, respectively.

Summaries of the effects of three of these 24 RIP-G variables (rs1074265 in *SLC35D1*, rs13267109 in *FGFR1* and rs2724626 in *LRP6*) presented in Figures 6A, 6B, and 6C illustrate these results. A detailed analysis and description of each of the 24 SNP effects using FSCPs is given in the supporting material (Text S1). The gene solute carrier family 35 member D1 gene (*SLC35D1*; OMIM#610804) is located on human chromosome 1p31.3 [24]. Mutations in *SLC35D1* have been shown to result in Schneckenbecken dysplasia (OMIM#269250), which affects the face causing the characteristic feature of “superiorly oriented orbits.” The normal-range results of the SNP in rs1074265 in *SLC35D1* (Figure 6A) indicate strong effects at the eyes and periorbital regions, including notable differences at the supraorbital region, as well as at the midface and the chin. Mutations in the human fibroblast growth factor receptor 1 (*FGFR1*;OMIM#136350) gene located on chromosome 8p21.23-p21.22 can result in four autosomal dominant craniofacial disorders: Jackson-Weiss syndrome (OMIM#123150), which is characterized by craniosynostosis and midfacial hypoplasia; trigonocephaly (OMIM#190440), which is characterized by a keel-shaped forehead resulting in a triangle-shaped cranium when viewed from above; osteoglophonic dysplasia (OMIM#166250), which is characterized by craniosynostosis prominent supraorbital ridge and depressed nasal root; and Pfeiffer syndrome (OMIM#101600), which is characterized by midface hypoplasia and, depending on the subtype, ocular proptosis, short cranial base, and cloverleaf skull. The normal-range results of the SNP rs13267109 in *FGFR1* depicted in Figure 6B indicate the strongest effects in the supraorbital ridges, the eyes, the midface, the nose, and the corners of the mouth. The strongest differences in the shape transformations are indeed the forehead, supraorbital ridges and nasal bridge. The mouse homologue of the human low-density lipoprotein receptor-related protein 6 (*LRP6*; OMIM#603507) gene is known to be critical for the development of lips in the mouse resulting in bilateral cleft lips in the knockout *LRP6* mouse model [25]. As yet, no human craniofacial diseases have been linked to the *LRP6* gene or to the gene region on human chromosome 12p13.2 although the gene product is known to interact on a molecular level with WNT signaling. Observing the shape transformation in Figure 6C, a change from a prominent lip region, including the appearance of a thick and convex vermilion, to a less prominent lip region, including an apparently thinner and less convex (more concave) vermilion, is noted. This is confirmed by inspecting the normal displacement results and the significance maps, in which the lips are clearly delineated (Figure S43).

In general, some RIP-G variables show localized effects (*e.g.*, rs1074265 in *SLC35D1*), changing only certain aspects in facial shape, while others display changes in several facial regions (*e.g.*, rs13267109 in *FGFR1*). Summary statistics for the underlying distributions of effect sizes across the quasi-landmarks are presented in Table S3. In the case where multiple SNPs in the same gene are modeled, overlapping and similar effects are seen across the different SNPs for the same gene (*e.g.*, *DNMT3B* and *SATB2*) and different SNPs from genes within the same biological pathway (*e.g.*, *WNT3*, *FGFR1*, and *FGFR2*). We present a graphical user interface (GUI) so that effects of changes in these 24 RIP-G variables, RIP-A, RIP-S, or any of the top 44 PC variables can be visualized in more detail. These transformations can be visualized with the texture map as well as shape only, and the GUI (http://tinyurl.com/DNA2FACEIN3D) allows for the illustration of the comparison of transformed faces to the consensus face using the three primary FSCPs.

Since both categorical and continuous variables can be modeled using BRIM, this approach might be used to test for relationships between facial features and other factors, *e.g.*, age, adiposity, and temperament. The methods illustrated here also provide for the development of diagnostic tools by modeling validated cases of overt craniofacial dysmorphology. Most directly, our methods provide the means of identifying the genes that affect facial shape and for modeling the effects of these genes to generate a predicted face. Although much more work is needed before we can know how many genes will be required to estimate the shape of a face in some useful way and many more populations need to be studied before we can know how generalizable the results are, these results provide both the impetus and analytical framework for these studies.

## Materials and Methods

### Population samples and participant recruitment

Population samples were collected in the United States (State College, PA, Williamsport, PA, and The Bronx, NY); Brasilia, Brazil; and Cape Verde (São Vicente, and Santiago), all under a Penn State University Internal Review Board (IRB) approved research protocol titled, “Genetics of Human Pigmentation, Ancestry and Facial Features.” Skin pigmentation was measured using narrow-band reflectometry with the DermaSpectrometer (Cortrex Technology, Hadsund, Denmark) in the United States and Brazil and the DSMII (Cortrex Technology, Hadsund, Denmark) in Cape Verde. DermaSpectrometer readings were rescaled to the DSMII scale by multiplying by 1.19, the slope derived from a comparison of readings with both instruments on the same set of participants (data not shown). Height, weight, age, self-reported ancestry, and sex were collected by survey. DNA was collected both with buccal cell brushes and using finger-stick blood on four-circle Whatman FTA cards (Whatman, Florham Park, NJ).

To minimize age-related variation in facial morphology, we only recruited participants between the ages of 18 and 40. From these recruits, we selected individuals with >10% West African ancestry and <15% combined Native American and East-Asian ancestry as measured with the 176 ancestry informative marker (AIM) panel. We assigned these cutoff points to reduce admixture from parental populations other than West African and European. Ancestry-based exclusion criteria were not applied to Cape Verdeans given the largely dihybrid nature of this population. Finally, we excluded participants whose 3D images were obstructed by facial or head hair. After excluding participants by these criteria, we were left with 592 participants (154 from the US, 191 from Brazil, and 247 from Cape Verde).

### SNP genotyping and genomic ancestry estimates

Genotyping of 176 AIMs for the US and Brazilian samples was performed on the 25 K SNPstream ultra-high-throughput genotyping system (Beckman Coulter, Fullerton, CA) as previously described [11]. Ancestry was estimated using the various panels of AIMs by one of two methods. Ancestry using full set of 176 AIMs was estimated in the US and Brazilian subsample using maximum likelihood on a four-population model; European, West African, Native American, and East Asian [11].The 68-AIM ancestry estimates were generated using the full sample (U.S., Brazilian, and Cape Verdean) using ADMIXMAP as these markers were available on all 592 participants. One marker (rs917502) from the original 176 had a call rate of less than 30% and was omitted from the ADMIXMAP analyses.

The Cape Verdean sample was assayed for the Illumina Infinium HD Human1M-Duo Beadarray (Illumina, San Diego, CA) following the manufacturer's recommendations. A total of 537,895 autosomal SNPs that passed quality controls were used to estimate ancestry using the program FRAPPE [26], assuming two ancestral populations (West African and European). HapMap genotype data, including 60 unrelated European-Americans (CEU) and 60 unrelated West Africans (YRI), were incorporated in the analysis as reference panels (phase 2, release 22, The HapMap Project; [27]).

We identified a list of selection-nominated candidate genes for testing against normal-range facial variation in admixed individuals of European and West African descent. Ancestry information and tests for accelerated evolution [28] were used to prioritize among a larger set of craniofacial genes. Since most genomic regions show low levels of allele frequency change across human populations, genes affecting traits that vary across populations are usually distinctive in showing large differences in frequency and other features of local variation and allele frequency spectra consistent with rapid local evolution. A preliminary set of craniofacial candidate genes was developed by searching the Online Mendelian Inheritance in Man (OMIM) database [24]. The keywords “craniofacial” and “facial” were searched to determine a set of genes known to affect craniofacial development. The OMIM entries for each gene included in the search output were then scanned manually to remove genes where the term appeared as a result of phrases such as “no craniofacial associations found” and other similar negative results. OMIM searching resulted in a list of 199 unique craniofacial candidate genes. Because this work focused on admixed populations of West African and European descent, the statistical power to detect linkage with craniofacial variation is greatest for SNPs that show large allele frequency differences between West African and European parental populations. Therefore, allele frequency differences among parental groups were further used to prioritize among the candidate genes. SNP frequency data in putative parental population (CEPH Europeans (CEU) and Yoruban (YRI) West Africans) for all SNPs within the 199 OMIM candidate genes were pulled from the HapMap database. This reduced subset of genes was then tested for signatures of non-neutral evolution in a 200 kb window surrounding each gene using a combination of three statistical tests: Locus-Specific Branch Length (LSBL) [29], the log of the ratio of the heterozygosities (lnRH) [30], and Tajima's D [31]. Because these tests are inferring different concepts regarding population history, we considered as significant any gene with statistical evidence of selection for all three measures or strong evidence of non-neutral evolution for two measures in either West African and/or European parental populations as a Selection-nominated candidate gene. It is notable that these steps were taken to increase the likelihood that a functional SNP would be available to test the ability of methods like BRIM to model individual gene effects on the human face. We are making no strong claims in this analysis that craniofacial genes generally or this subset in particular have been subject to greater than average levels of non-neutral evolution or that these genes do in fact have genetic variation that is affecting normal range facial variation in this sample. A total of 50 autosomal genes were thus selected (*SKI*, *LMNA*, *SIL1*, *EDN1*, *RSPO2*, *TRPS1*, *POLR1D*, *MAP2K1*, *ADAMTS10*, *TBX1*, *PEX14*, *HSPG2*, *CAV3*, *CTNND2*, *TFAP2A*, *PEX6*, *PEX3*, *MEOX2*, *RELN*, *ROR2*, *NEBL*, *CHUK*, *FGFR2*, *WT1*, *PEX16*, *BMP4*, *FANCA*, *RAI1*, *FOXA2*, *ECE1*, *DPYD*, *ZEB2*, *SATB2*, *FGFR3*, *NIPBL*, *NSD1*, *ENPP1*, *GLI3*, *COL1A2*, *BRAF*, *ASPH*, *FREM2*, *SNRPN*, *FBN1*, *MAP2K2*, *RPS19*, *DNMT3B*, *GDF5*, and *UFD1L*) and a set of SNPs with high allele frequency differences (delta >0.4) in these 50 craniofacial Selection-nominated candidate genes to test for associations with facial shape variation.

### 3D facial images and phenotyping

3D images composed of surface and texture maps were taken using the 3dMDface system (3dMD, Atlanta, GA). Participants were asked to close their mouths and hold their faces with a neutral expression for the picture. Images were then exported from the 3dMD Patient software in OBJ file format and imported into a scan cleaning program for cropping and trimming, removing hair, ears, and any dissociated polygons. The complete work flow involved in processing face scans is depicted in Figure 1. Five positioning landmarks were placed on the face to establish a rough facial orientation. Subsequently, an anthropometric mask (7,150 quasi-landmarks) was non-rigidly mapped onto the original 3D images and their reflections [8], [9], [17], which were constructed by changing the sign of the x-coordinate [18], [32]. This established homologous spatially-dense quasi-landmark (Q-L) configurations for all original and reflected 3D images (*8*). Note that, by homologous, we mean that each quasi-landmark occupies the same position on each face relative to all other quasi-landmarks. Subsequently, a generalized Procrustes superimposition [18], [33] is used to eliminate differences in position, orientation, and scale of both original and reflected configurations combined was performed. This constructed a tangent space of the Kendall shape-space centered on the overall consensus configuration [25]. Procrustes shape coordinates, representing the shape of an object [34], were obtained for all 3D faces and their reflections. After Procrustes superimposition, the overall consensus configuration is perfectly symmetrical and a single shape can be decomposed into its asymmetric and its bilaterally symmetric part [18]. The average of an original and its reflected configuration constitutes the symmetric component while the difference between the two configurations constitutes the asymmetric component [19], [35]. The analyses in this report were all based on facial shape as represented using the component of symmetry only. Although deviations from bilateral symmetry are thought to be the effects of developmental noise and/or environmental factors [36], it is likely there are genetic effects on asymmetry, which would compel independent investigation.

Principal components analysis (PCA) [9] on the superimposed and symmetrized quasi-landmark configurations of the panel of 592 participants resulted in 44 PCs that together summarize 98% of the total variation in face space. To examine the effect of excluding lower PCs, we first reconstructed actual quasi-landmark configuration from the 44 PCs only and compared these to the original remapped face. We found that the average root mean squared error (RMSE) is as small as 0.2 mm per quasi-landmark. The localized differences between the original faces and the faces as represented by the first 44 PCs are largest around the iris, eyelids, under the nose, and the corners and opening of the mouth and are at most about 0.45 mm. How a PC or any other independent variable affects the face can be shown with heat maps and shape transformations: heat maps use contrasting colors to highlight the specific parts of the face that are affected, while shape transformations illustrate the changes in overall face shape with two or more images of the face at set intervals. Shape transformations are obtained from the average face in the direction of each PC at −3 and +3 times the accompanying standard deviation (square-root of the eigenvalue). Figure 2 shows how the first 10 PCs affect the face. Some of these PCs (*e.g.*, PC1, PC2, PC3) summarize effects on many parts of the face, while other PCs (*e.g.*, PC4, PC5) summarize the effects of changes in only particular parts of the face. The effects of each of the 44 PCs as well as the RIP variables can be visualized with a GUI software tool that we have written called DNA2FACEIN3D.EXE. The program and instruction manual can be downloaded here: http://tinyurl.com/DNA2FACEIN3D.

We have used three methods to visualize and quantify facial difference so that we can systematically express the effects of particular response-based imputed predictor (RIP) variables on the face into anatomically interpretable results. These are based on comparing faces pairwise, such as comparing the most feminine RIP-S to the most masculine RIP-S transformed consensus faces using three fundamental measures: area ratio, normal displacement, and curvature ratio. These two ratios and one displacement along with particular inter-landmark distances and angles can together be termed “face shape change parameters” (FSCPs) and are a means of translating face shape changes from the abstract face space into language of facial characteristics such that comparisons between clinical or anthropological descriptions of faces can be compared to bootstrapped response-based imputation modeling (BRIM) results. The statistical significance of these FSCPs can be estimated using permutation. A more detailed description on how this is done is given in the supplementary online material.

### Human observer ratings and judgments

#### Ancestry and sex observations

Given the dexterity humans have for discerning numerous traits, features, and expressions, it is reasonable to expect the observer would provide a useful reference point for studies of the genetics of facial traits. We accessed observer ratings and judgments of sex and ancestry in order to test the informativeness of RIP-A and RIP-S.

#### Selection of stimuli

A total of 500 participant faces were selected and divided into twenty-five panels of twenty faces, with each panel including faces of research participants across the range of genomic ancestry levels and similar numbers of male and female faces. We used false colored grey GIF animations so that ancestry and sex ratings and judgments would be based on face-shape cues but not cues of skin, iris, or hair pigmentation or hair texture. Animation order was randomized.

#### Administration of instruments

We administered the instruments containing the animated, false-colored GIFs with accompanying questions using Survey Monkey (SurveyMonkey.com LLC; Palo Alto, CA). Four survey questions were asked for each of 20 faces participants observed:

1. “What proportion (from 0% to 100%) of this person's ancestry appears to be West African?” *(Ratings made with a number between 0 and 100.)*
2. Which single categorical group best describes this person? *(Judged with Black African, or African-American; White, European or European-American; or Mixed)*
3. Does this person appear to be male or female? *(Judged with “male” or “female”)*
4. “How feminine does this person's face appear to you?” *(Ratings made with a choice from a 7-point Likert scale ranging from 1 “extremely feminine” to 7 “extremely masculine”.)*

Observers were randomly assigned to one of the 20 panels through a link on the Anthropology Department homepage. Observers were recruited from students enrolled at Penn State University. Of the 1,156 participants, 938 (81.1%) completed the surveys. The number of observers for the 20 alternative surveys ranged from 27 to 70, with a mean of 47. Observers who completed fewer than half of the survey as well as three whose discrepancies were more than three standard deviations from the mean were excluded from the analysis. Observers were not trained and were not familiar with the research participants whose faces were shown as stimuli.

## Supporting Information

Figure S1 Response-based imputation based on Leave-One-Out.
(TIFF)

Figure S2 Response-based imputation using distance decomposition.
(TIFF)

Figure S3 Bootstrapping.
(TIFF)

Figure S4 The correlation of A′ with A in function of the magnification constant c. The higher the constant, higher the level of injected noise, and hence, the lower the correlation.
(TIFF)

Figure S5 Absolute correlations of retrieved RIP-A′ variables for each iteration and for each noise level with the genomic ancestry variable A. Color bar ranges from 0 to 1. Note the peak correlation of 1 in the situation of no noise injection and no iteration, this correlation is of A against itself.
(TIFF)

Figure S6 Correlations of retrieved RIP-A′ variables for each iteration and for each misclassification level with RIP-A. Color bar ranges from 0 to 1.
(TIFF)

Figure S7 Correlation of A′ and RIP-A′ with A for different levels of noise.
(TIFF)

Figure S8 Correlation of A′ and RIP-A′ with RIP-A for different levels of noise.
(TIFF)

Figure S9 Correlation matrices for different AIMs subsets over each iteration 1–6 (A–F).
(TIFF)

Figure S10 Correlation matrices of AIM 68 for different population subsamples over each iteration 0–6 (A–F) Note that iteration 0, implies correlations in between the original predictor variables. A = American, B = Brazilian, C = Cape Verdean.
(TIFF)

Figure S11 Correlation matrices of M-index for different population subsamples over each iteration 0–6 (A–F) Note that iteration 0, implies correlations in between the original predictor variables. A = American, B = Brazilian, C = Cape Verdean.
(TIFF)

Figure S12 AUC in function of percentage misclassification.
(TIFF)

Figure S13 Average AUC values of ROC analyses for each iteration and for each level of misclassification.
(TIFF)

Figure S14 AUC of S′ and RIP-S′ with S as grouping variable in an ROC analysis for different levels of misclassification.
(TIFF)

Figure S15 Correlation boxplots of RIP-G values for each iteration with genomic ancestry A, without ancestry conditioning.
(TIFF)

Figure S16 Correlation boxplots of RIP-G values for each iteration with RIP-A, without ancestry conditioning.
(TIFF)

Figure S17 Correlation boxplots of RIP-G values for each iteration with A, conditioned on A.
(TIFF)

Figure S18 Correlation boxplots of RIP-G values for each iteration with RIP-A, conditioned on A.
(TIFF)

Figure S19 Correlation boxplots of RIP-G values for each iteration with A, conditioned on RIP-A.
(TIFF)

Figure S20 Correlation boxplots of RIP-G values for each iteration with RIP-A, conditioned on RIP-A.
(TIFF)

Figure S21 Correlation boxplots of RIP-G values for each iteration with S, without sex conditioning.
(TIFF)

Figure S22 Correlation boxplots of RIP-G values for each iteration with RIP-S, without sex conditioning.
(TIFF)

Figure S23 Correlation boxplots of RIP-G values for each iteration with S, conditioned on S.
(TIFF)

Figure S24 Correlation boxplots of RIP-G values for each iteration with RIP-S, conditioned on S.
(TIFF)

Figure S25 Correlation boxplots of RIP-G values for each iteration with A, conditioned on RIP-A.
(TIFF)

Figure S26 Correlation boxplots of RIP-G values for each iteration with RIP-A, conditioned on RIP-A.
(TIFF)

Figure S27 The effect of the rs13267109 SNP in *FGFR1* using approach 1 (no conditioning for ancestry), left, approach 2 (conditioning for genomic ancestry), second from left, approach 3 (conditioning for RIP-A), second from right and approach 4 (conditioning for RIP-A using BRIM), right.
(TIFF)

Figure S28 Facial Regions: (A) Orange, Orbital Ridges; Red, Lips; Yellow, Eyes Superior; Green. Eyes Iinferior; Blue, Paranasal Tissues; Pink, Chin. (B) Orange, Left Eye; Red, Right Eye; Yellow, Lower Lip; Green, Upper Lip; Blue Cheek Bones. (C) Orange, Cheeks; Pink, Nose ridge; Yellow, Browridges; Green, Lateral half of eyes; Blue; Medial half of eyes; Red, Cheekbones. (D) Green, Forehead; Yellow, Midface; Red, Nasal tip; Pink, Philtrum; Orange, Nasal bridge; Blue, Lower face. (E) Green, Metopic ridge; Orange, Forehead sides; Yellow, Eyes; Red, Nose; Blue, Malars; Pink, Lateral midface.
(TIFF)

Figure S29 Manually annotated landmarks: gl = Glabella; pn = Pronasale; sn = Subnasale; ls = Labiale Superiusinferius; li = Labiale Iinferius; gn = Gnathion; exr = Right Eendocanthion; pr = Right Ppupil; pl = Left Ppupil; enl = Left Eendocanthion; ar = Right Alar; chr = Chelion right; chl = Chelion left.
(TIFF)

Figure S30 Measurement of facial squareness: The facial border is projected onto the XY plane and compared to a fitted square. The result is a dissimilarity score with a higher/lower score indicating a less/more square face. The colors in the border points indicate their distance to the square.
(TIFF)

Figure S31 A proxy for head circumference: The forehead intersection with a plane halfway the Glabella and the top of the forehead is determined. This generates and arc segment through which a circle is fitted. The radius of the fitted circle serves as a proxy for head circumference.
(TIFF)

Figure S32 Receiver operator curve (ROC) showing the ability of facial femininity (RIP-S) to correctly classify faces by self-reported sex.
(TIFF)

Figure S33 The effect, effect-size (r^2^), significance of the effect (H), and two shape transformations at opposite sides (+3 and −3 times the standard deviation) of the RIP distribution for sex (top row) and ancestry (bottom row). The maximum values for r^2^ can be found in Table S3.
(TIFF)

Figure S34 The effect, effect-size (r^2^), significance of the effect (H), and two shape transformations at opposite sides (+X and −X times the standard deviation) of the RIP distribution. The maximum values for R^2^ can be found in Table S3. PART 1.
(TIFF)

Figure S35 The effect, effect-size (r^2^), significance of the effect (H), and two shape transformations at opposite sides (+X and −X times the standard deviation) of the RIP distribution. The maximum values for R^2^ can be found in Table S3. PART 2.
(TIFF)

Figure S36 Facial area changes due to sex and ancestry.
(TIFF)

Figure S37 Facial curvature changes due to sex and ancestry.
(TIFF)

Figure S38 Normal displacements due to sex and ancestry.
(TIFF)

Figure S39 Facial area changes due to candidate genes. PART 1.
(TIFF)

Figure S40 Facial area changes due to candidate genes. PART 2.
(TIFF)

Figure S41 Facial curvature changes due to candidate genes. PART 1.
(TIFF)

Figure S42 Facial curvature changes due to candidate genes. PART 2.
(TIFF)

Figure S43 Normal displacements due to candidate genes. PART 1.
(TIFF)

Figure S44 Normal displacements due to candidate genes. PART 2.
(TIFF)

Table S1 List of local face shape change parameters (FSCPs) measured.
(DOCX)

Table S2 ANOVA analysis of genotypes on facial morphology using RIP variables. The Table is sorted from low to high p-value on the three group ANOVA results.
(DOCX)

Table S3 RIP effect-size statistics.
(DOCX)

Table S4 Empirical *p*-values under 10,000 permutations for the local FSCPs listed in TableS1 tested for the 24 candidate genes, sex and ancestry.
(DOCX)

Text S1 Supporting materials text (1) Bootstrapped response = based imputation modeling (BRIM), (2) Empirical Analysis of BRIM, (3) Facial Characteristics, (4) Extended Results: Sex, Ancestry, and Gene Effects.
(DOCX)
