# Supplementary figures and images for: Modeling 3D Facial Shape from DNA
Source: PLoS Genet. 2014 Mar 20;10(3):e1004224. doi: 10.1371/journal.pgen.1004224 (PMC3961191; doi:10.1371/journal.pgen.1004224)

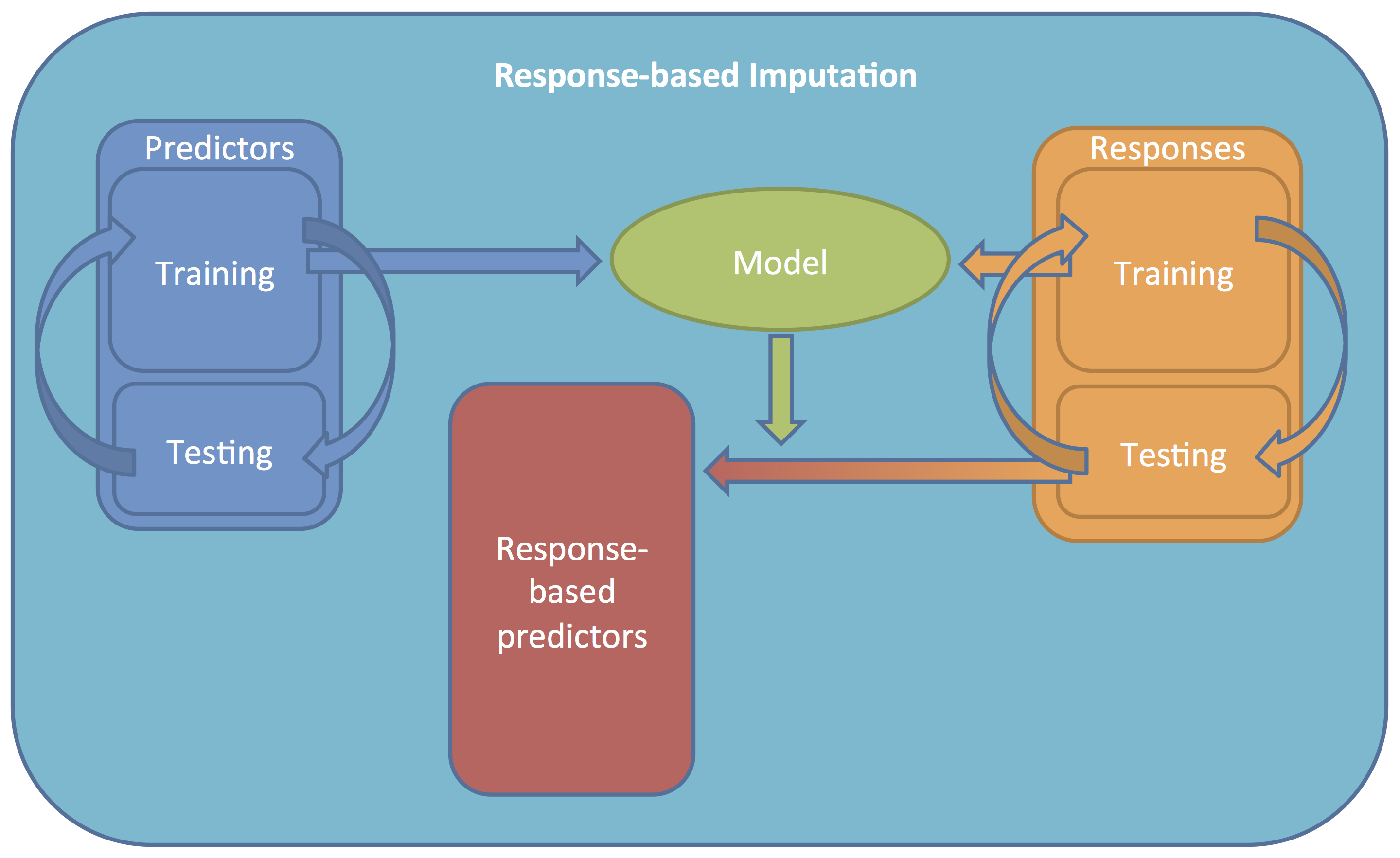

Supplement: Figure S1 — Response-based imputation based on Leave-One-Out. (TIFF) [file pgen.1004224.s001.tiff]

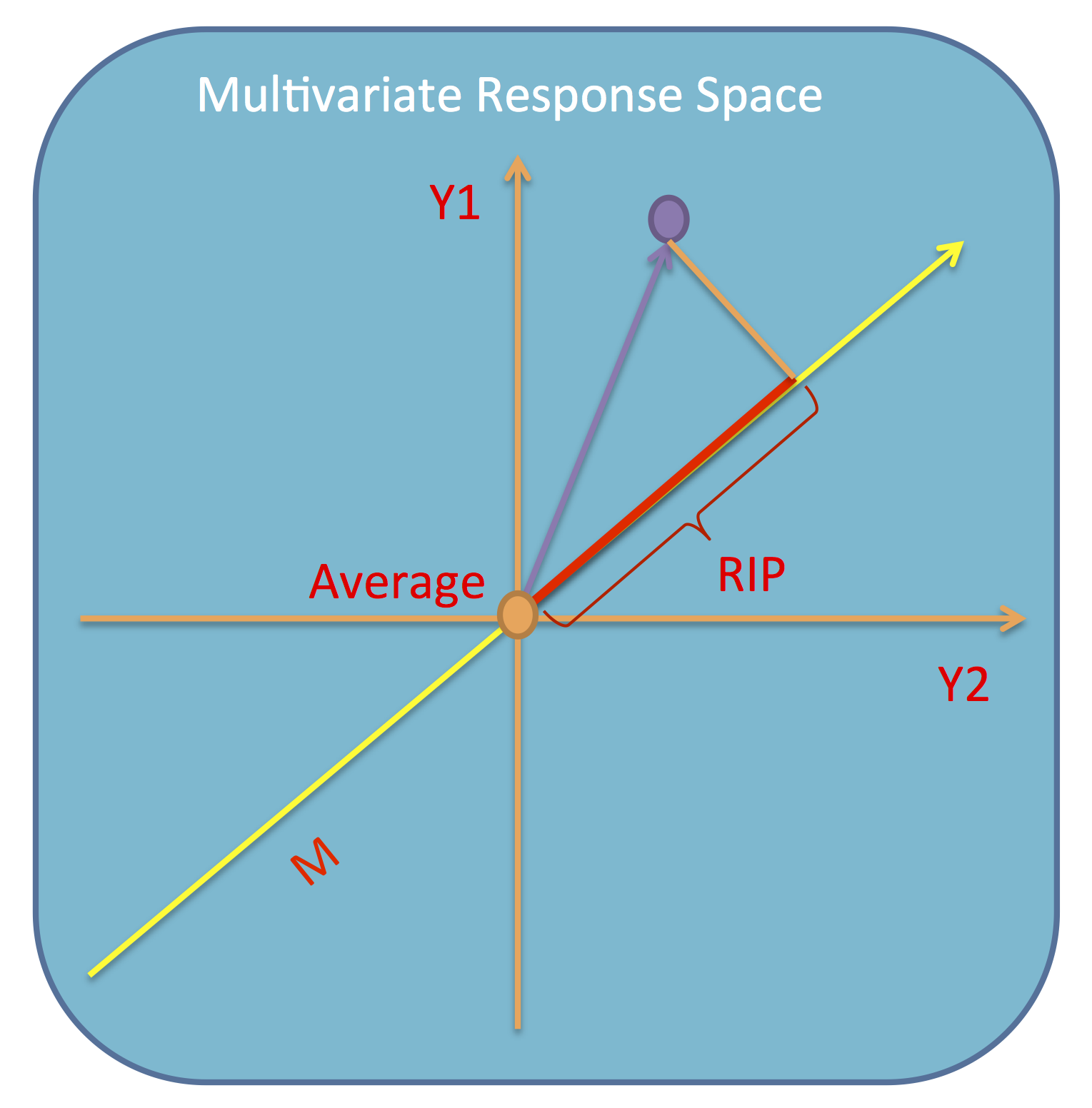

Supplement: Figure S2 — Response-based imputation using distance decomposition. (TIFF) [file pgen.1004224.s002.tiff]

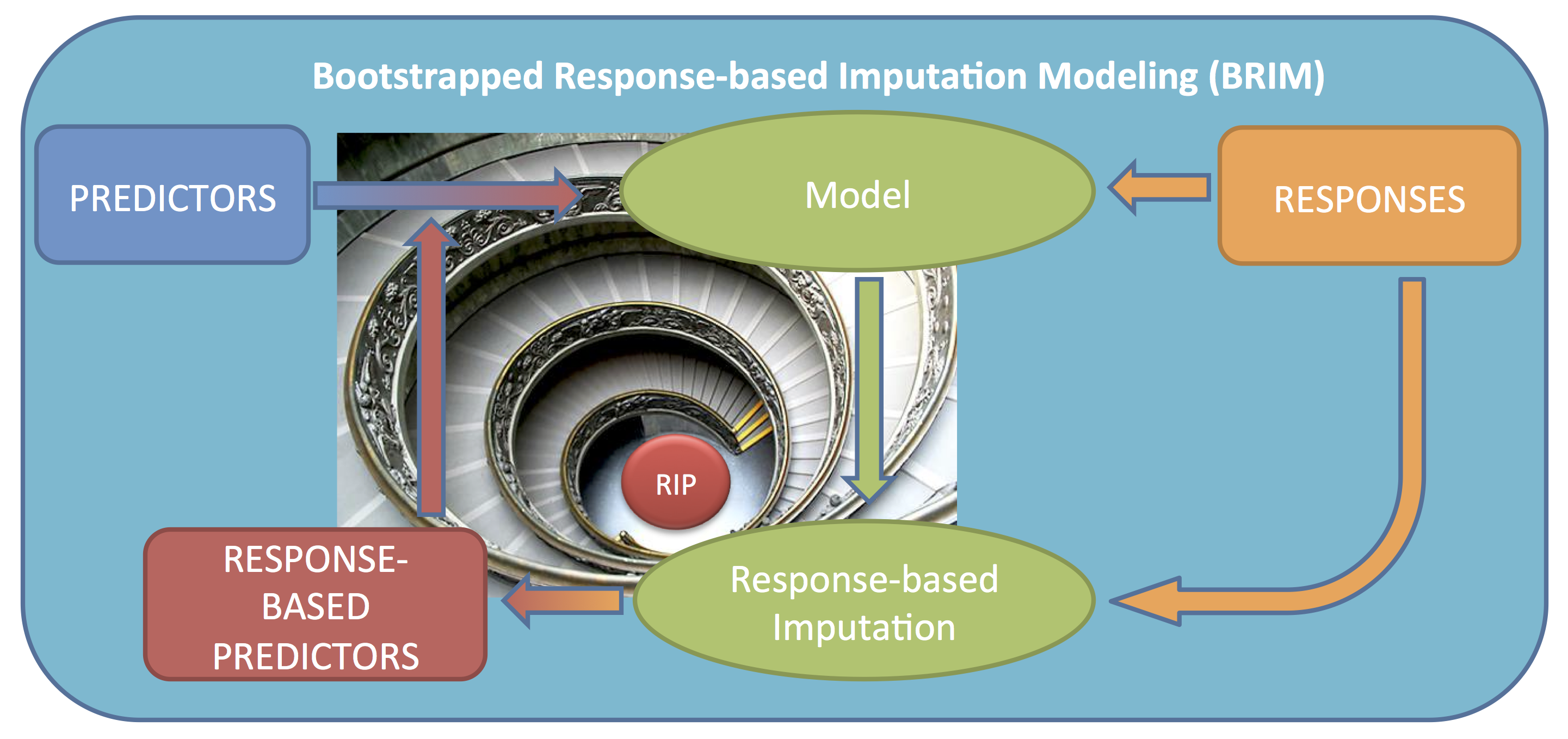

Supplement: Figure S3 — Bootstrapping. (TIFF) [file pgen.1004224.s003.tiff]

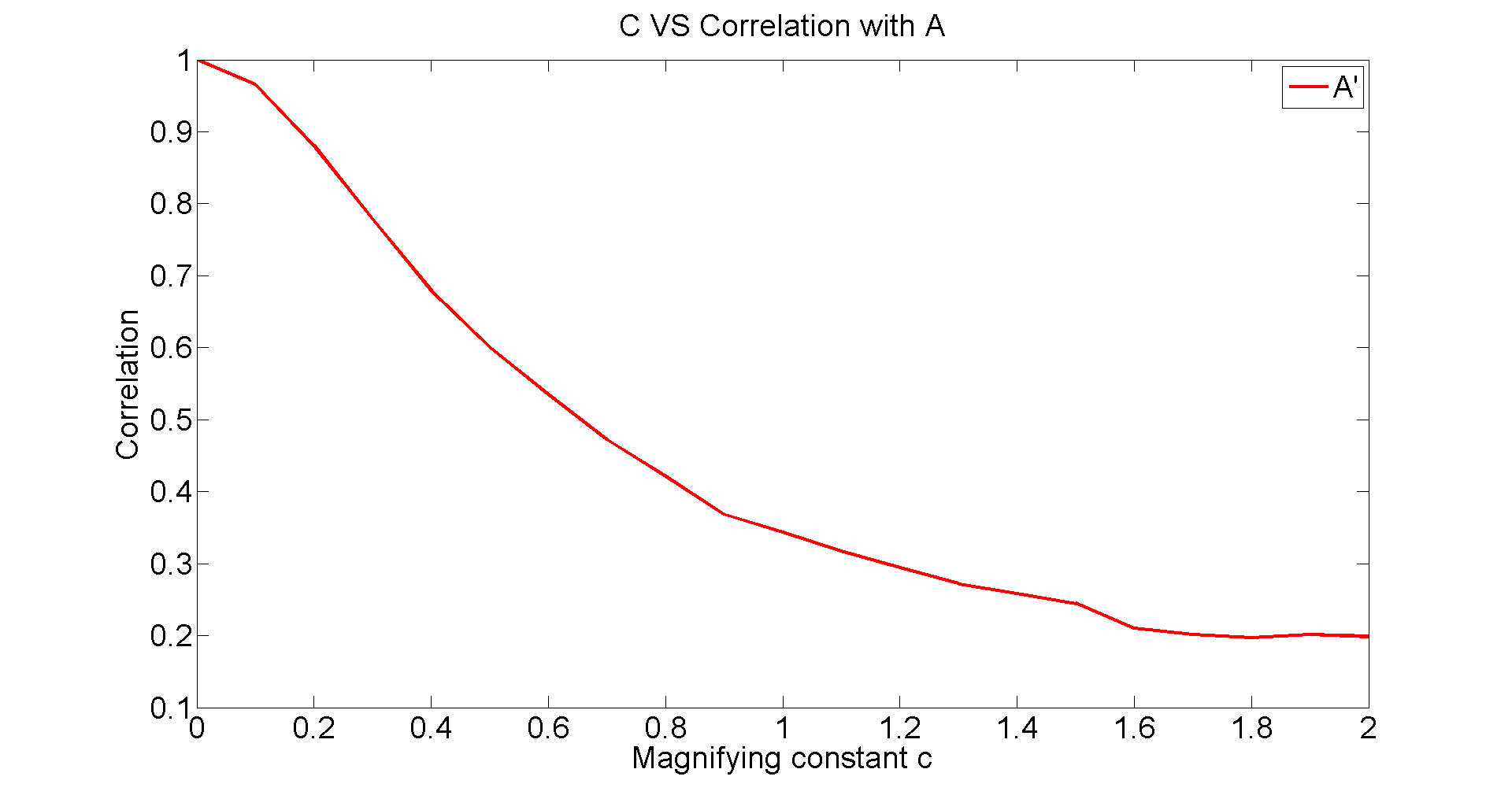

Supplement: Figure S4 — The correlation of A′ with A in function of the magnification constant c. The higher the constant, higher the level of injected noise, and hence, the lower the correlation. (TIFF) [file pgen.1004224.s004.tiff]

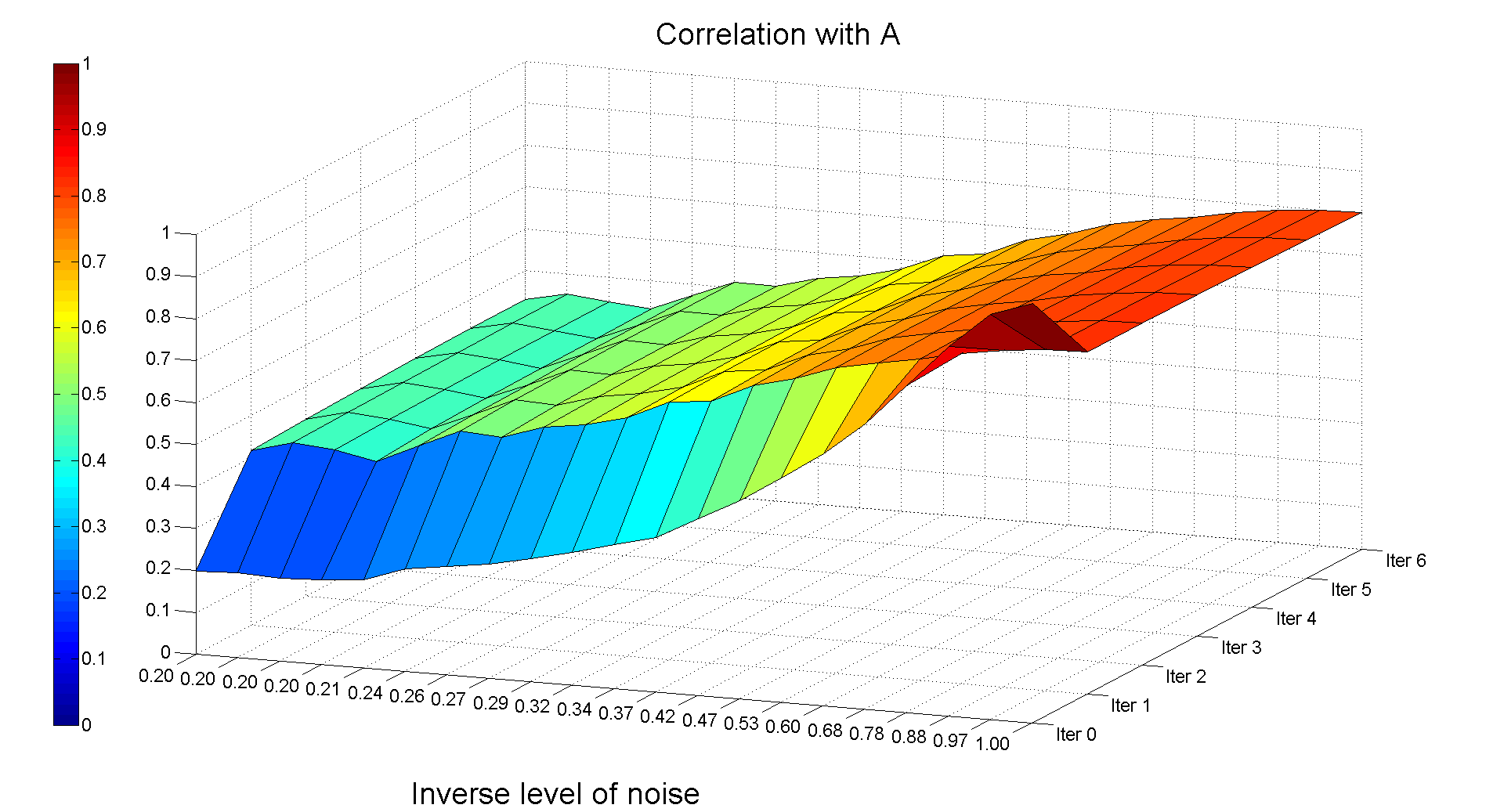

Supplement: Figure S5 — Absolute correlations of retrieved RIP-A′ variables for each iteration and for each noise level with the genomic ancestry variable A. Color bar ranges from 0 to 1. Note the peak correlation of 1 in the situation of no noise injection and no iteration, this correlation is of A against itself. (TIFF) [file pgen.1004224.s005.tiff]

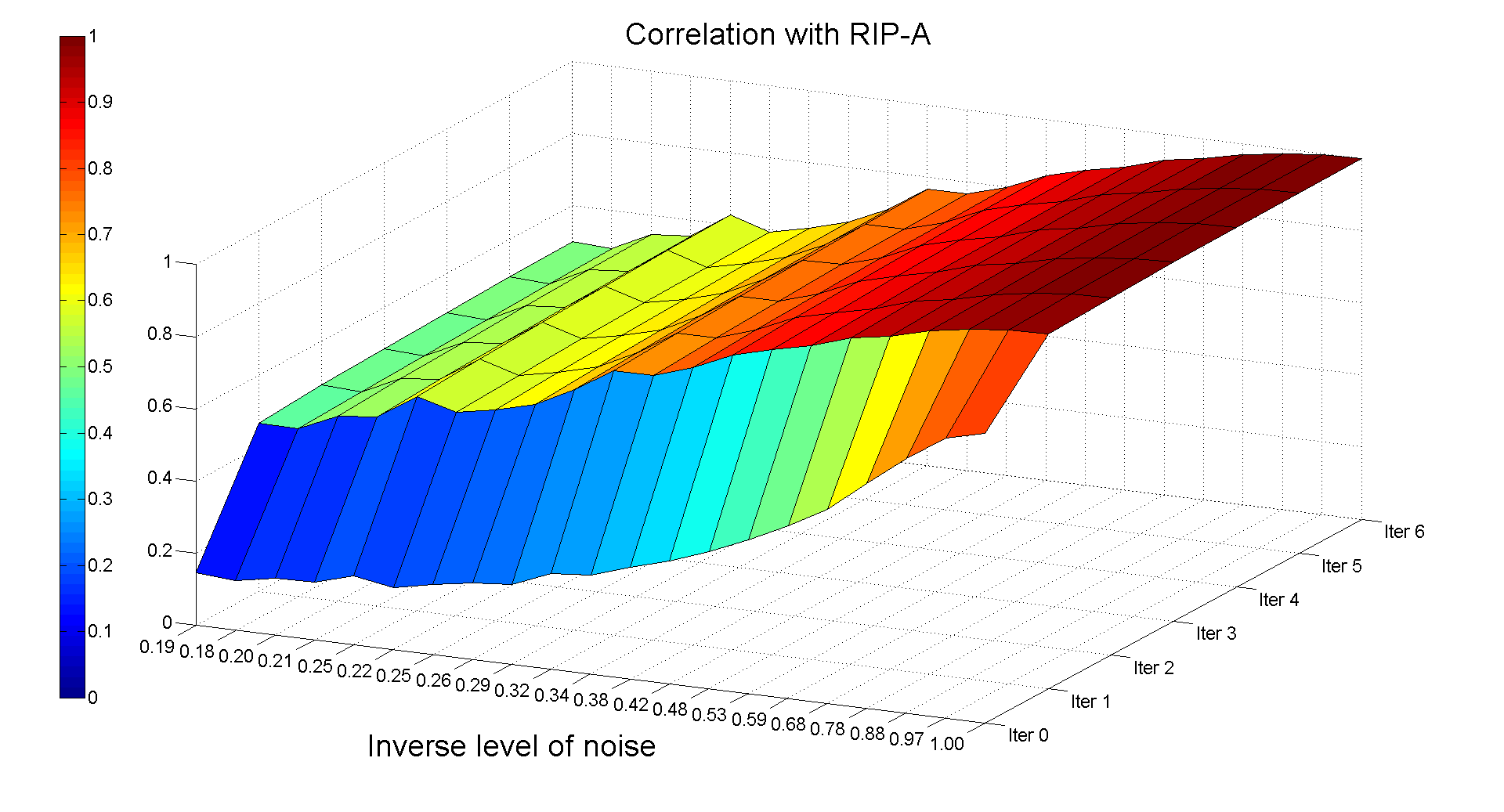

Supplement: Figure S6 — Correlations of retrieved RIP-A′ variables for each iteration and for each misclassification level with RIP-A. Color bar ranges from 0 to 1. (TIFF) [file pgen.1004224.s006.tiff]

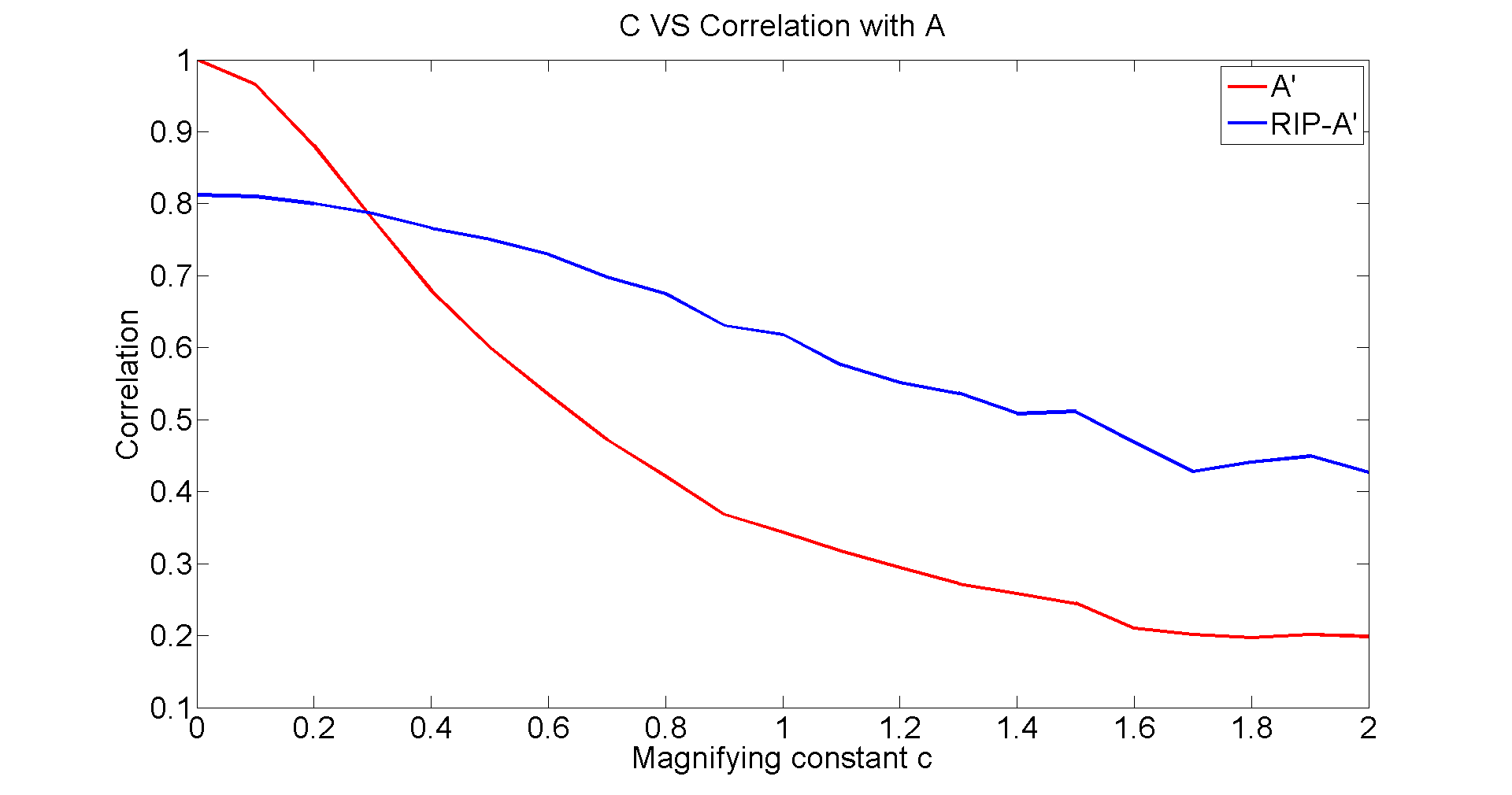

Supplement: Figure S7 — Correlation of A′ and RIP-A′ with A for different levels of noise. (TIFF) [file pgen.1004224.s007.tiff]

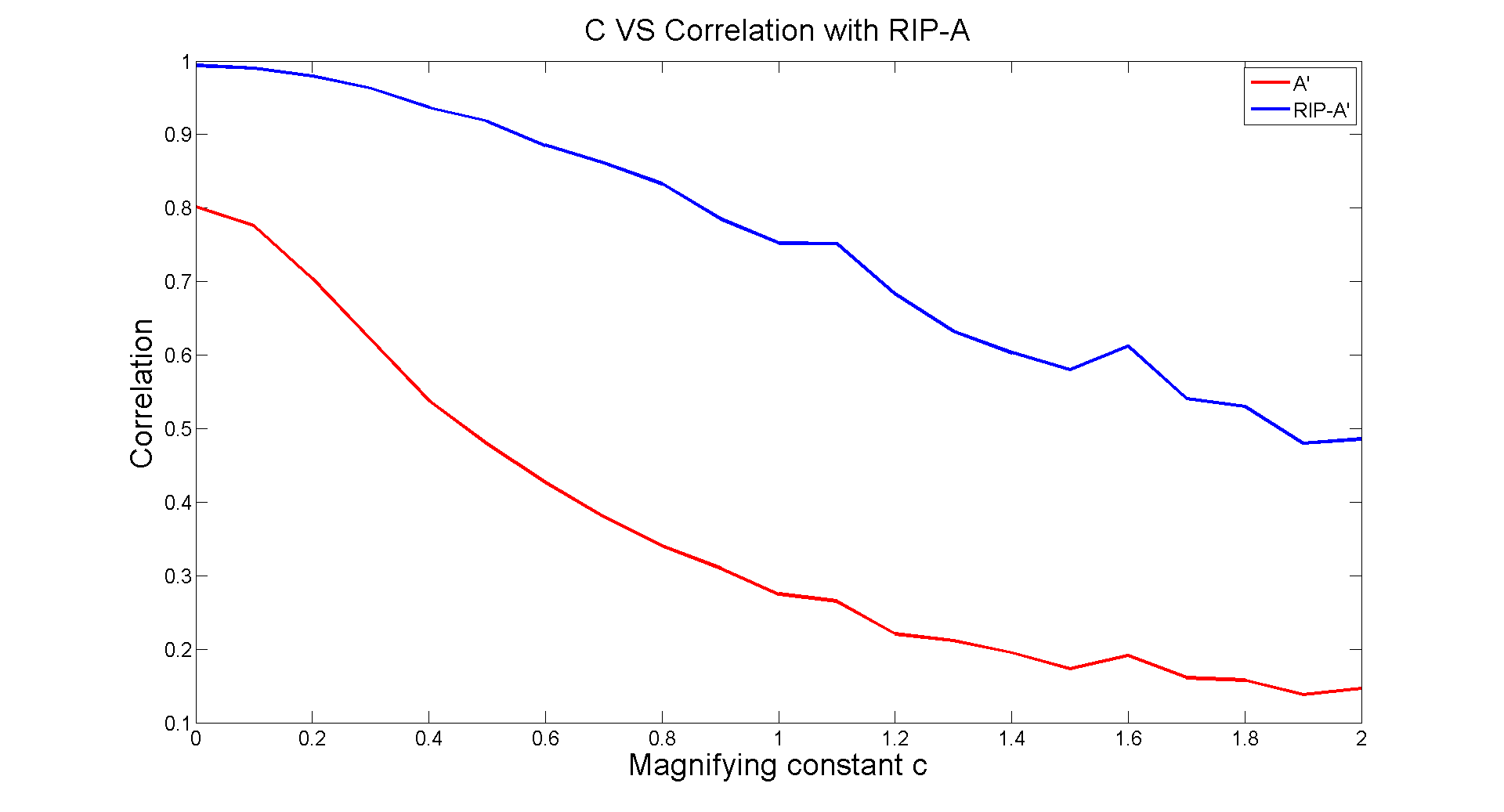

Supplement: Figure S8 — Correlation of A′ and RIP-A′ with RIP-A for different levels of noise. (TIFF) [file pgen.1004224.s008.tiff]

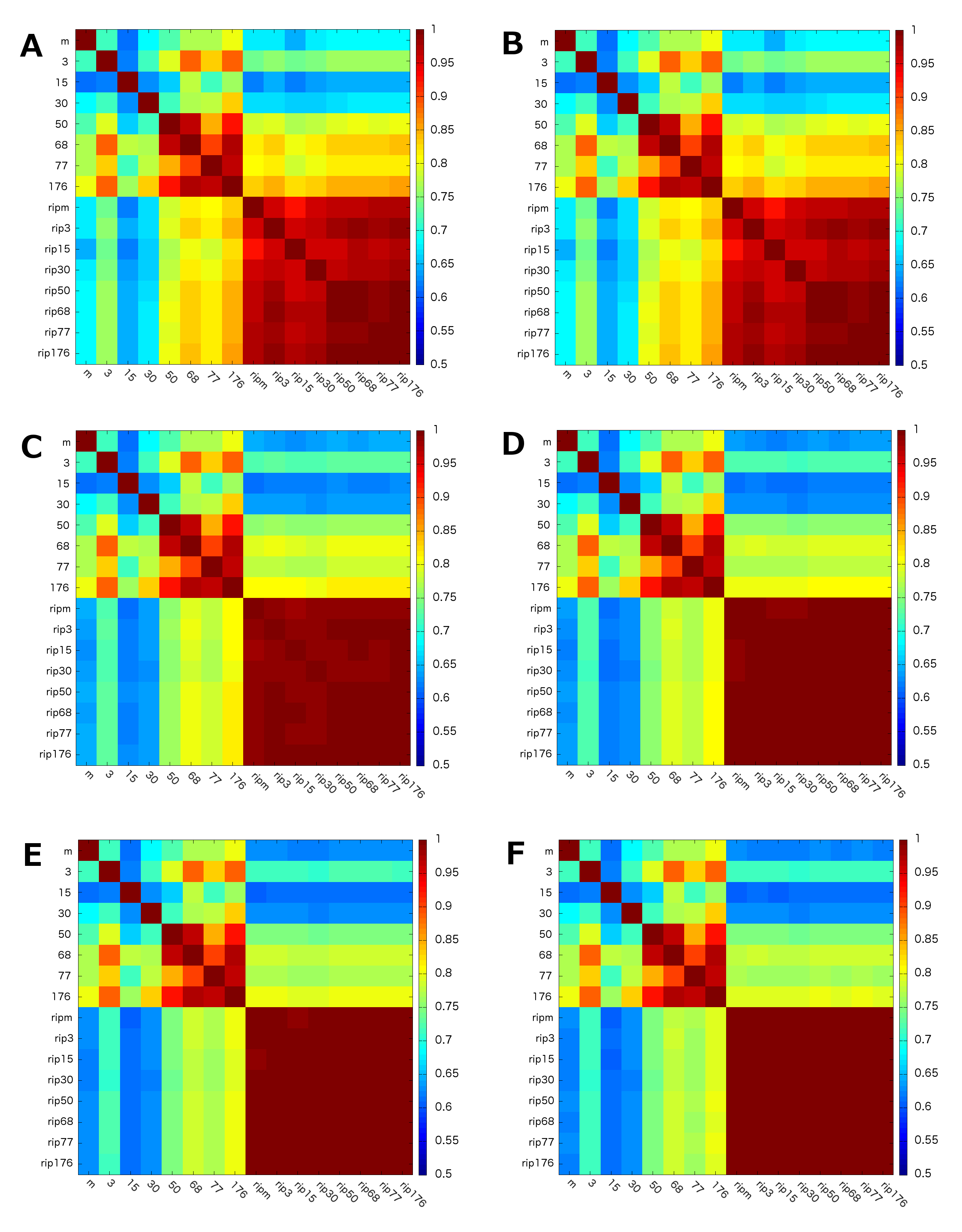

Supplement: Figure S9 — Correlation matrices for different AIMs subsets over each iteration 1–6 (A–F). (TIFF) [file pgen.1004224.s009.tiff]

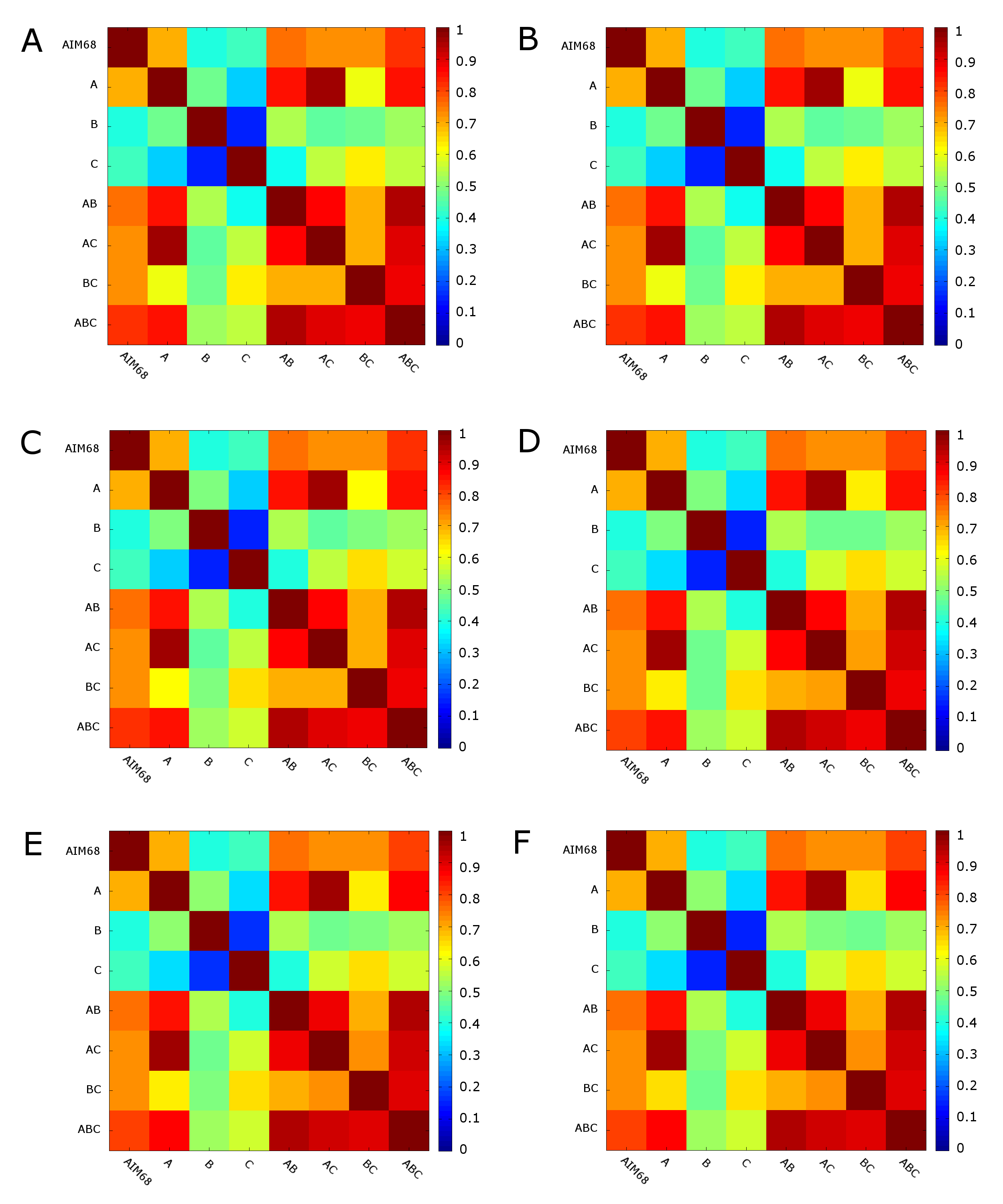

Supplement: Figure S10 — Correlation matrices of AIM 68 for different population subsamples over each iteration 0–6 (A–F) Note that iteration 0, implies correlations in between the original predictor variables. A = American, B = Brazilian, C = Cape Verdean. (TIFF) [file pgen.1004224.s010.tiff]

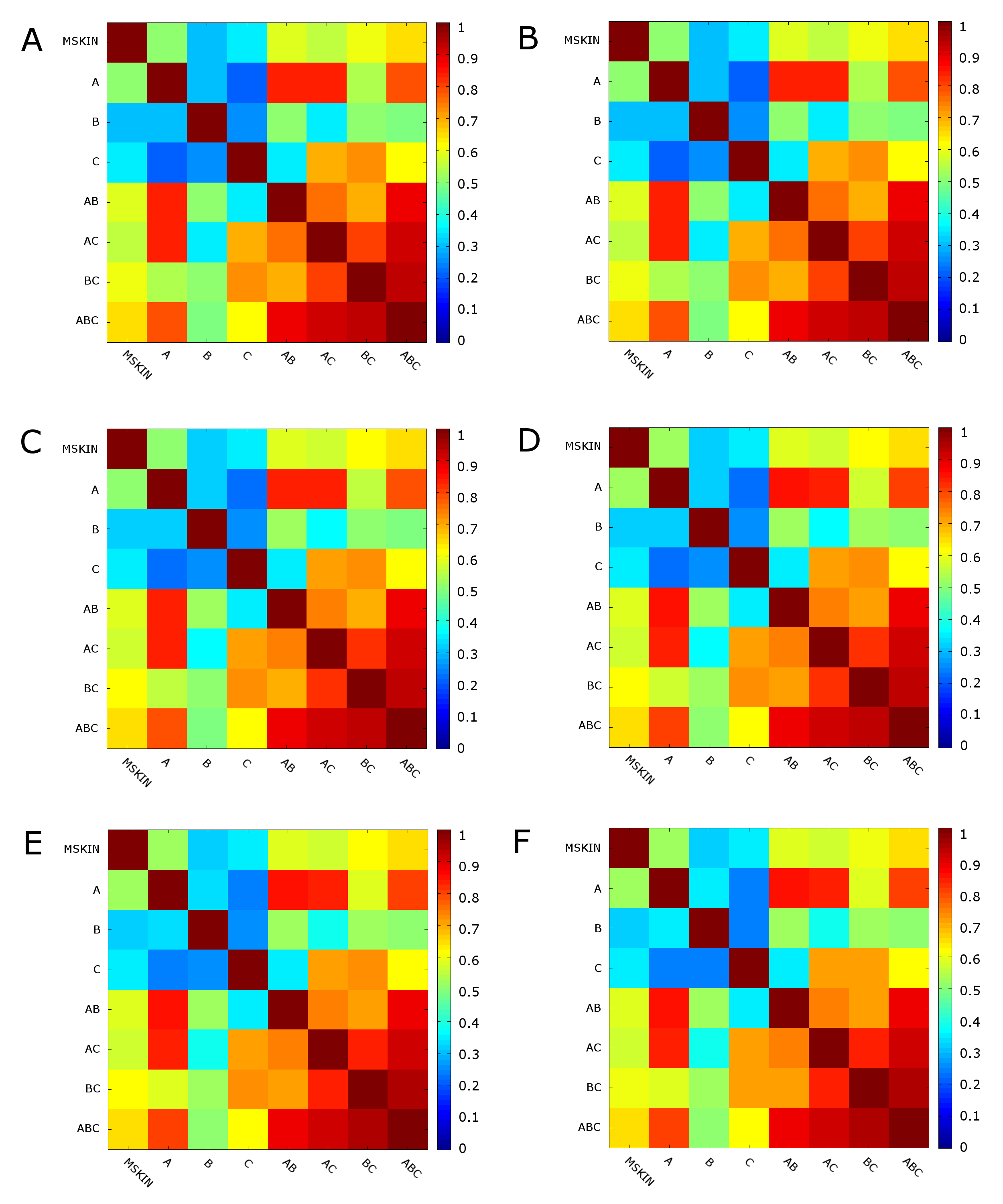

Supplement: Figure S11 — Correlation matrices of M-index for different population subsamples over each iteration 0–6 (A–F) Note that iteration 0, implies correlations in between the original predictor variables. A = American, B = Brazilian, C = Cape Verdean. (TIFF) [file pgen.1004224.s011.tiff]

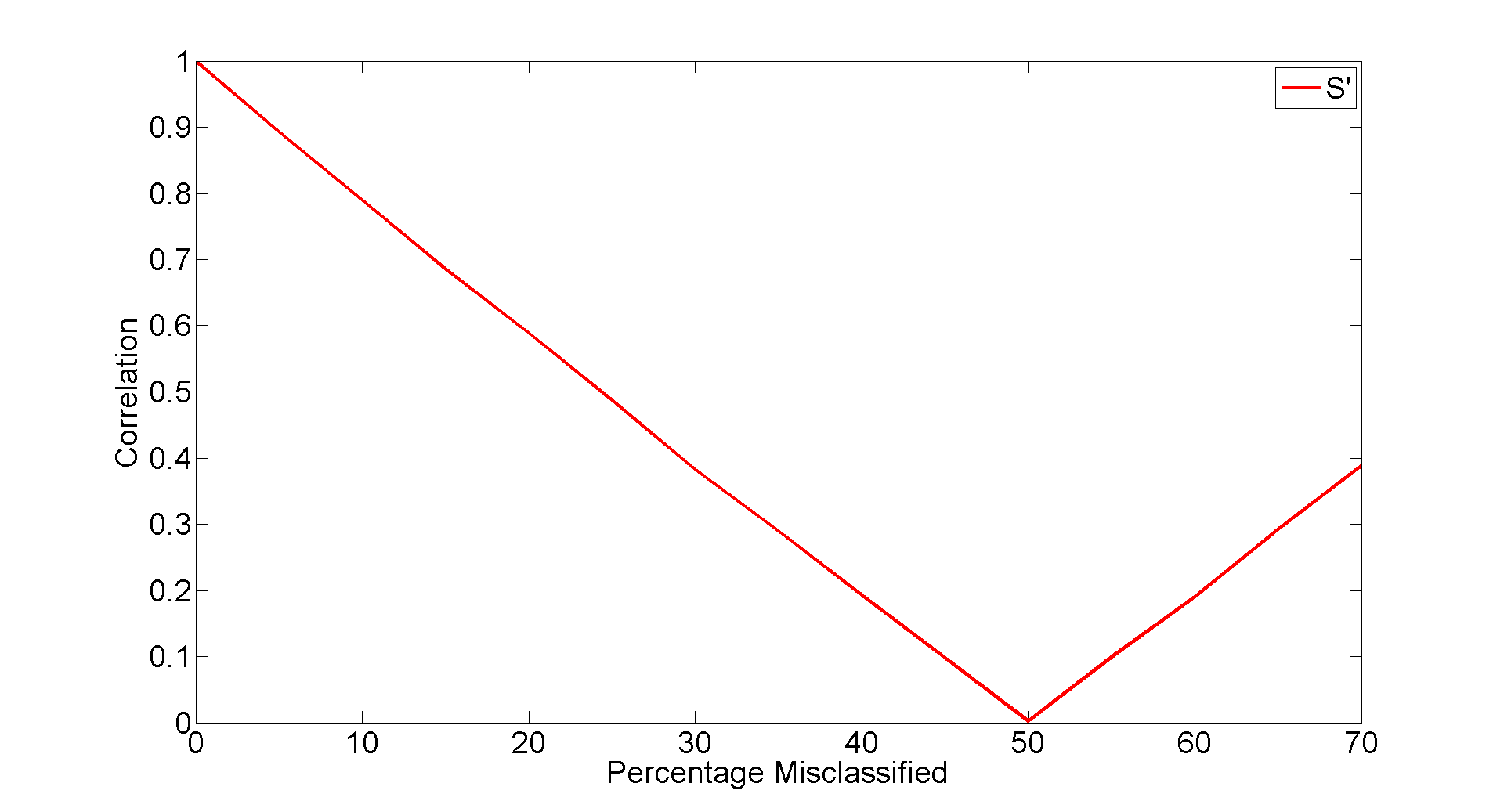

Supplement: Figure S12 — AUC in function of percentage misclassification. (TIFF) [file pgen.1004224.s012.tiff]

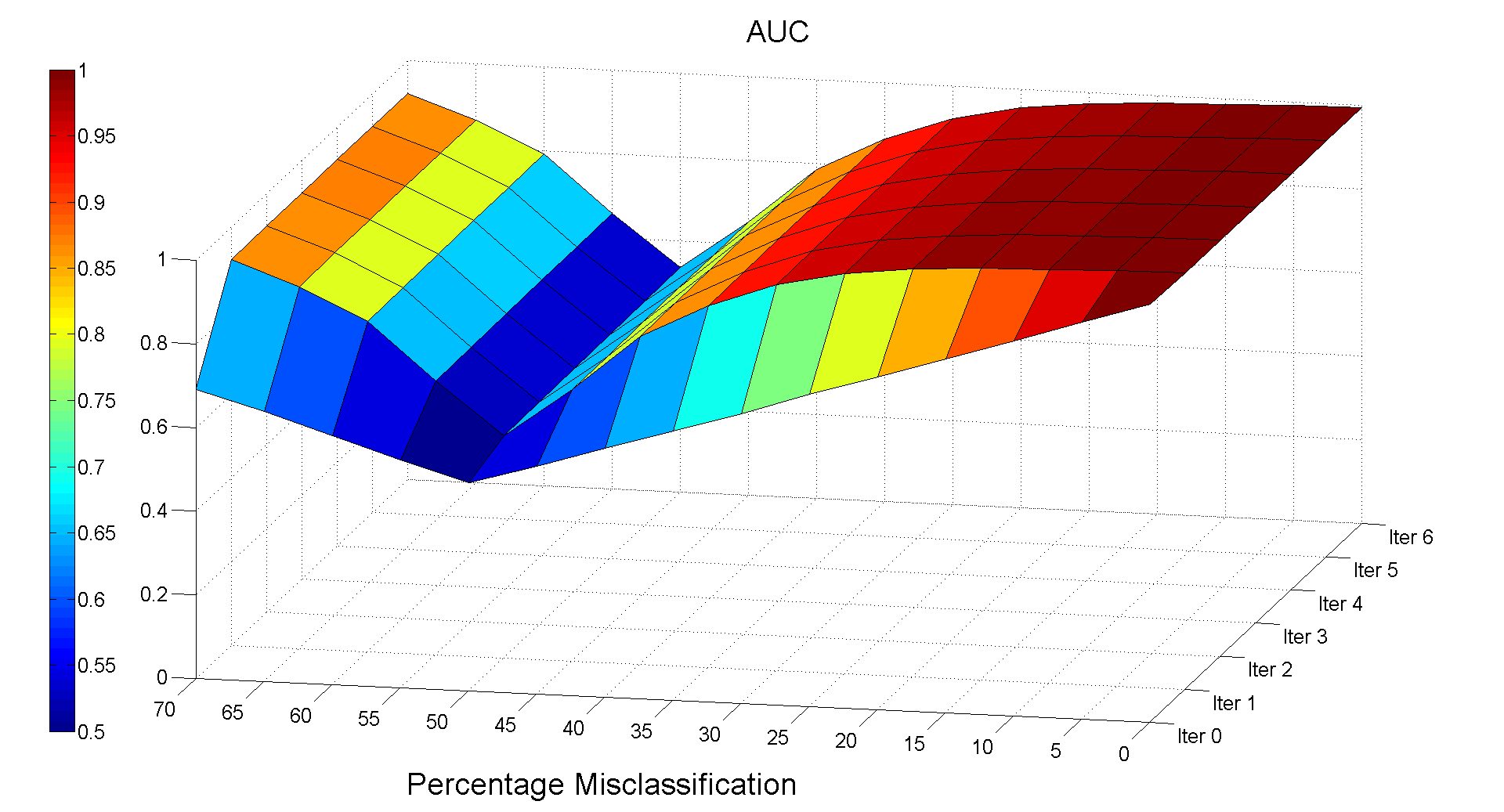

Supplement: Figure S13 — Average AUC values of ROC analyses for each iteration and for each level of misclassification. (TIFF) [file pgen.1004224.s013.tiff]

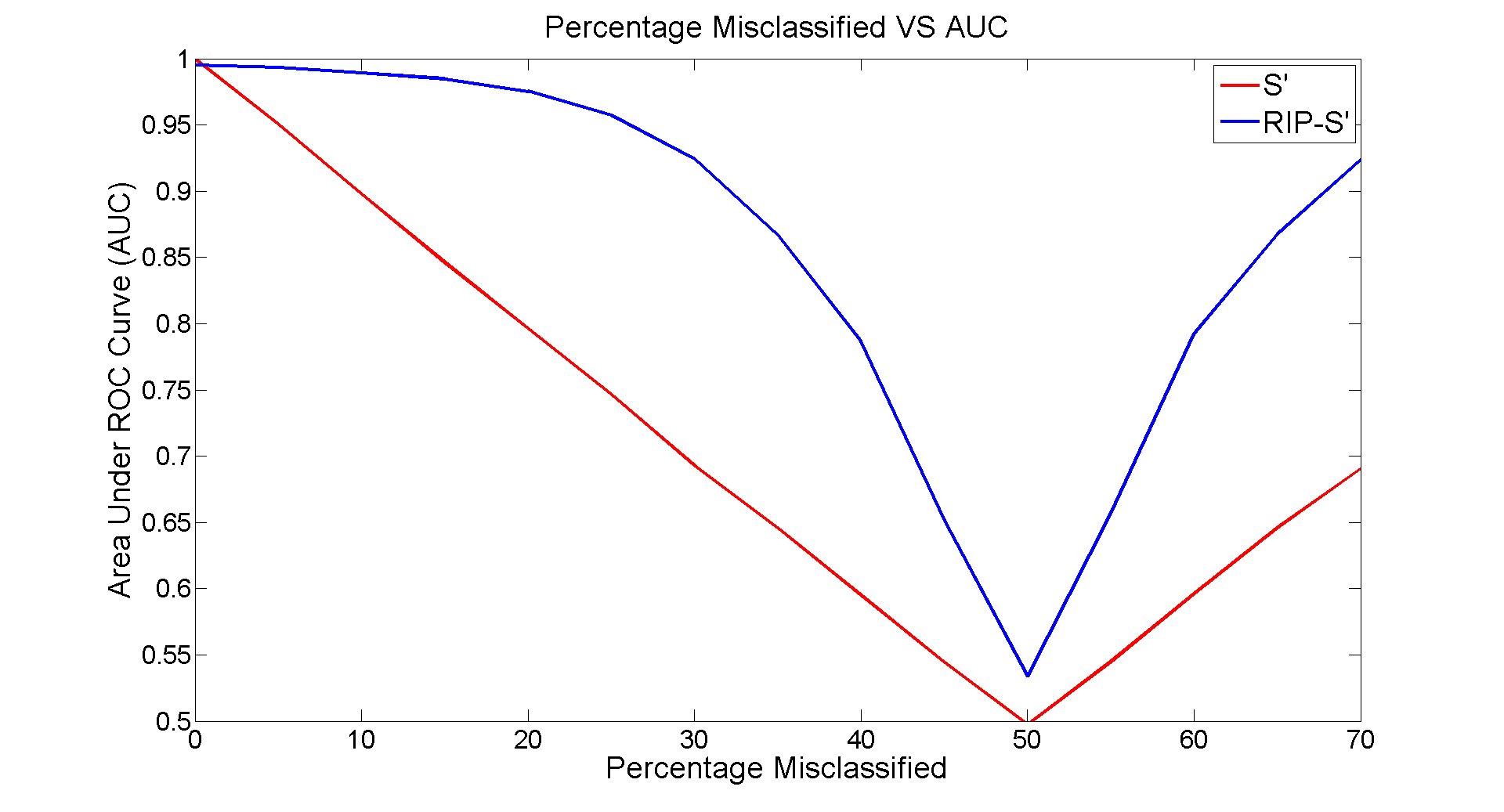

Supplement: Figure S14 — AUC of S′ and RIP-S′ with S as grouping variable in an ROC analysis for different levels of misclassification. (TIFF) [file pgen.1004224.s014.tiff]

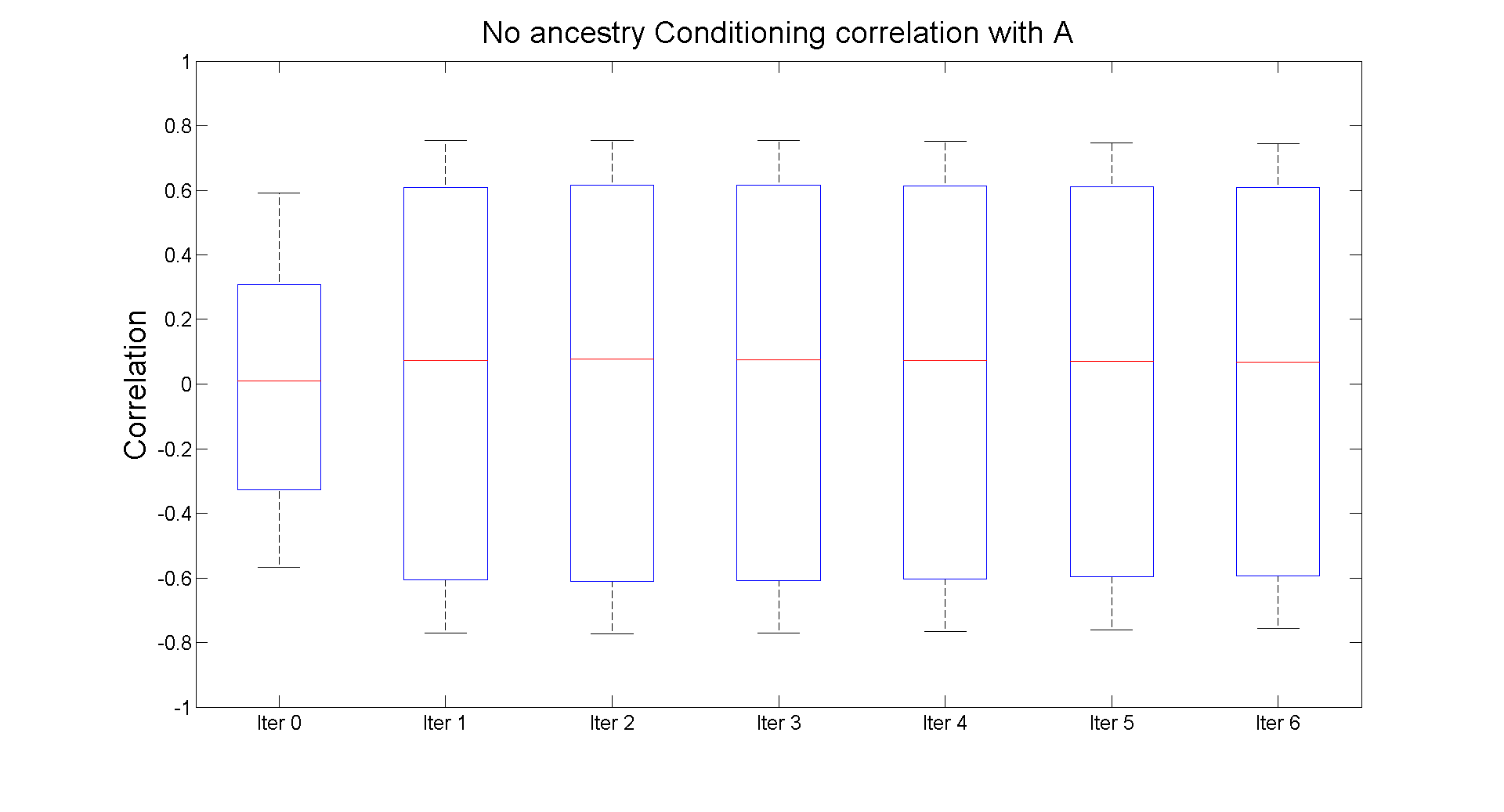

Supplement: Figure S15 — Correlation boxplots of RIP-G values for each iteration with genomic ancestry A, without ancestry conditioning. (TIFF) [file pgen.1004224.s015.tiff]

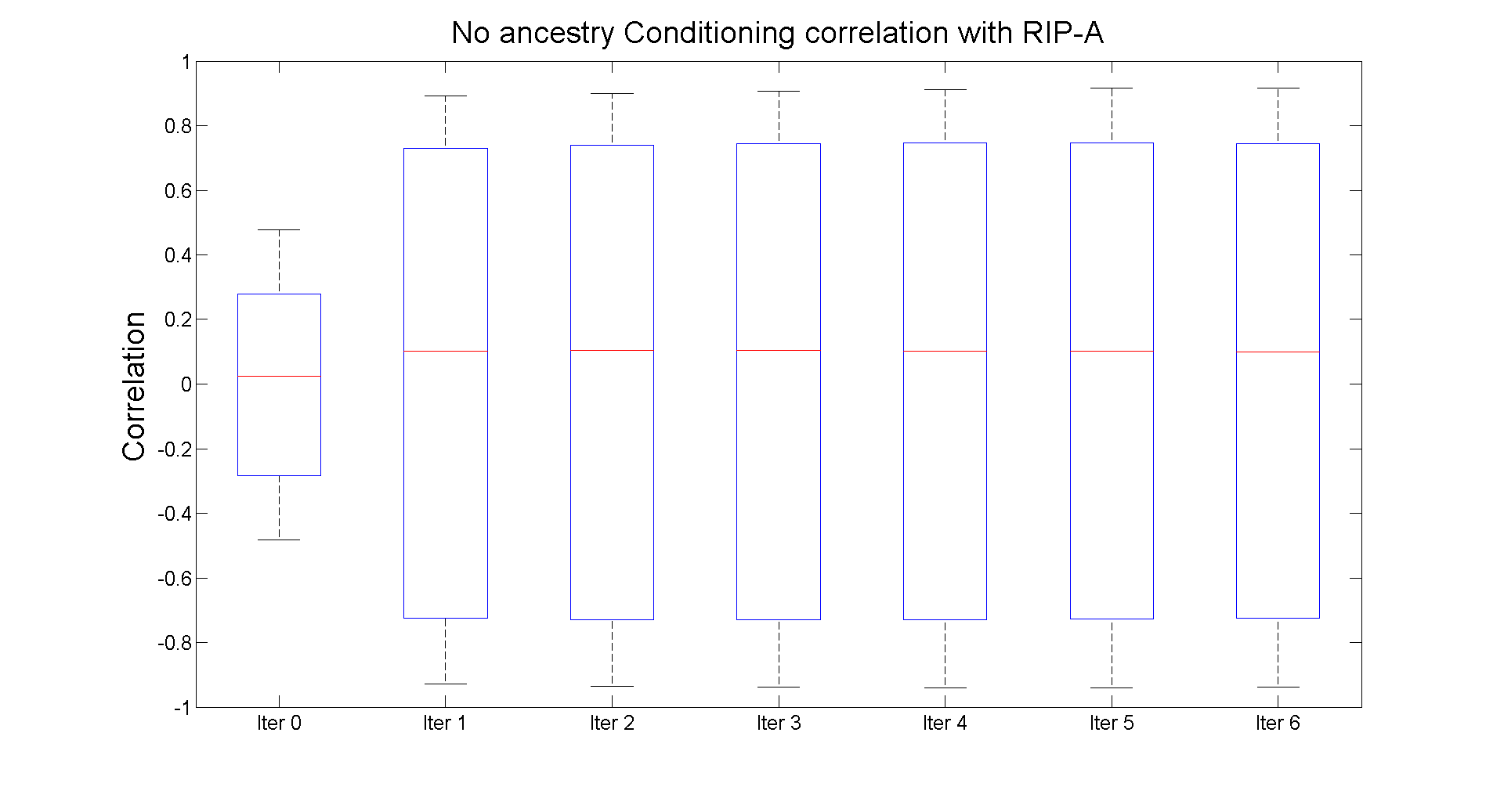

Supplement: Figure S16 — Correlation boxplots of RIP-G values for each iteration with RIP-A, without ancestry conditioning. (TIFF) [file pgen.1004224.s016.tiff]

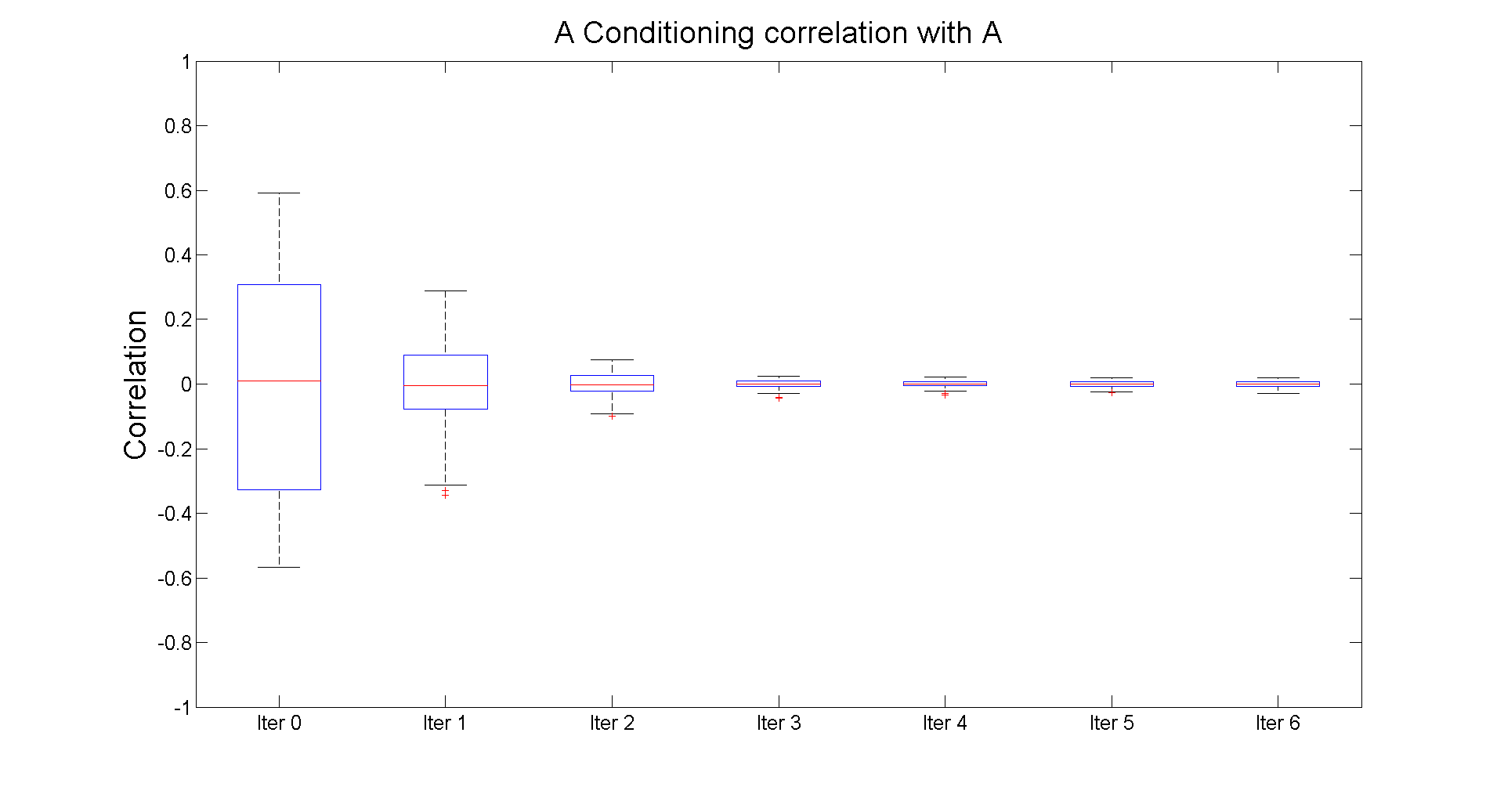

Supplement: Figure S17 — Correlation boxplots of RIP-G values for each iteration with A, conditioned on A. (TIFF) [file pgen.1004224.s017.tiff]

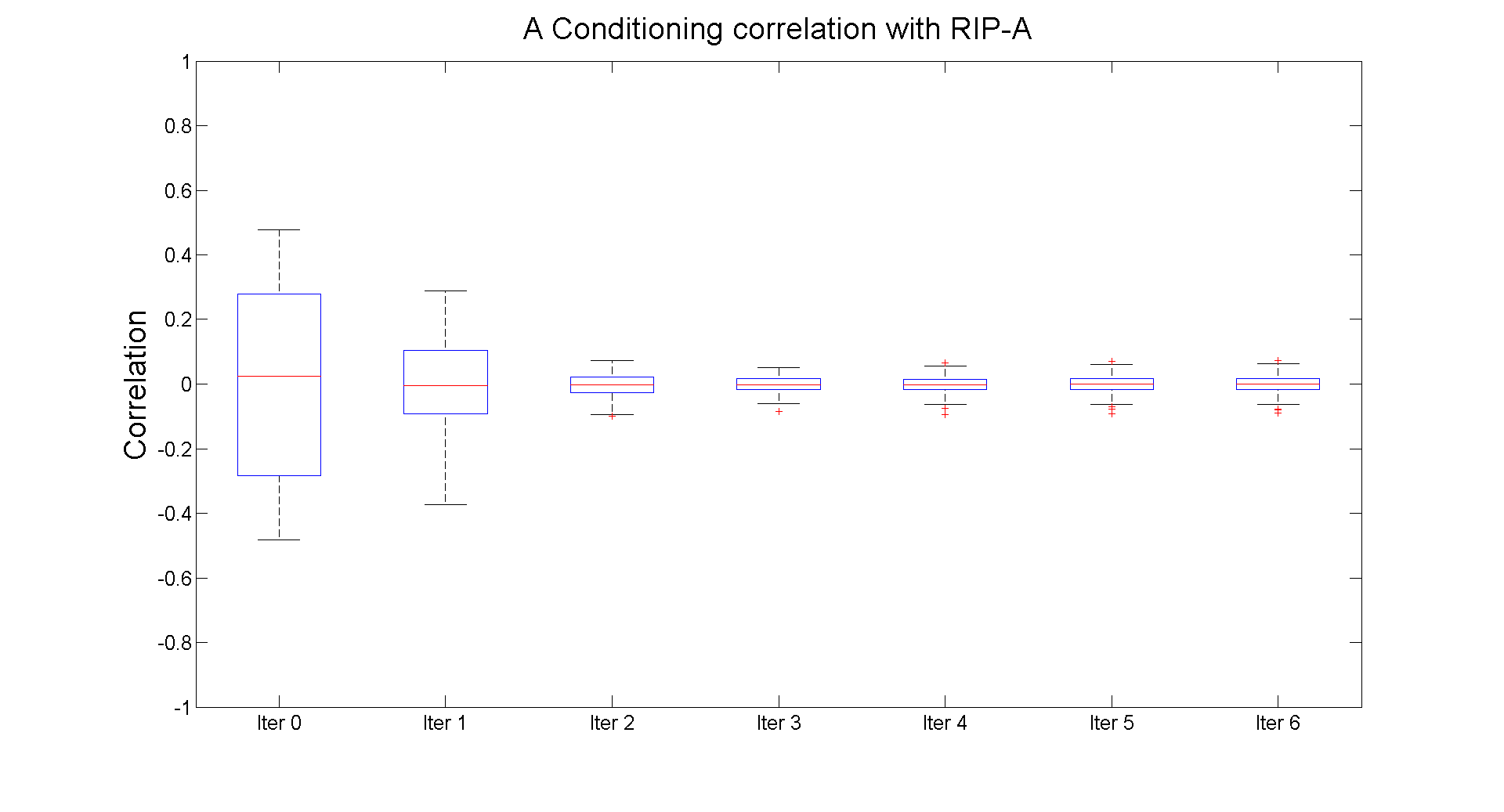

Supplement: Figure S18 — Correlation boxplots of RIP-G values for each iteration with RIP-A, conditioned on A. (TIFF) [file pgen.1004224.s018.tiff]

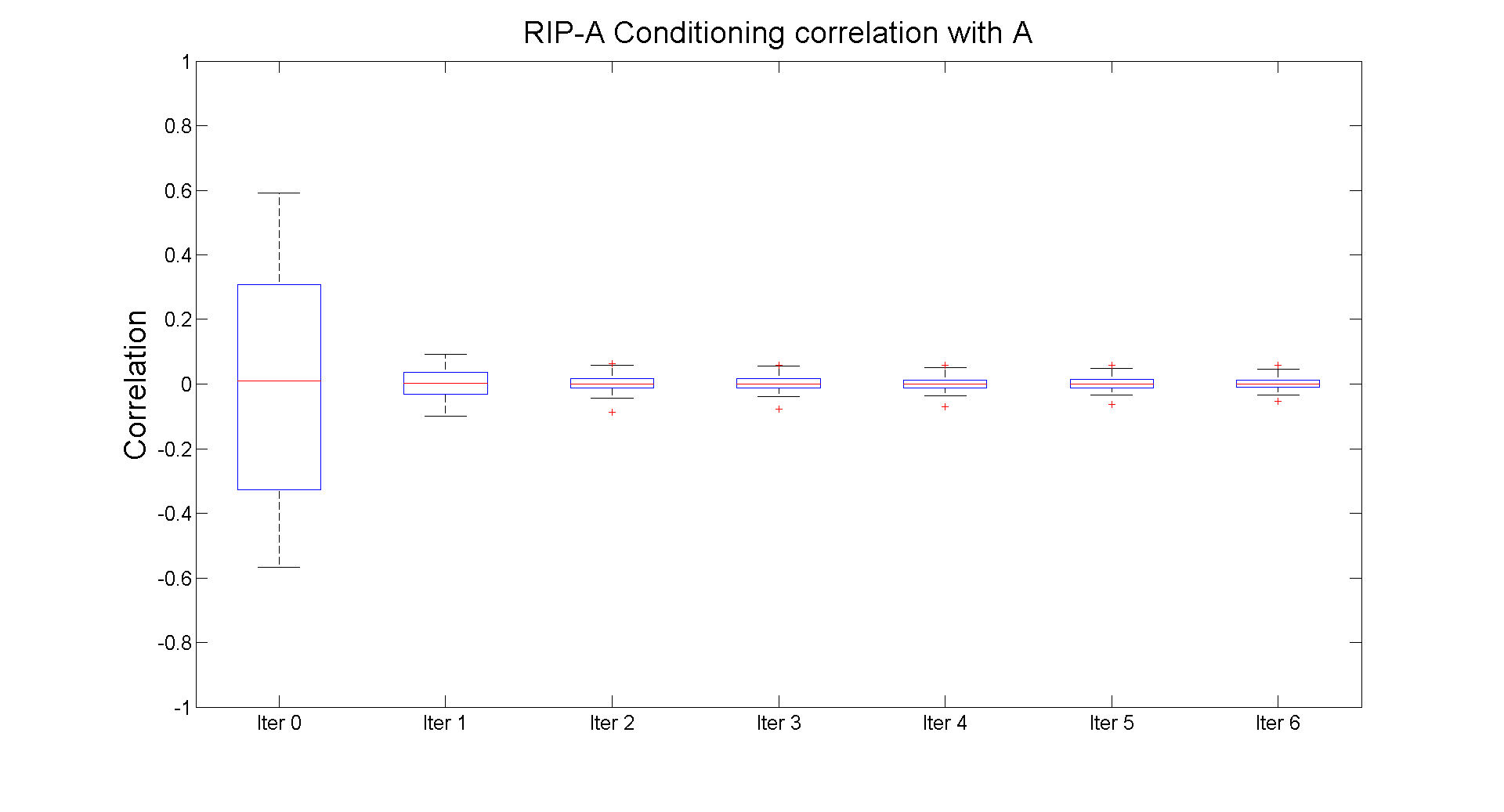

Supplement: Figure S19 — Correlation boxplots of RIP-G values for each iteration with A, conditioned on RIP-A. (TIFF) [file pgen.1004224.s019.tiff]

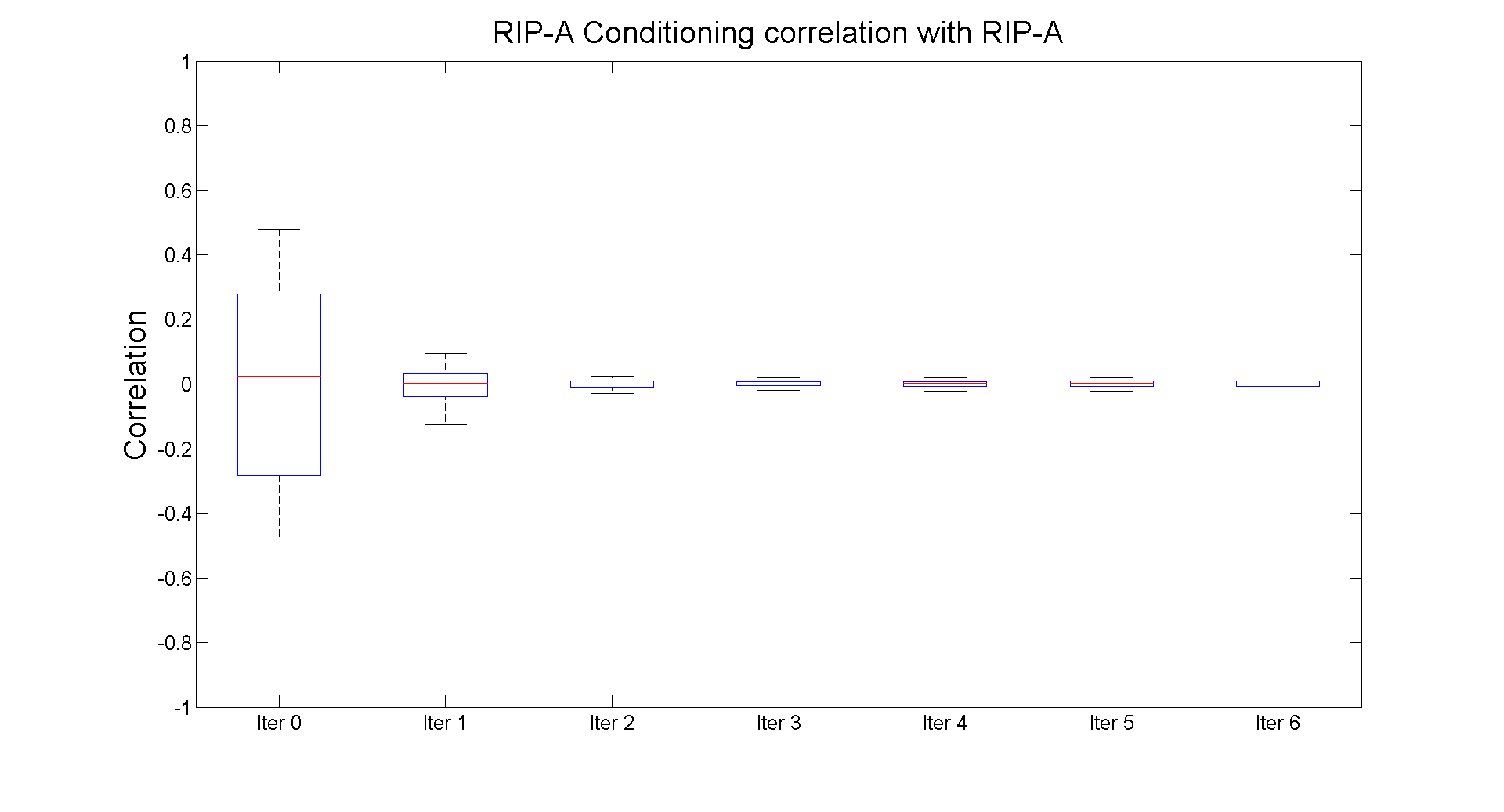

Supplement: Figure S20 — Correlation boxplots of RIP-G values for each iteration with RIP-A, conditioned on RIP-A. (TIFF) [file pgen.1004224.s020.tiff]

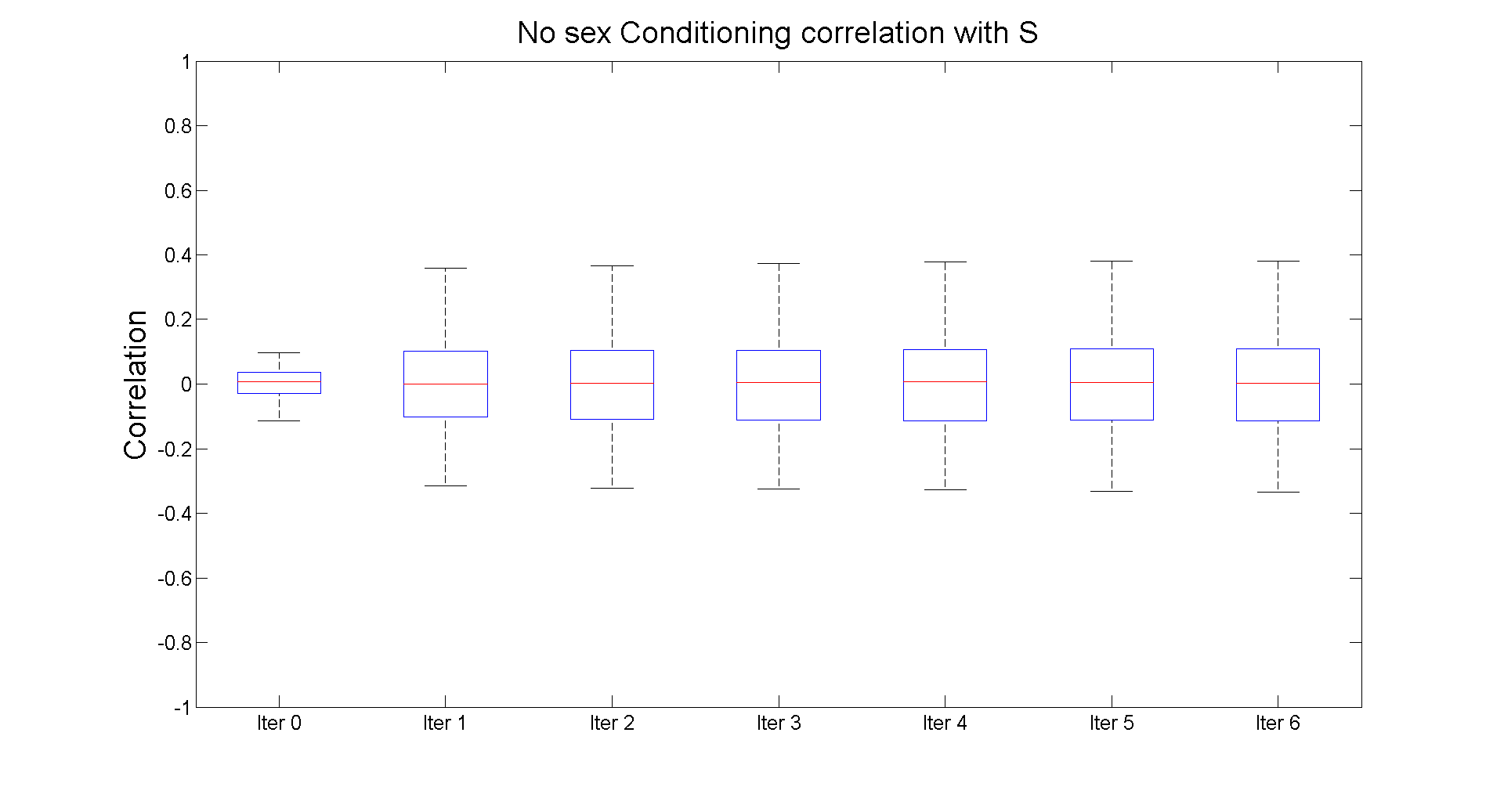

Supplement: Figure S21 — Correlation boxplots of RIP-G values for each iteration with S, without sex conditioning. (TIFF) [file pgen.1004224.s021.tiff]

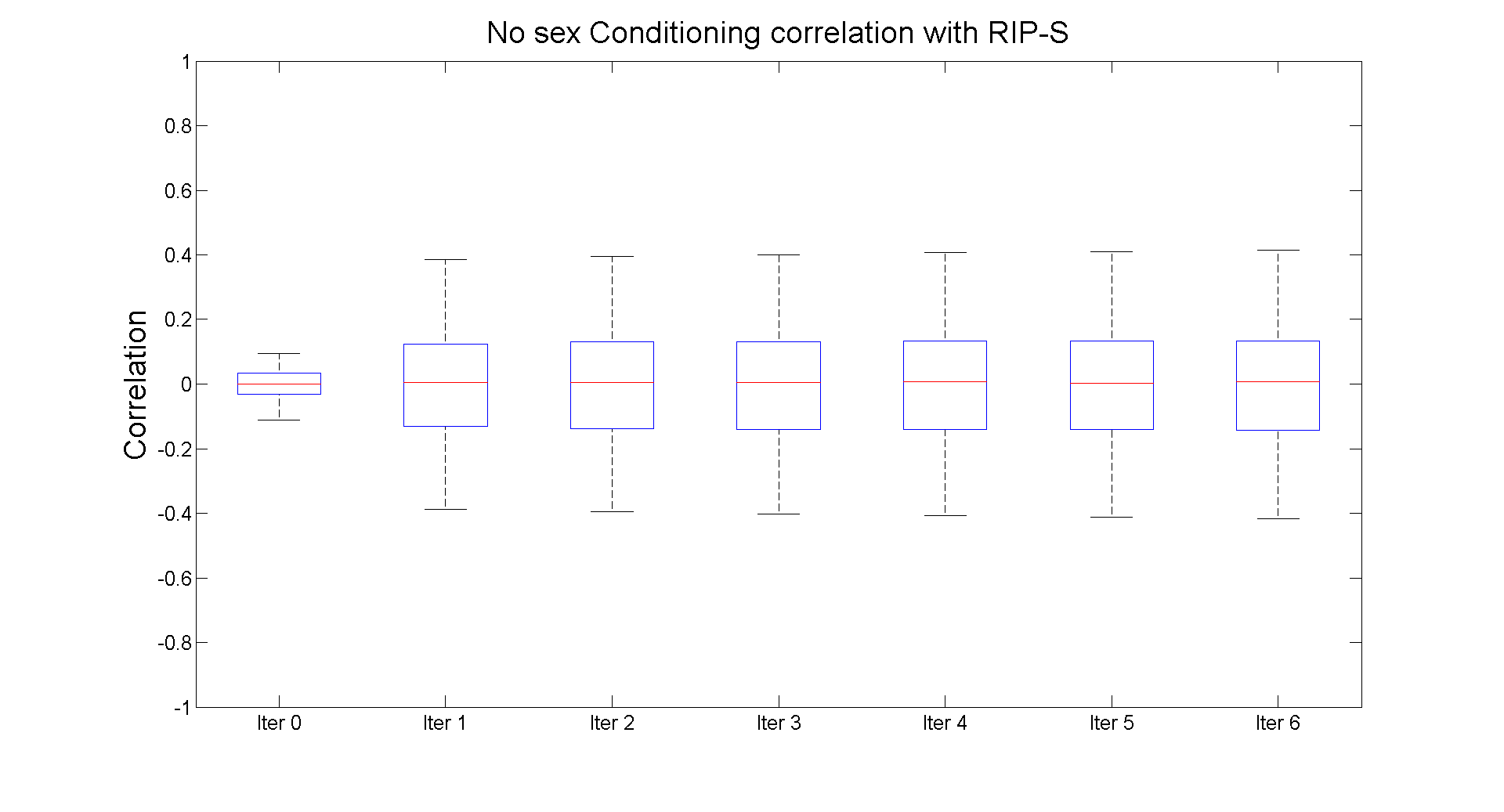

Supplement: Figure S22 — Correlation boxplots of RIP-G values for each iteration with RIP-S, without sex conditioning. (TIFF) [file pgen.1004224.s022.tiff]

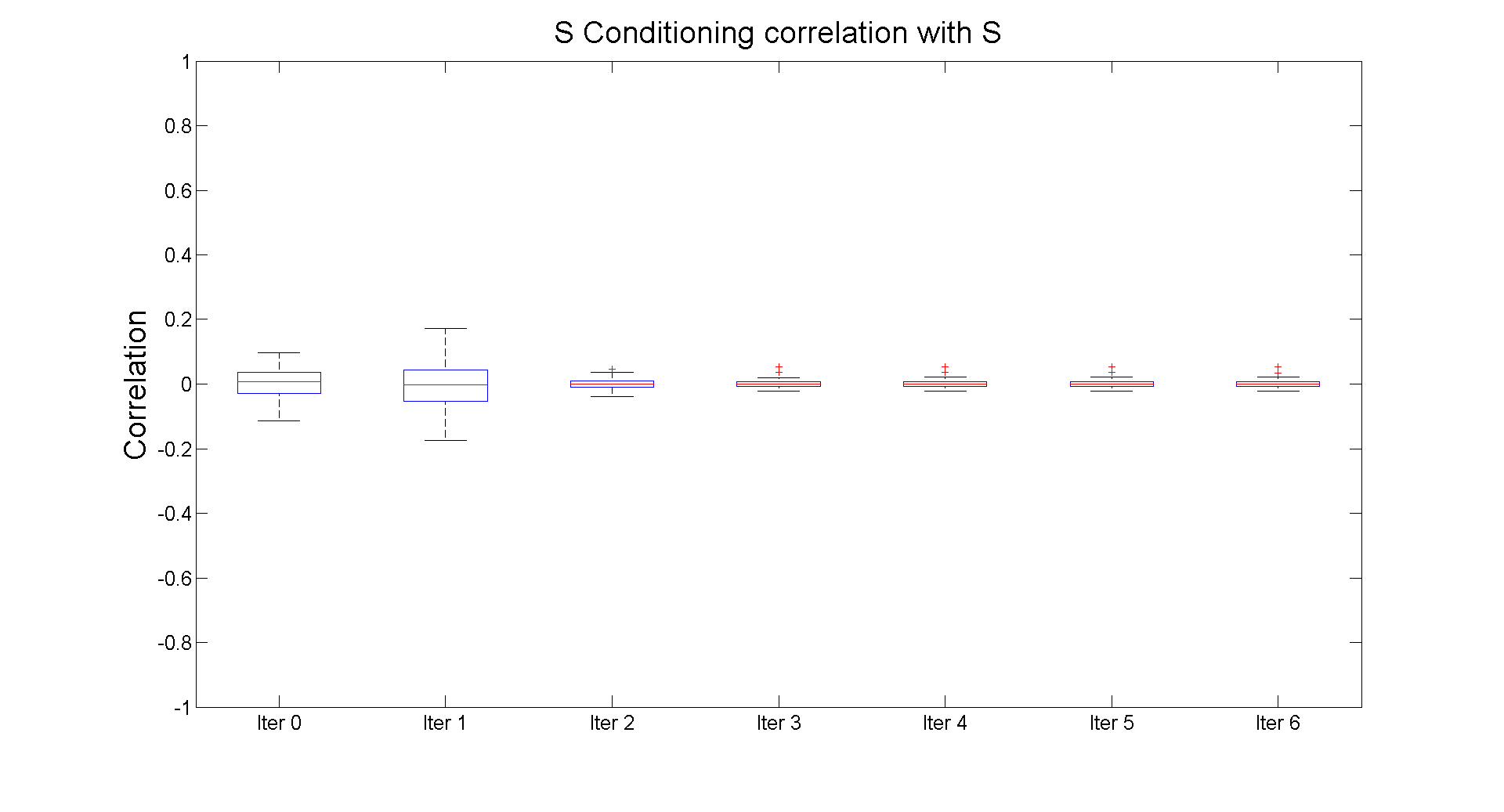

Supplement: Figure S23 — Correlation boxplots of RIP-G values for each iteration with S, conditioned on S. (TIFF) [file pgen.1004224.s023.tiff]

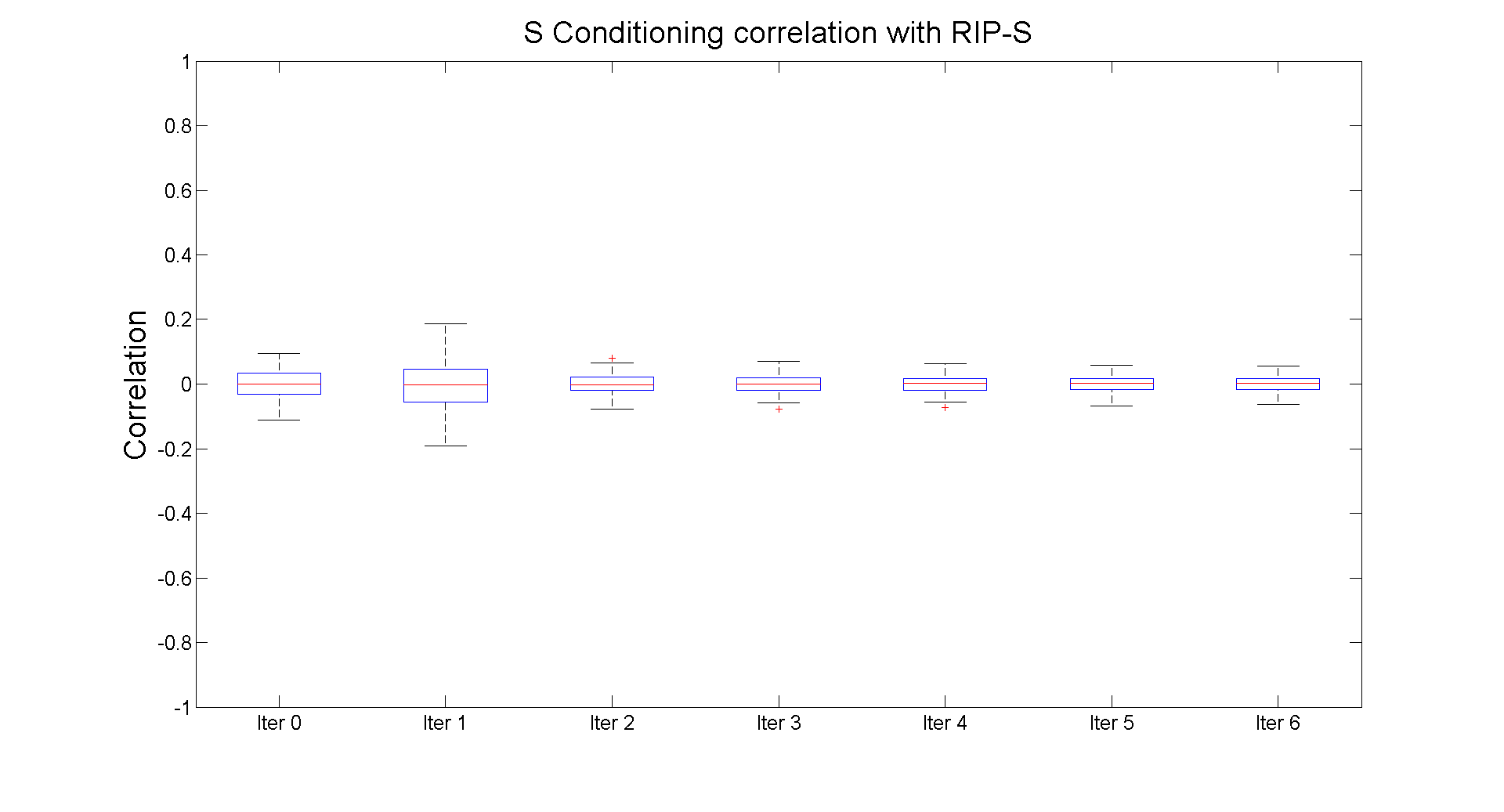

Supplement: Figure S24 — Correlation boxplots of RIP-G values for each iteration with RIP-S, conditioned on S. (TIFF) [file pgen.1004224.s024.tiff]

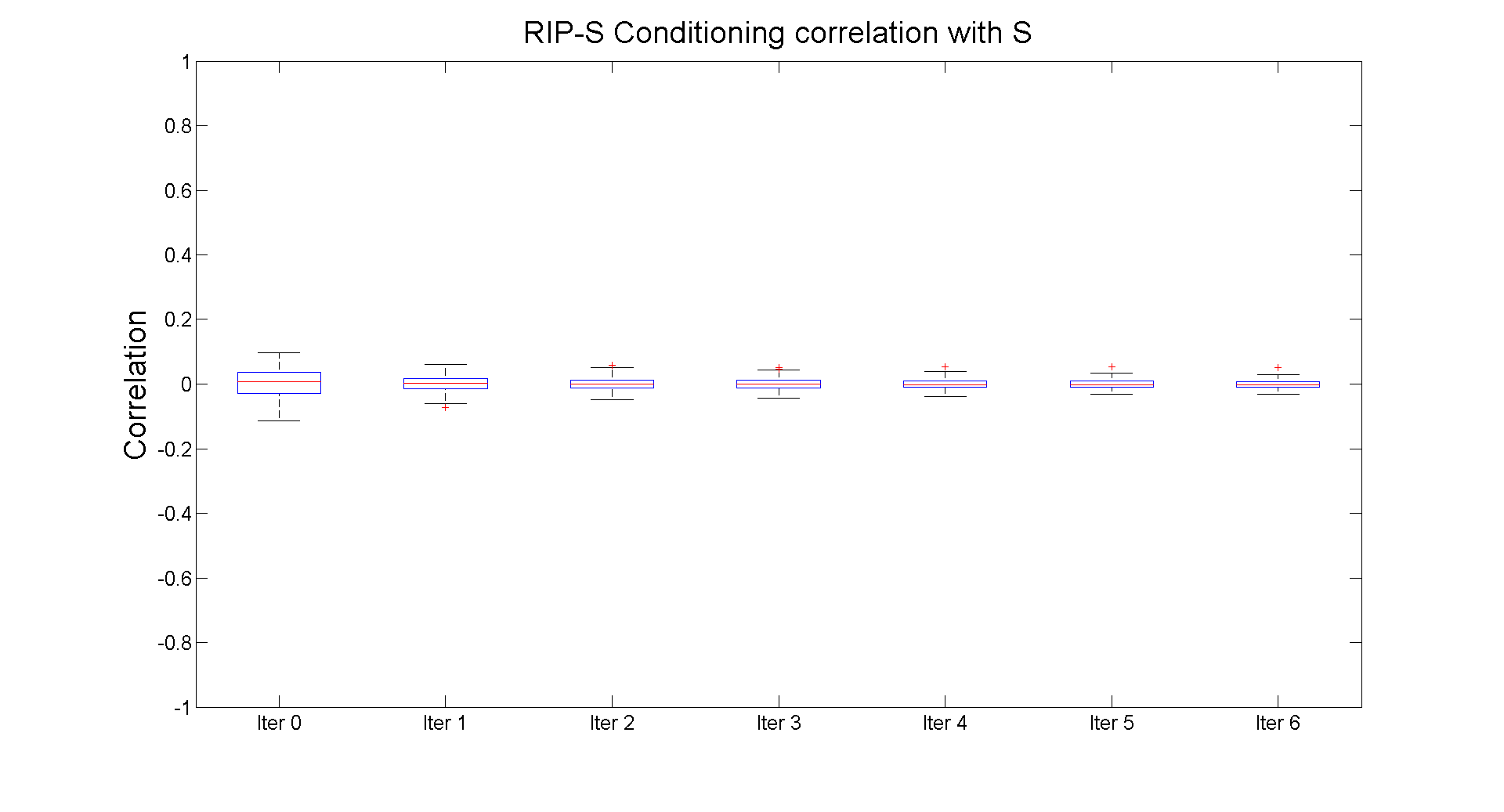

Supplement: Figure S25 — Correlation boxplots of RIP-G values for each iteration with A, conditioned on RIP-A. (TIFF) [file pgen.1004224.s025.tiff]

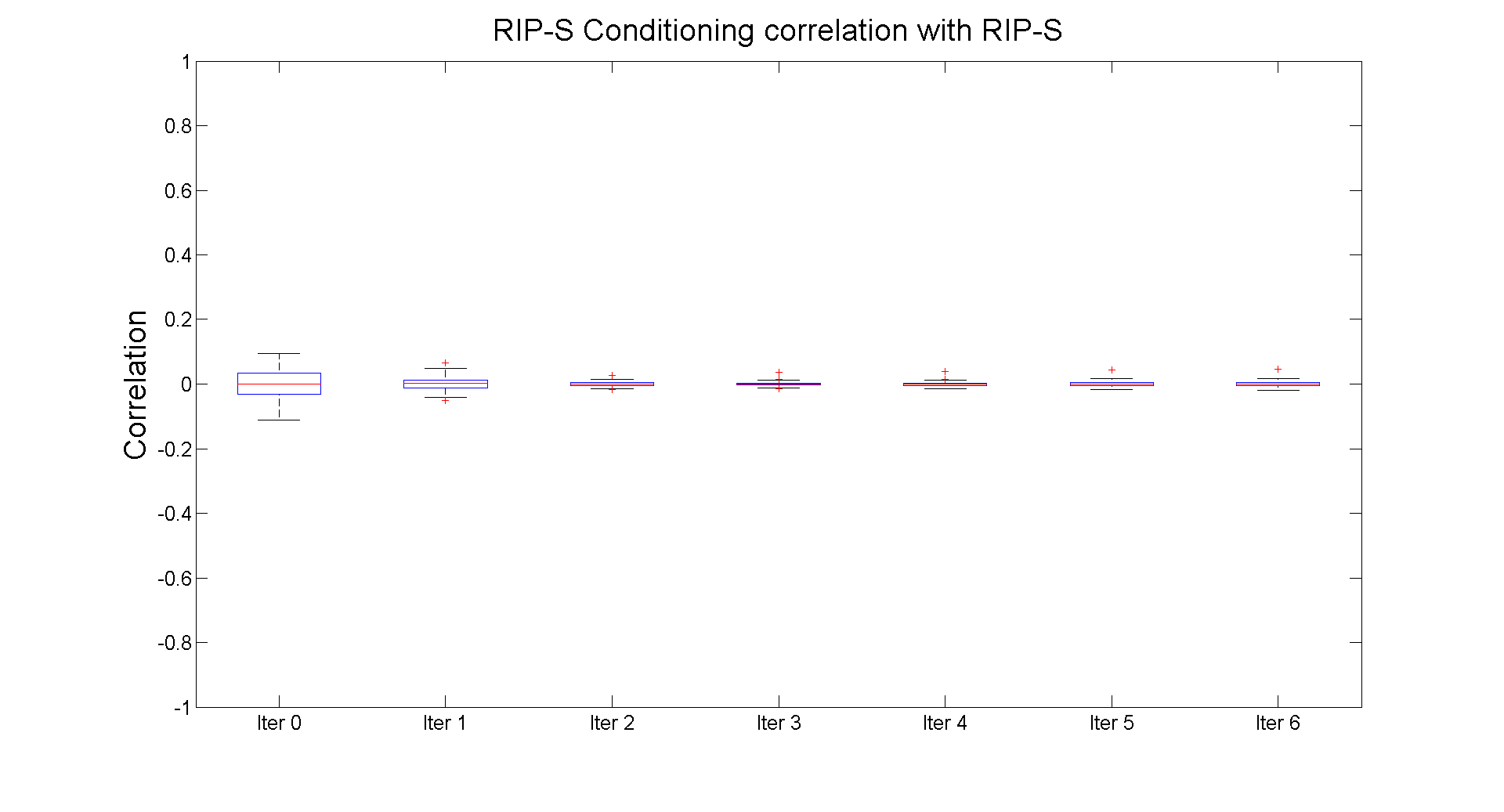

Supplement: Figure S26 — Correlation boxplots of RIP-G values for each iteration with RIP-A, conditioned on RIP-A. (TIFF) [file pgen.1004224.s026.tiff]

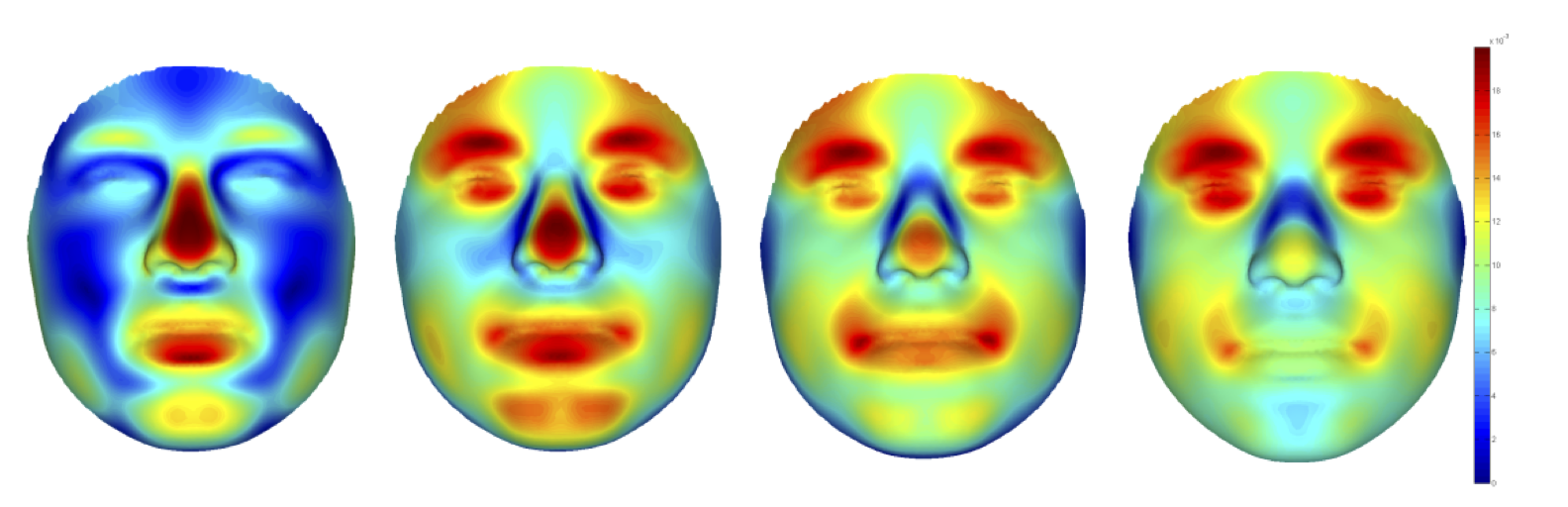

Supplement: Figure S27 — The effect of the rs13267109 SNP in FGFR1 using approach 1 (no conditioning for ancestry), left, approach 2 (conditioning for genomic ancestry), second from left, approach 3 (conditioning for RIP-A), second from right and approach 4 (conditioning for RIP-A using BRIM), right. (TIFF) [file pgen.1004224.s027.tiff]

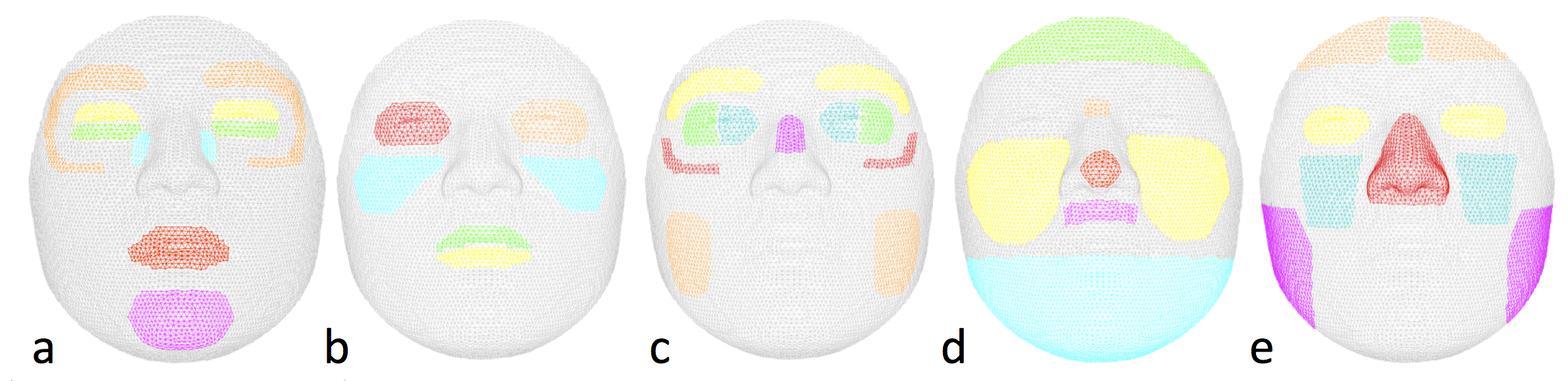

Supplement: Figure S28 — Facial Regions: (A) Orange, Orbital Ridges; Red, Lips; Yellow, Eyes Superior; Green. Eyes Iinferior; Blue, Paranasal Tissues; Pink, Chin. (B) Orange, Left Eye; Red, Right Eye; Yellow, Lower Lip; Green, Upper Lip; Blue Cheek Bones. (C) Orange, Cheeks; Pink, Nose ridge; Yellow, Browridges; Green, Lateral half of eyes; Blue; Medial half of eyes; Red, Cheekbones. (D) Green, Forehead; Yellow, Midface; Red, Nasal tip; Pink, Philtrum; Orange, Nasal bridge; Blue, Lower face. (E) Green, Metopic ridge; Orange, Forehead sides; Yellow, Eyes; Red, Nose; Blue, Malars; Pink, Lateral midface. (TIFF) [file pgen.1004224.s028.tiff]

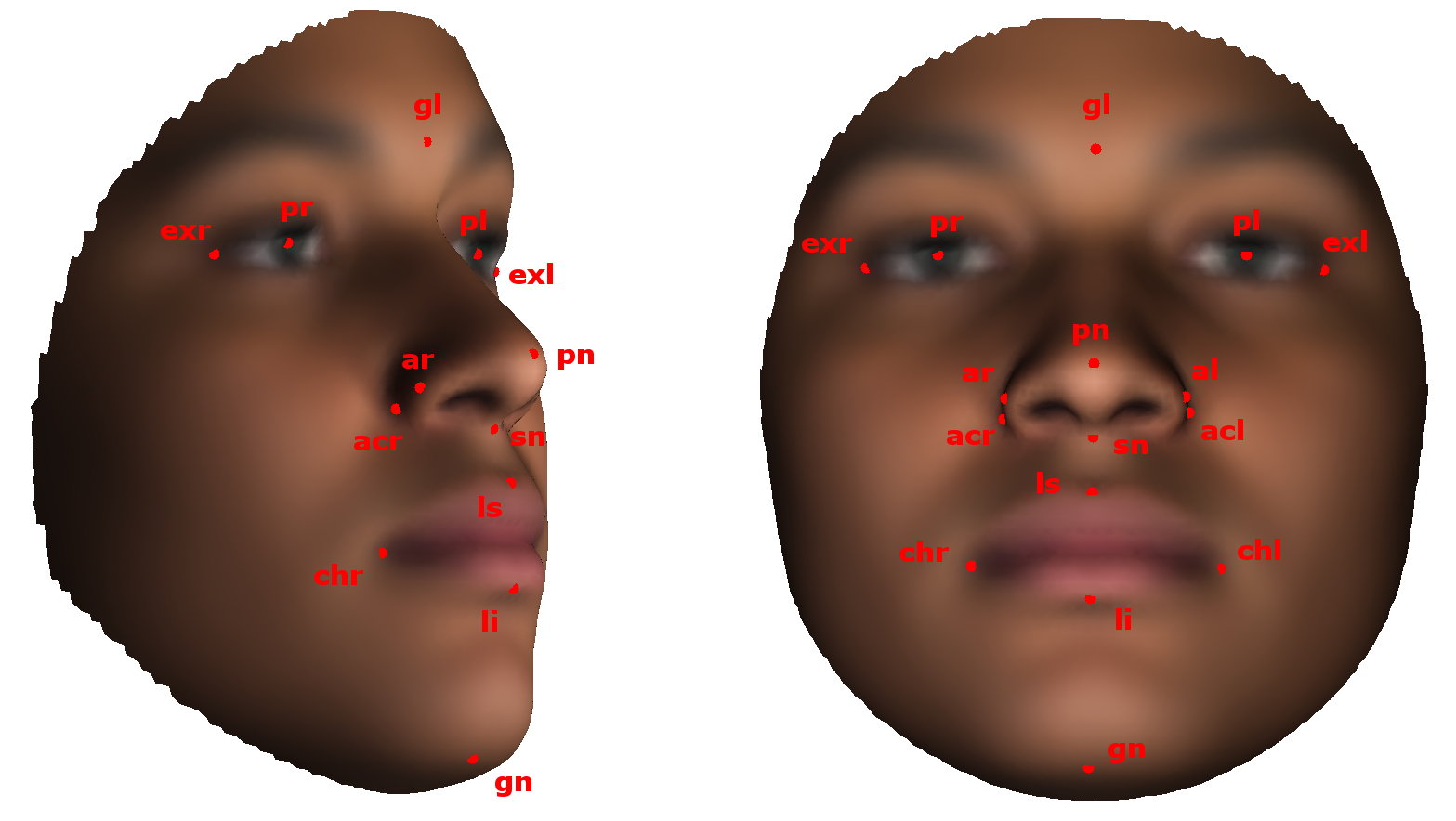

Supplement: Figure S29 — Manually annotated landmarks: gl = Glabella; pn = Pronasale; sn = Subnasale; ls = Labiale Superiusinferius; li = Labiale Iinferius; gn = Gnathion; exr = Right Eendocanthion; pr = Right Ppupil; pl = Left Ppupil; enl = Left Eendocanthion; ar = Right Alar; chr = Chelion right; chl = Chelion left. (TIFF) [file pgen.1004224.s029.tiff]

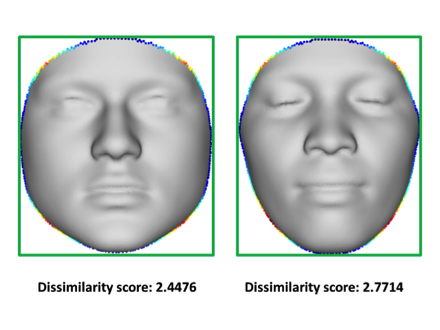

Supplement: Figure S30 — Measurement of facial squareness: The facial border is projected onto the XY plane and compared to a fitted square. The result is a dissimilarity score with a higher/lower score indicating a less/more square face. The colors in the border points indicate their distance to the square. (TIFF) [file pgen.1004224.s030.tiff]

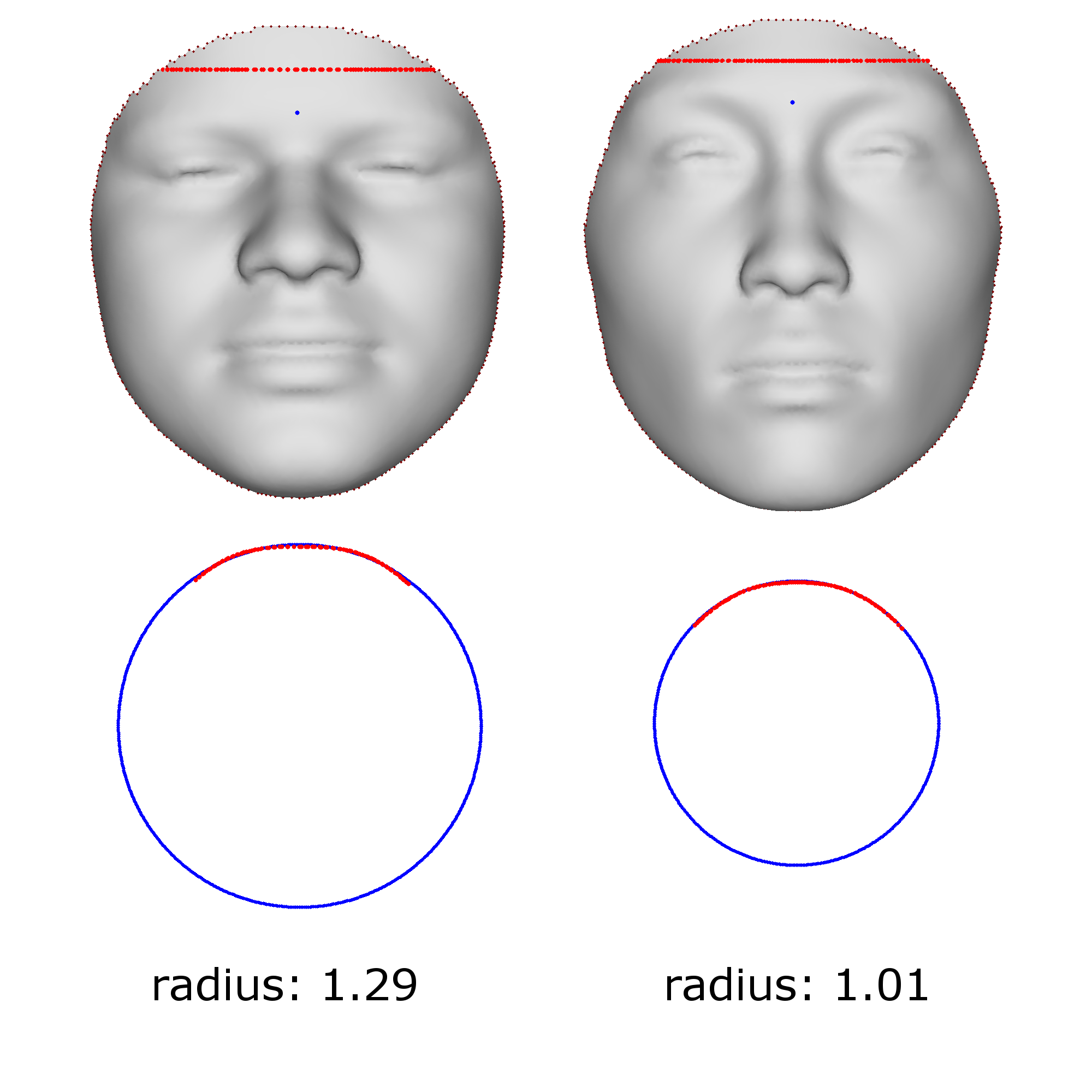

Supplement: Figure S31 — A proxy for head circumference: The forehead intersection with a plane halfway the Glabella and the top of the forehead is determined. This generates and arc segment through which a circle is fitted. The radius of the fitted circle serves as a proxy for head circumference. (TIFF) [file pgen.1004224.s031.tiff]

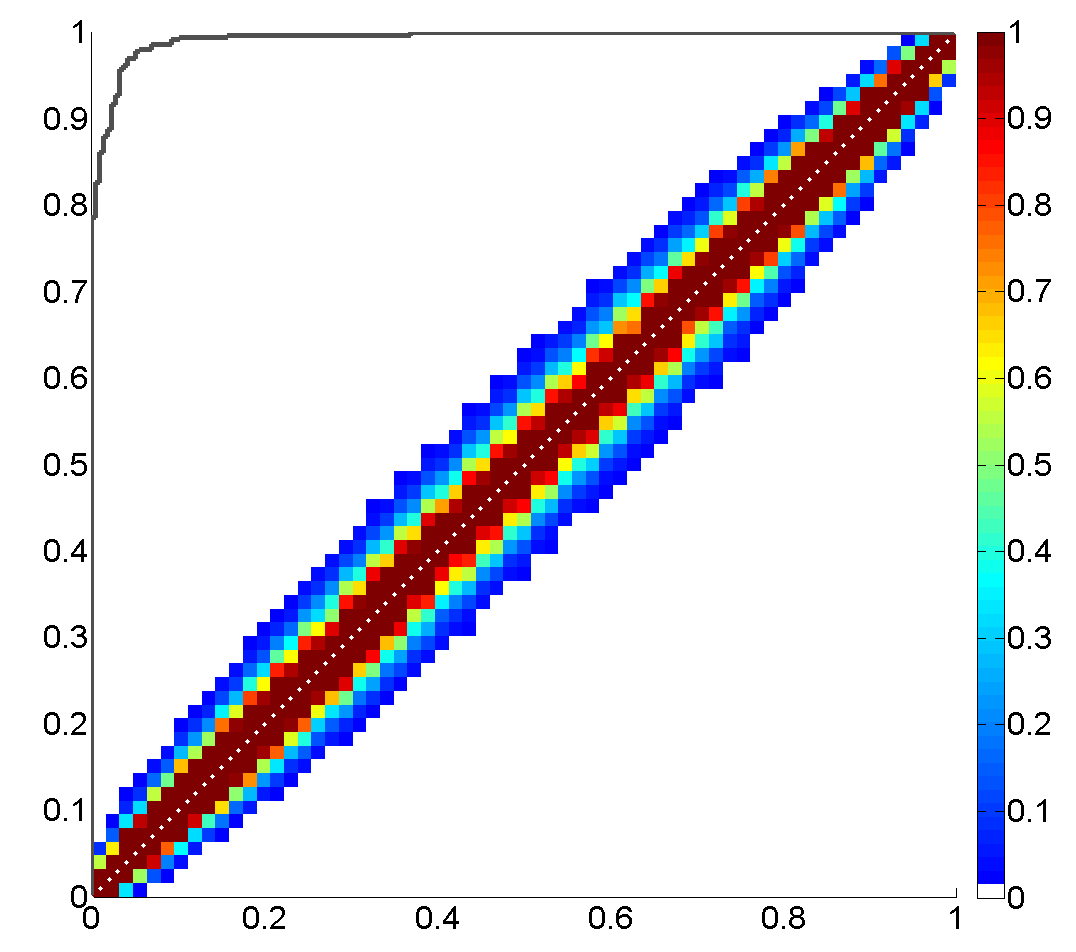

Supplement: Figure S32 — Receiver operator curve (ROC) showing the ability of facial femininity (RIP-S) to correctly classify faces by self-reported sex. (TIFF) [file pgen.1004224.s032.tiff]

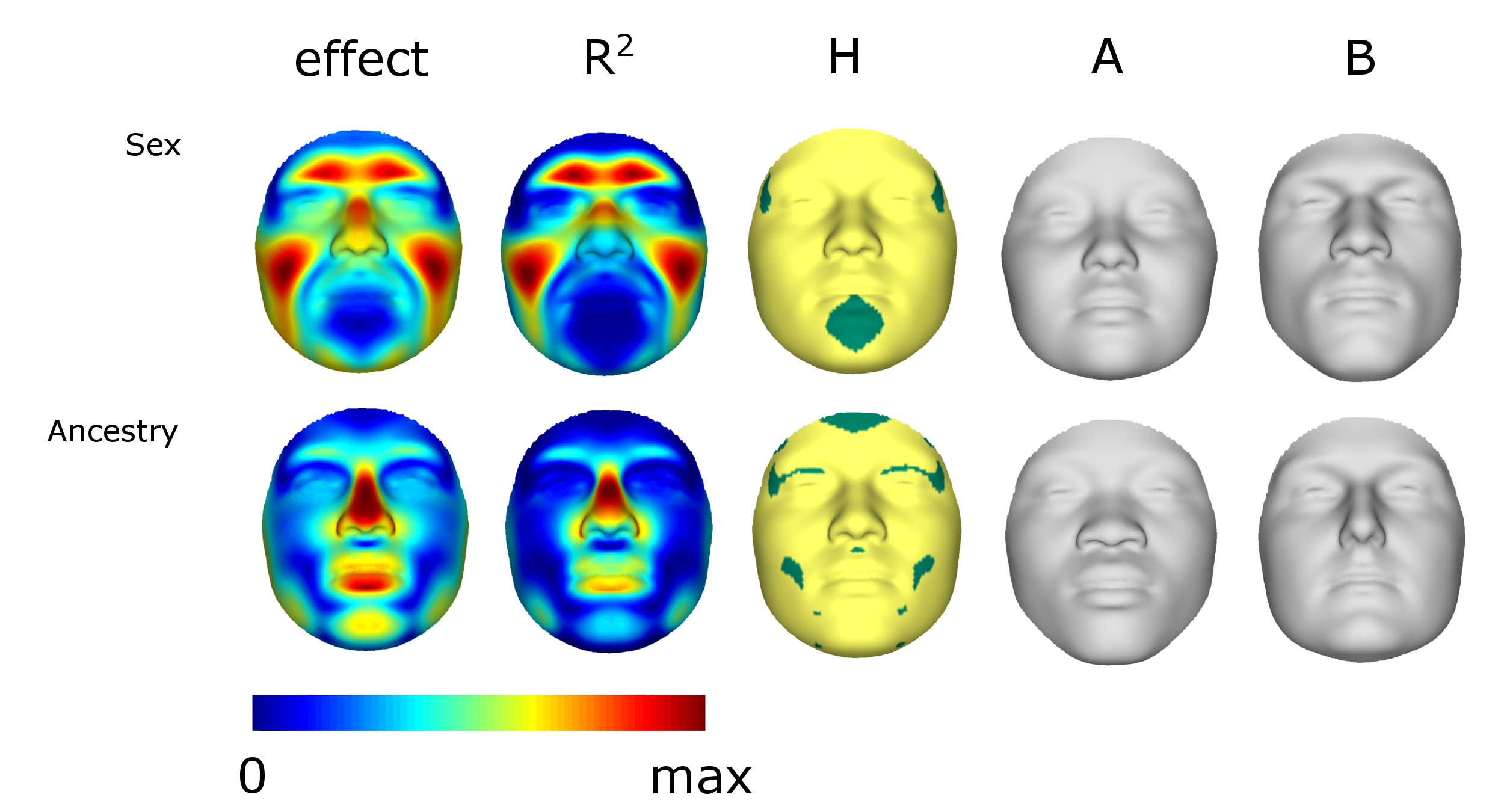

Supplement: Figure S33 — The effect, effect-size (r2), significance of the effect (H), and two shape transformations at opposite sides (+3 and −3 times the standard deviation) of the RIP distribution for sex (top row) and ancestry (bottom row). The maximum values for r2 can be found in Table S3. (TIFF) [file pgen.1004224.s033.tiff]

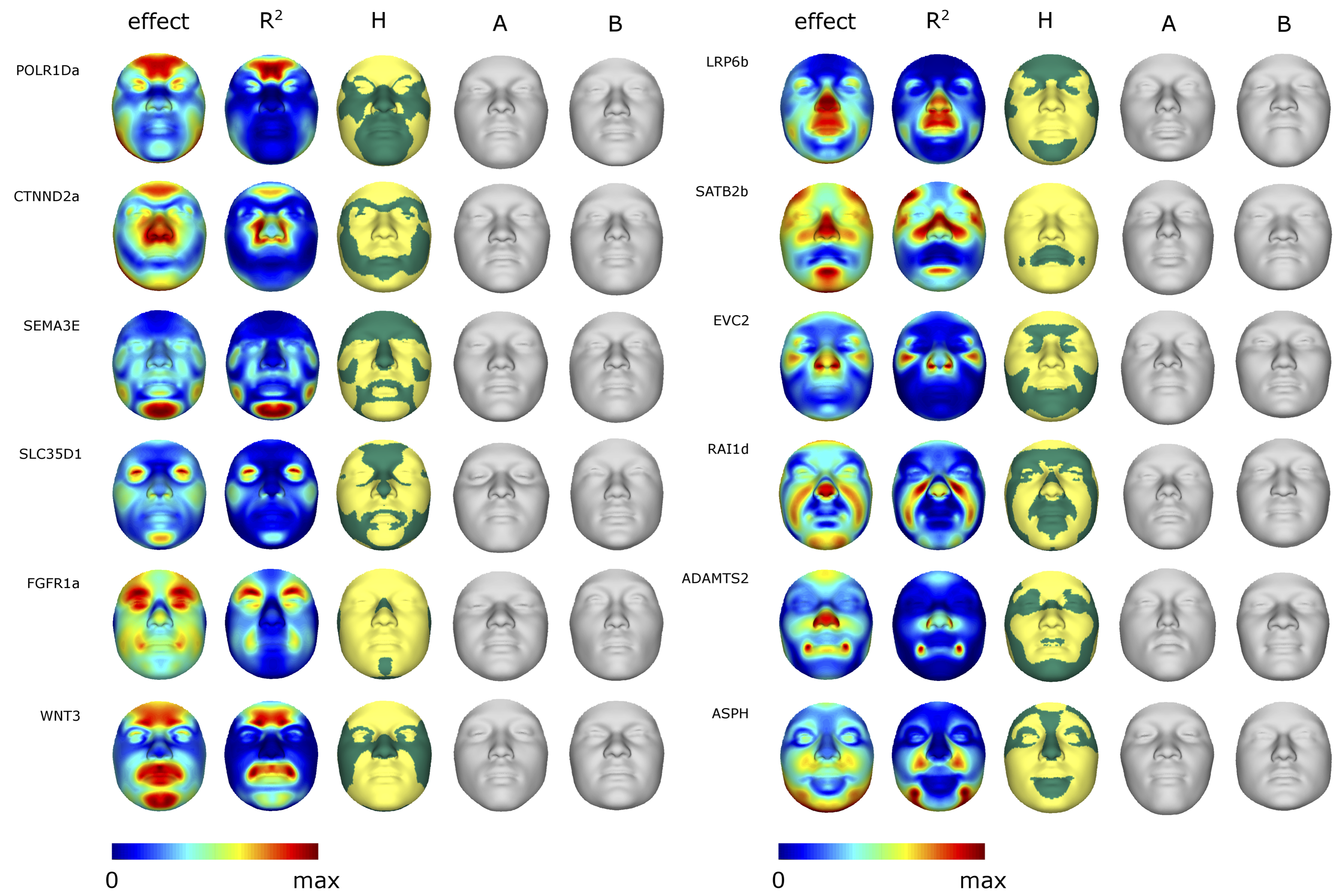

Supplement: Figure S34 — The effect, effect-size (r2), significance of the effect (H), and two shape transformations at opposite sides (+X and −X times the standard deviation) of the RIP distribution. The maximum values for R2 can be found in Table S3. PART 1. (TIFF) [file pgen.1004224.s034.tiff]

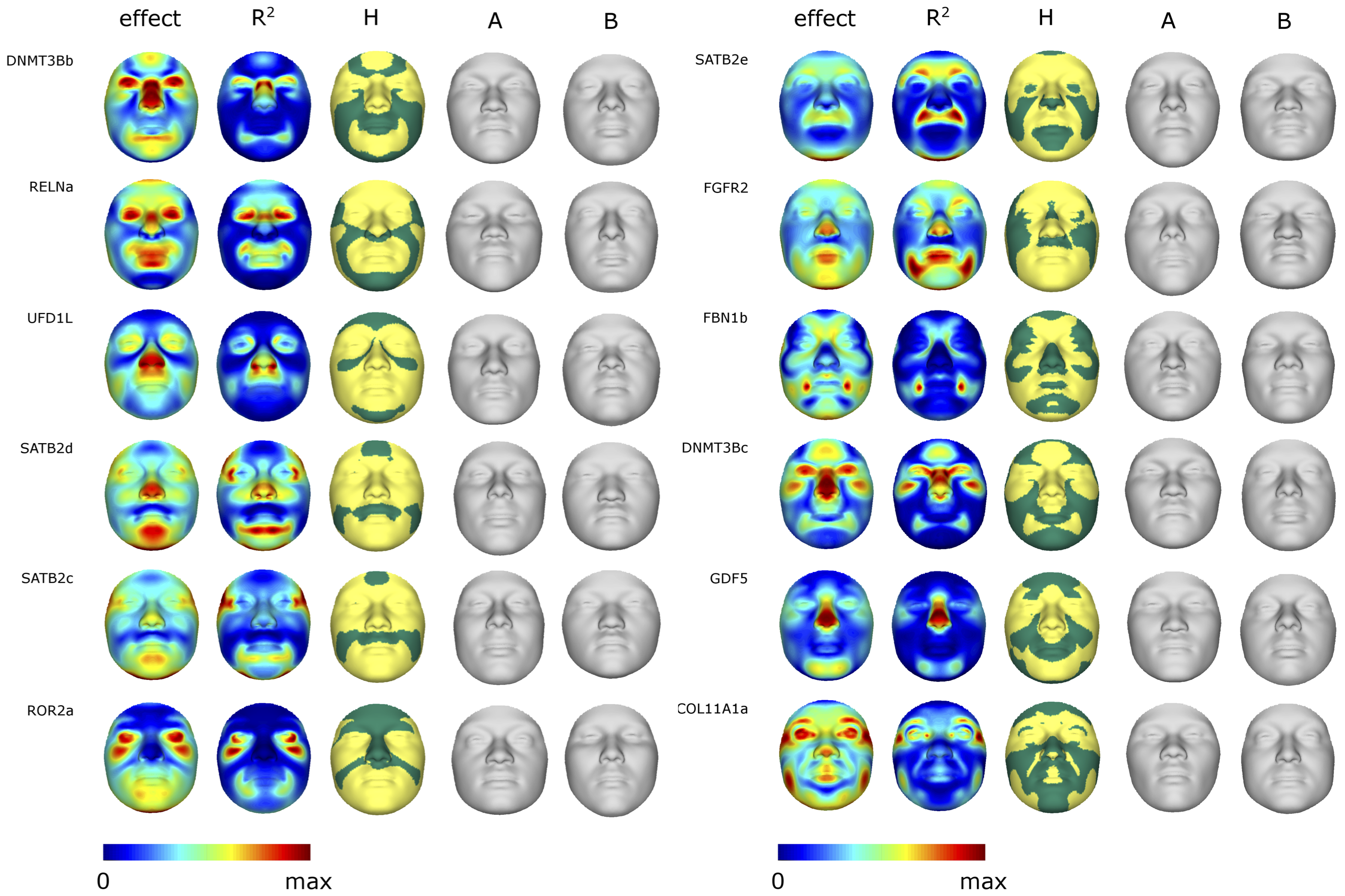

Supplement: Figure S35 — The effect, effect-size (r2), significance of the effect (H), and two shape transformations at opposite sides (+X and −X times the standard deviation) of the RIP distribution. The maximum values for R2 can be found in Table S3. PART 2. (TIFF) [file pgen.1004224.s035.tiff]

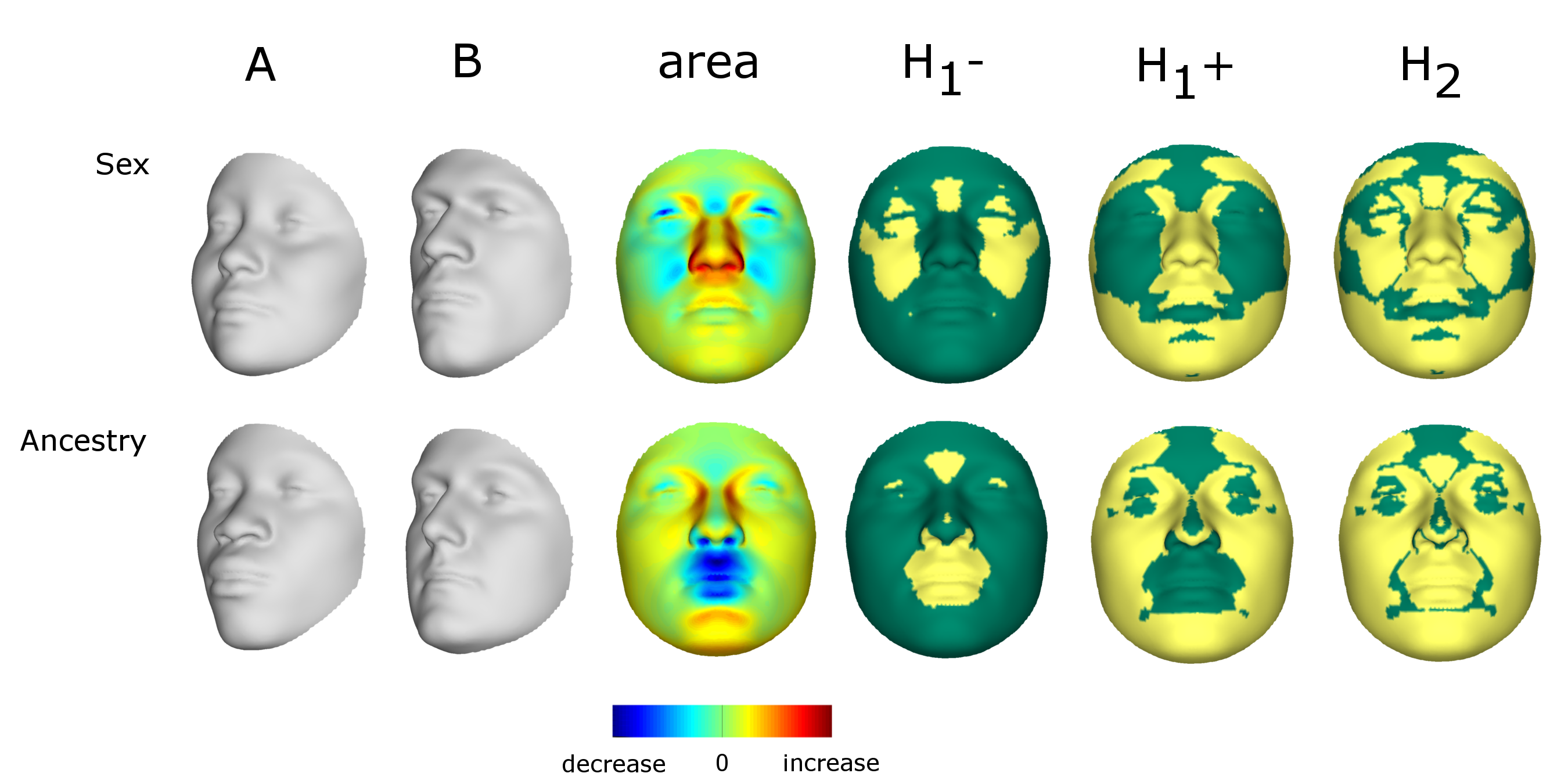

Supplement: Figure S36 — Facial area changes due to sex and ancestry. (TIFF) [file pgen.1004224.s036.tiff]

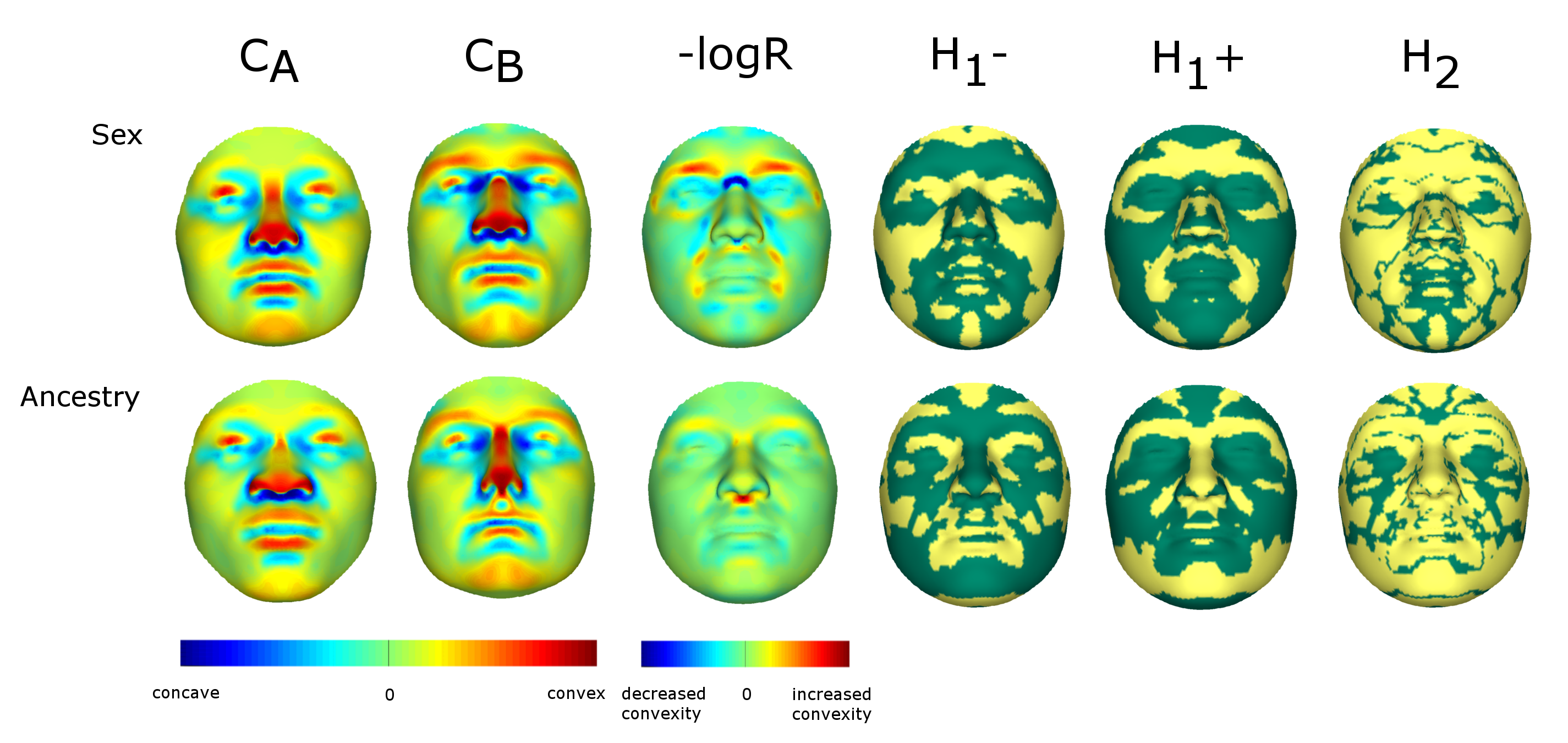

Supplement: Figure S37 — Facial curvature changes due to sex and ancestry. (TIFF) [file pgen.1004224.s037.tiff]

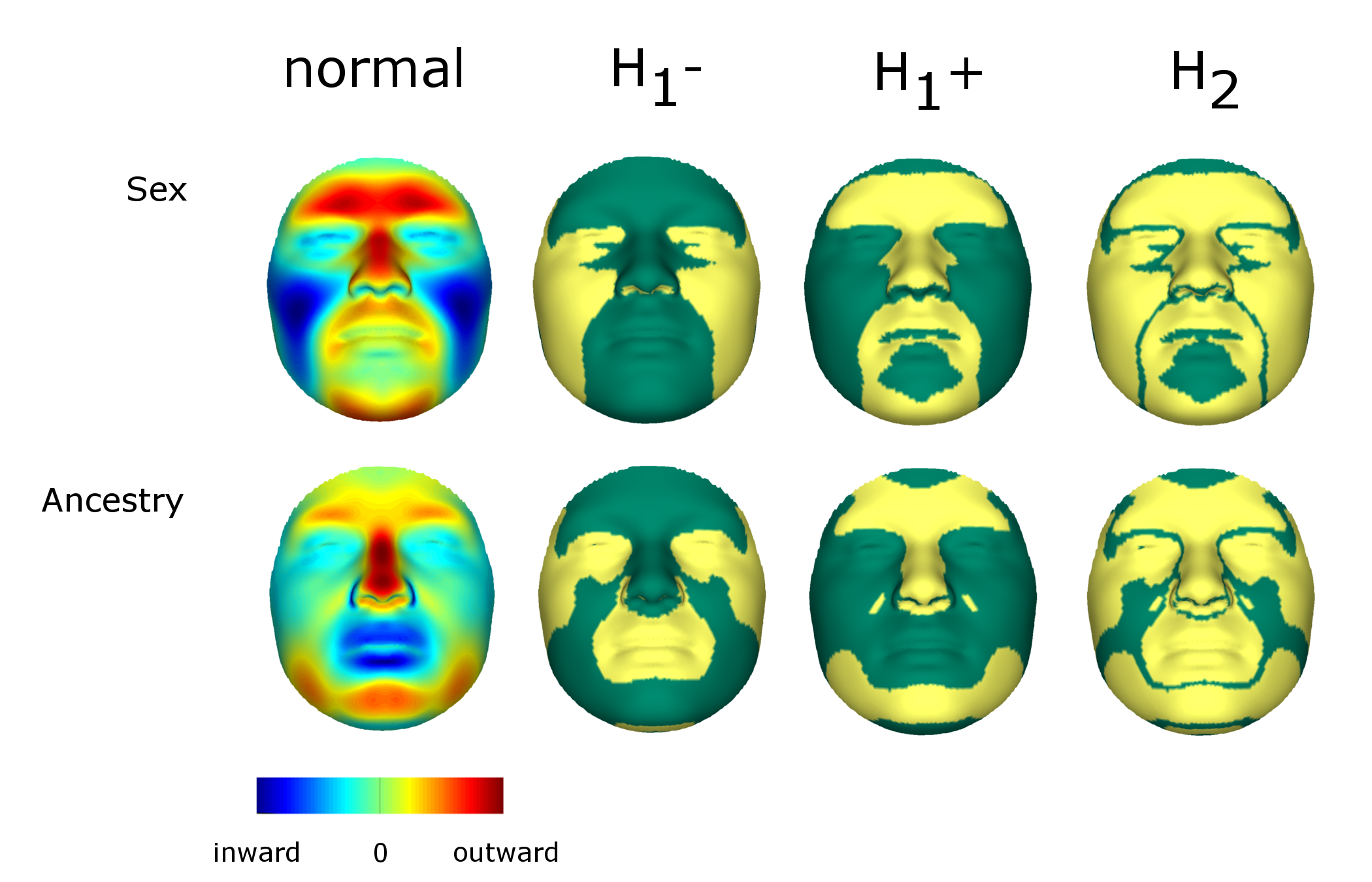

Supplement: Figure S38 — Normal displacements due to sex and ancestry. (TIFF) [file pgen.1004224.s038.tiff]

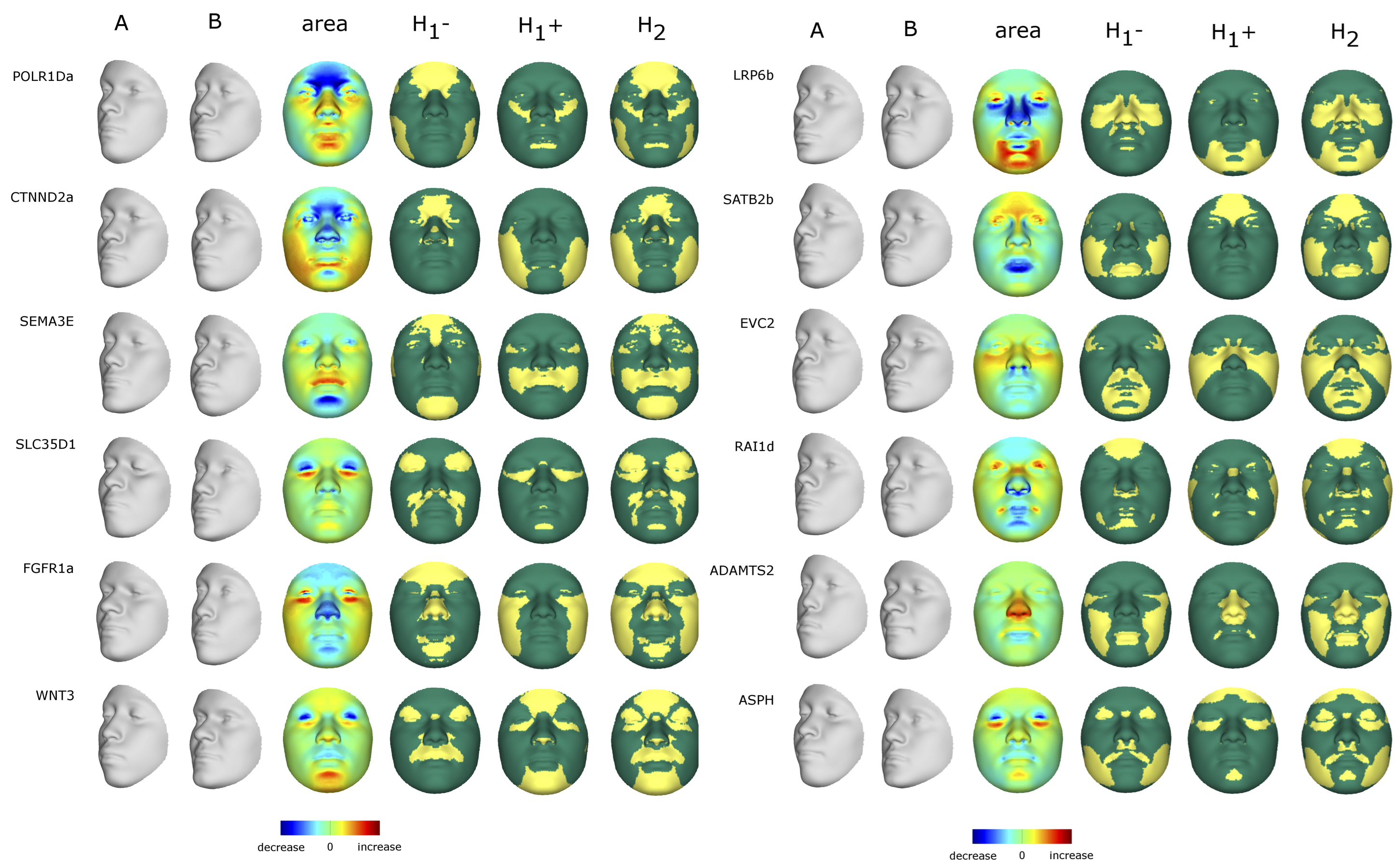

Supplement: Figure S39 — Facial area changes due to candidate genes. PART 1. (TIFF) [file pgen.1004224.s039.tiff]

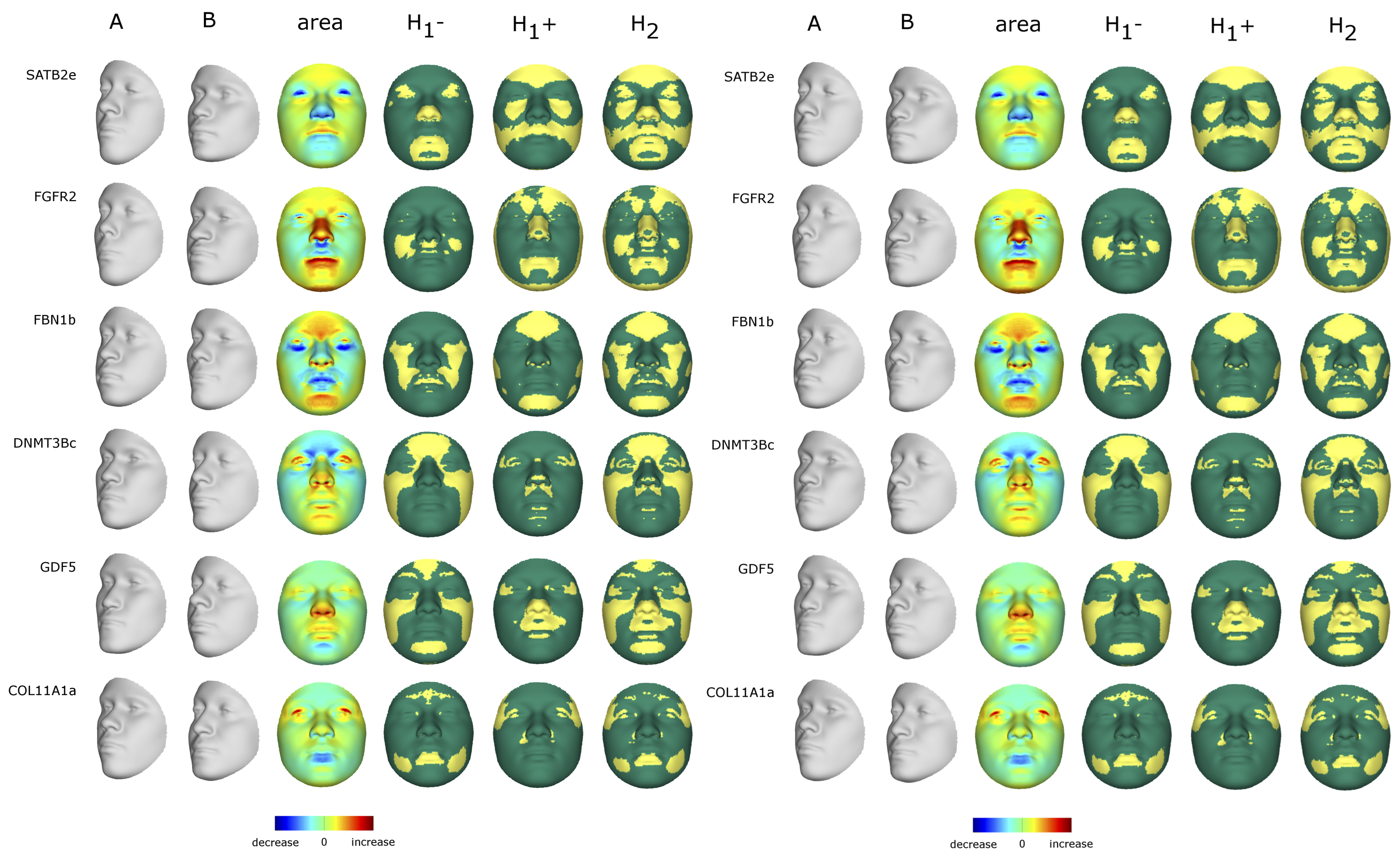

Supplement: Figure S40 — Facial area changes due to candidate genes. PART 2. (TIFF) [file pgen.1004224.s040.tiff]

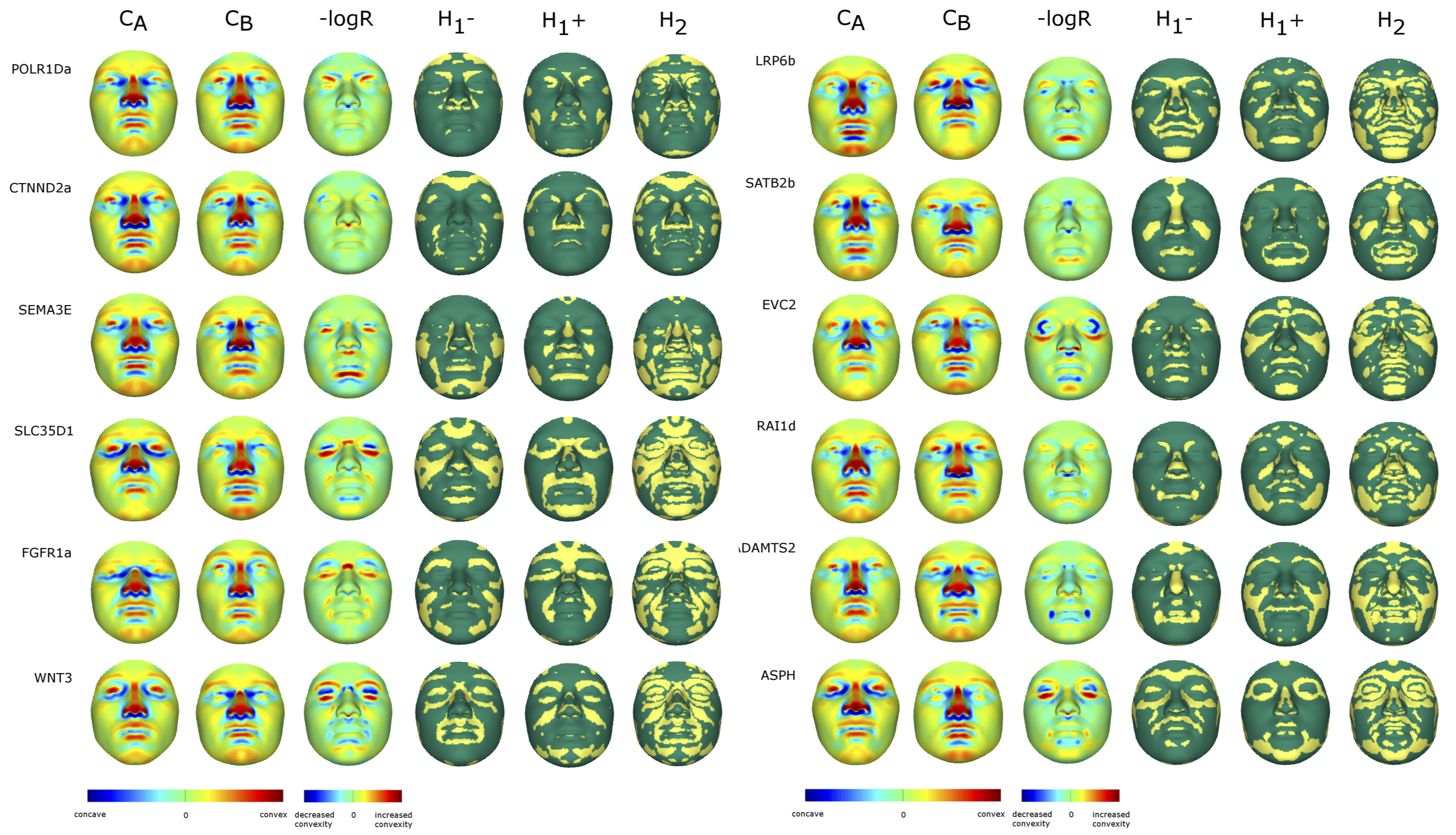

Supplement: Figure S41 — Facial curvature changes due to candidate genes. PART 1. (TIFF) [file pgen.1004224.s041.tiff]

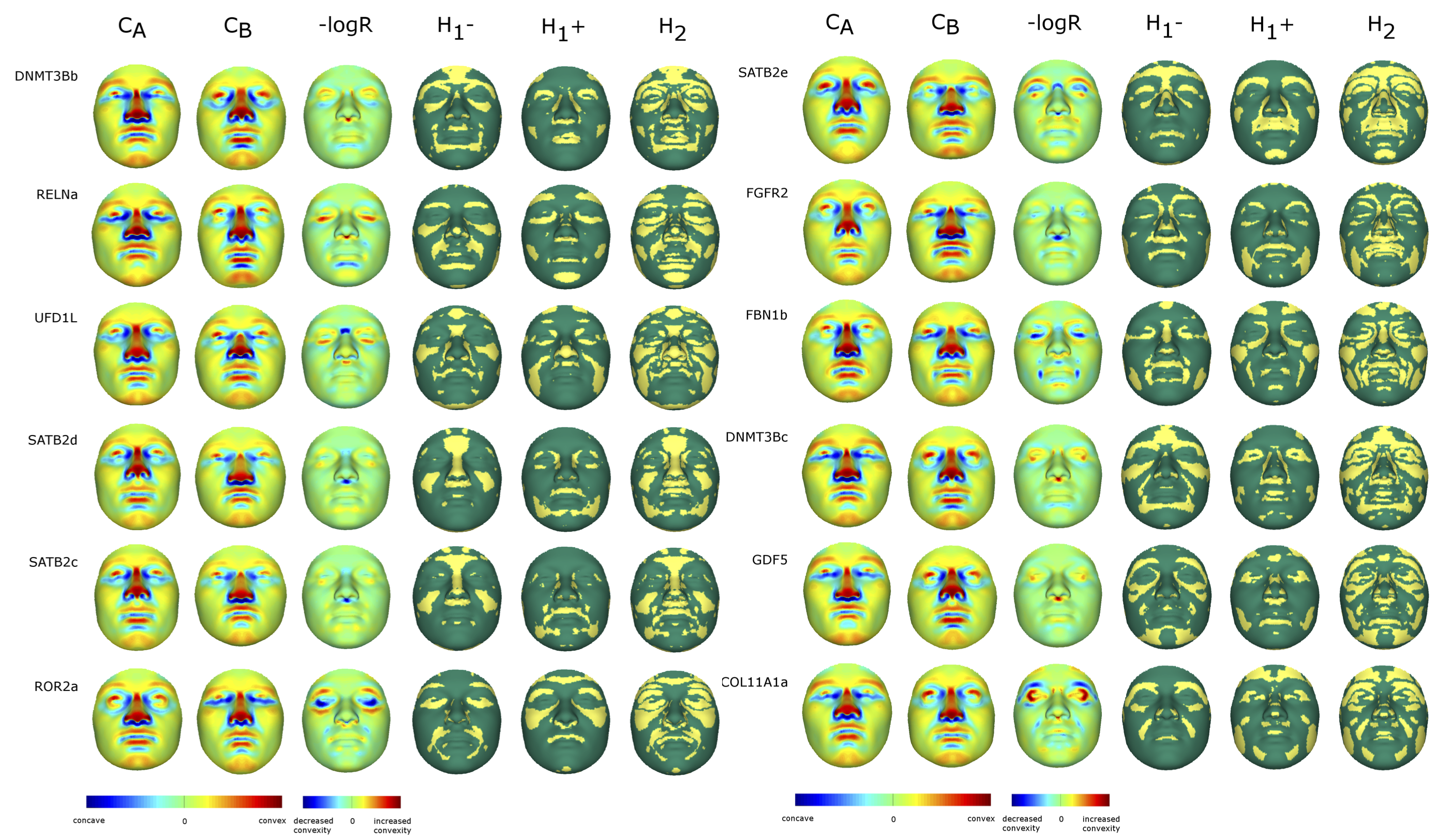

Supplement: Figure S42 — Facial curvature changes due to candidate genes. PART 2. (TIFF) [file pgen.1004224.s042.tiff]

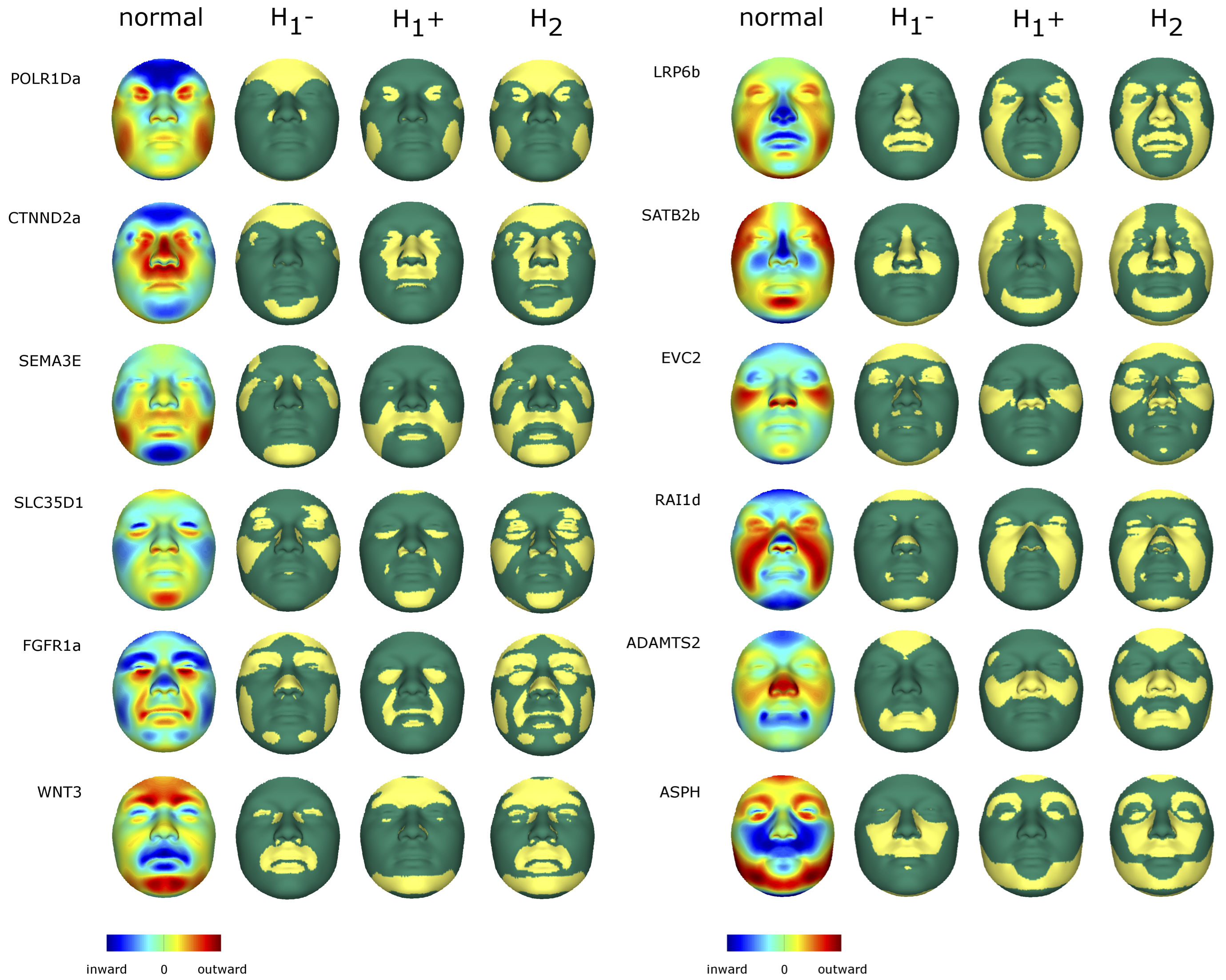

Supplement: Figure S43 — Normal displacements due to candidate genes. PART 1. (TIFF) [file pgen.1004224.s043.tiff]

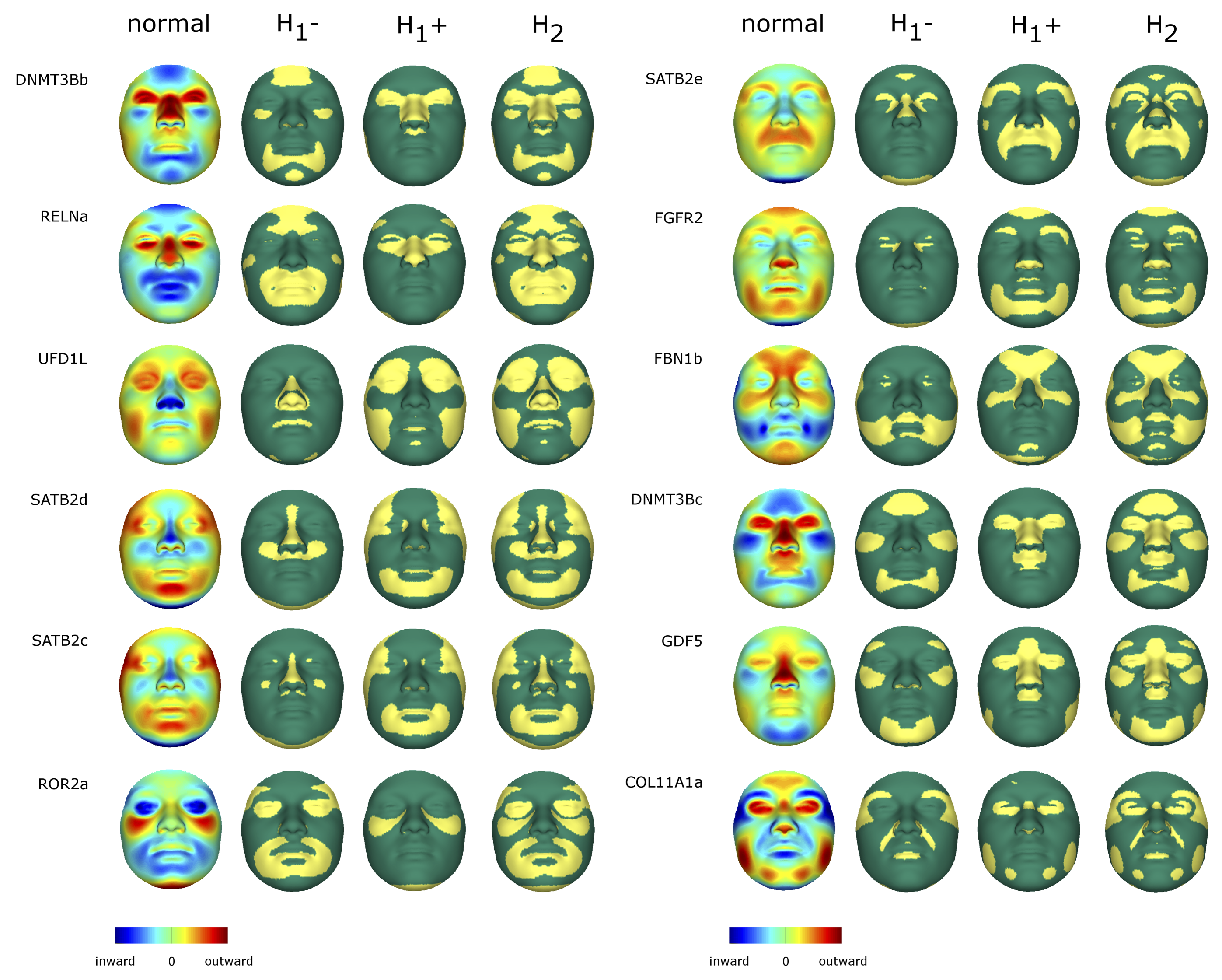

Supplement: Figure S44 — Normal displacements due to candidate genes. PART 2. (TIFF) [file pgen.1004224.s044.tiff]
