# Supplementary material for: Modeling 3D Facial Shape from DNA
Source: PLoS Genet. 2014 Mar 20;10(3):e1004224. doi: 10.1371/journal.pgen.1004224 (PMC3961191; doi:10.1371/journal.pgen.1004224)
Supplement: Table S1 — List of local face shape change parameters (FSCPs) measured. (DOCX) [file pgen.1004224.s045.docx]

| ***Facial Characteristic*** | ***ID*** | ***Explanation*** | | | ***Technical Formulation*** |
| --- | --- | --- | --- | --- | --- |
| Malar flattening | A | Change in surface curvature at the cheekbones | | | $\left\vert C_{Cheekbones}^{B}-C_{Cheekbones}^{A} \right\vert$ |
|  | B | Change in variation or dispersion in position (standard deviation) along the antero-posterior (sagittal) axis of the cheekbones | | | $\left\vert\sigma_{z_{Cheekbones}}^{B}-\sigma_{z_{Cheekbones}}^{A} \right\vert$ |
|  | C | Inward/outward movement of the medial part of the midface, in relation to the lateral part of the lower face | | | $\left\vert N_{MidfaceMedial}-N_{LowerFaceLateral} \right\vert$ |
| Square/round face | A | Change in similarity between facial contours and a square (Fig S30) | | | *Take a border projection of each shape transformation and compare it with a fitted square:*  $\left\vert\sqrt{\sum_{\forall i\in border B} \left( \min_{\forall j\in square B} \left( \Delta\left( P_{i} , P_{j} \right) \right) \right)^{2}}-\sqrt{\sum_{\forall i\in border A} \left( \min_{\forall j\in square A} \left( \Delta\left( P_{i} , P_{j} \right) \right) \right)^{2}} \right\vert$  *The square fitted to each of the morphs is defined by placing the 2 vertical lines at the most lateral positions of the facial border (left and right) and the 2 horizontal lines at the most superior and inferior positions of the facial border.* |
| Micrognathia | A | Inward/outward movement of the chin | | | $\left\vert N_{Chin} \right\vert$ |
|  | B | Area increase/decrease of the size of the chin | | | $\left\vert-log \left( \frac{S_{Chin}^{B}}{S_{Chin}^{A}} \right) \right\vert$ |
|  | C | Change in distance between labiale inferius and gnathion, along the vertical (longitudinal) axis | | | $\left\vert\Delta_{y}\left( \mathrm{LM}_{Labiale Inferius}^{B} , \mathrm{LM}_{Gnathion}^{B} \right)-\Delta_{y}\left( \mathrm{LM}_{Labiale Inferius}^{A} , \mathrm{LM}_{Gnathion}^{A} \right) \right\vert$ |
| Microcephaly | A | Change in size of the circle fitted to the surface of the forehead. The circle is placed in the transverse plane, halfway the glabella and the top of the face (Fig S31) | | | *Take the intersection plane with the facial mesh (through point halfway between glabella and top of head, parallel to the XZ plane), fit a circle through the intersection points, and take radius as approximate for head circumference* |
|  | B | Inward/outward movement of the upper face | | | $\left\vert N_{UpperFace} \right\vert$ |
| Midface retrusion / Flat midface | A | Inward/outward movement of the midface in relation to the inward/outward movement of the upper and lower face | | | $\left\vert N_{UpperFace\cup LowerFace}-N_{Midface} \right\vert$ |
|  | B | Change in surface curvature at the midface | | | $\left\vert C_{Midface}^{B}-C_{Midface}^{A} \right\vert$ |
| Frontal bossing | A | Inward or outward movement of the bilateral parts of the upper face, relative to the movement of the metopic ridge | | | $\left\vert N_{ForeheadBilateral}-N_{MetopicRidge} \right\vert$ |
| Metopic ridge prominent | A | Change in curvature at the surface over the metopic ridge | | | $\left\vert C_{MetopicRidge}^{B}-C_{MetopicRidge}^{A} \right\vert$ |
| Face long | A | Change in height-width ratio of the entire face | | | $\left\vert\log\left( \frac{\sigma_{y_{UpperFace}}^{B}}{\sigma_{x_{UpperFace}}^{B}} \right)-\log\left( \frac{\sigma_{y_{UpperFace}}^{A}}{\sigma_{x_{UpperFace}}^{A}} \right) \right\vert$ |
| Forehead short | A | Change in height of the upper face | | | $\left\vert\sigma_{y_{UpperFace}}^{B}-\sigma_{y_{UpperFace}}^{A} \right\vert$ |
| Supraorbital ridge underdeveloped | A | Inward/outward movement of the supraorbital ridge | | | $\left\vert N_{SupraorbitalRidge} \right\vert$ |
|  | B | Displacement of the supraorbital ridge along the antero-posterior (sagittal) axis. | | | $\left\vert\Delta_{z}\left( \begin{aligned} mean\left( B_{SupraorbitalRidge} \right) , \\ mean\left( A_{SupraorbitalRidge} \right) \end{aligned} \right) \right\vert$ |
| Forehead sloping | A | Inward/outward movement of the upper face | | | $\left\vert N_{UpperFace} \right\vert$ |
|  | B | Change in angle that the antero-posterior (sagittal) axis makes with the anterior surface of the forehead, at the intersection with the sagittal (medial) plane, through the glabella | | | $\left\vert\theta\left( v_{Glabella\to TopForehead}^{B} , y-axis \right)-\theta\left( v_{Glabella\to TopForehead}^{A} , y-axis \right) \right\vert$  *where TopForehead is the most superior point of the intersection line between the ZY-plane through the glabella and the facial mesh.* |
| Forehead narrow/ broad | A | Change in width of the upper face | | | $\left\vert\sigma_{x_{UpperFace}}^{B}-\sigma_{x_{UpperFace}}^{A} \right\vert$ |
| Shallow orbits | A | AND | Inward/outward movement of the orbital ridges | | $\left\vert N_{OrbitalRidges} \right\vert$ |
|  | B |  | Inward/outward movement of the supraorbital ridge | | $\left\vert N_{SupraorbitalRidge} \right\vert$ |
|  | C |  | Inward/outward movement of the infraorbital ridge | | $\left\vert N_{InfraorbitalRidge} \right\vert$ |
|  | D | AND | Change in surface curvature at the orbital ridges | | $\left\vert C_{OrbitalRidges}^{B}-C_{OrbitalRidges}^{A} \right\vert$ |
|  | E |  | Change in surface curvature at the supraorbital ridge | | $\left\vert C_{SupraorbitalRidge}^{B}-C_{SupraorbitalRidge}^{A} \right\vert$ |
|  | F |  | Change in surface curvature at the infraorbital ridge | | $\left\vert C_{InfraorbitalRidge}^{B}-C_{InfraorbitalRidge}^{A} \right\vert$ |
| Superiorly oriented orbits | A | Change in surface curvature at the superior half of the eye, compared to the change in surface curvature at the inferior half of the eye. | | | $\left\vert\left( C_{EyesSuperior}^{B}-C_{EyesSuperior}^{A} \right)-\left( C_{EyesInferior}^{B}-C_{EyesInferior}^{A} \right) \right\vert$ |
|  | B | Inward/outward movement of the superior half of the eye, compared to the inward/outward movement of the inferior half of the eye. | | | $\left\vert N_{EyesSuperior}-N_{EyesInferior} \right\vert$ |
|  | C | Area increase/decrease of the superior half of the eye, compared to the area increase/decrease of the inferior half of the eye. | | | $\left\vert-log \left( \frac{S_{EyesSuperior}^{B}}{S_{EyesSuperior}^{A}} \right)+\log\left( \frac{A_{EyesInferior}^{B}}{A_{EyesInferior}^{A}} \right) \right\vert$ |
| Palpebral fissures downslanted | A | Change in distance along the vertical (longitudinal) axis from the average position of the medial half of the eye, to the average position of the lateral half of the eye. | | | $\left\vert\Delta_{y}\left( \begin{aligned} mean\left( B_{EyesMedial} \right), \\ mean(B_{EyesLateral}) \end{aligned} \right)-\Delta_{y}\left( \begin{aligned} mean\left( A_{EyesMedial} \right), \\ mean(A_{EyesLateral}) \end{aligned} \right) \right\vert$ |
|  | B | Change in angle between the principal axis of the left eye and the principal axis of the right eye | | | $\left\vert\theta\left( {PC1}_{LeftEye}^{B} , {PC1}_{RightEye}^{B} \right)-\theta\left( {PC1}_{LeftEye}^{A} , {PC1}_{RightEye}^{A} \right) \right\vert$ |
| Eyes widely spaced | A | Change in interpupillary distance | | | $\left\vert\Delta\left( \mathrm{LM}_{Right Pupil}^{B} , \mathrm{LM}_{Left Pupil}^{B} \right)-\Delta\left( \mathrm{LM}_{Right Pupil}^{A} , \mathrm{LM}_{Left Pupil}^{A} \right) \right\vert$ |
|  | B | Change in outer canthal distance | | | $\left\vert\Delta\left( \begin{aligned} \mathrm{LM}_{Right Endocanthion}^{B} , \\ \mathrm{LM}_{Left Endocanthion}^{B} \end{aligned} \right)-\Delta\left( \begin{aligned} \mathrm{LM}_{Right Endocanthion}^{A} , \\ \mathrm{LM}_{Left Endocanthion}^{A} \end{aligned} \right) \right\vert$ |
|  | C | Change in distance along the medio-lateral (horizontal) axis from the average position of the left eye, to the average position of the right eye. | | | $\left\vert\Delta_{x}\left( \begin{aligned} mean\left( B_{EyesLeft} \right), \\ mean(B_{EyesRight}) \end{aligned} \right)-\Delta_{x}\left( \begin{aligned} mean\left( A_{EyesLeft} \right), \\ mean(A_{EyesRight}) \end{aligned} \right) \right\vert$ |
| Proptosis | A | AND | Movement of the eyes along the antero-posterior (sagittal) axis | | $\left\vert\Delta_{z}\left( mean\left( B_{Eyes} \right) , mean\left( A_{Eyes} \right) \right) \right\vert$ |
|  | B |  | AND | Movement of the lateral half of the eyes along the antero-posterior (sagittal) axis. | $\left\vert\Delta_{z}\left( \begin{aligned} mean\left( B_{EyesLateral} \right) , \\ mean\left( A_{EyesLateral} \right) \end{aligned} \right) \right\vert$ |
|  | C |  |  | Movement of the medial half of the eyes along the antero-posterior (sagittal) axis. | $\left\vert\Delta_{z}\left( \begin{aligned} mean\left( B_{EyesMedial} \right) , \\ mean\left( A_{EyesMedial} \right) \end{aligned} \right) \right\vert$ |
|  | D |  | AND | Movement of the superior half of the eyes along the antero-posterior (sagittal) axis. | $\left\vert\Delta_{z}\left( \begin{aligned} mean\left( B_{EyesSuperior} \right) , \\ mean\left( A_{EyesSuperior} \right) \end{aligned} \right) \right\vert$ |
|  | E |  |  | Movement of the inferior half of the eyes along the antero-posterior (sagittal) axis. | $\left\vert\Delta_{z}\left( \begin{aligned} mean\left( B_{EyesInferior} \right) , \\ mean\left( A_{EyesInferior} \right) \end{aligned} \right) \right\vert$ |
|  | F |  | NOT | Change in distance along the antero-posterior (sagittal) axis from the average position of the superior half of the eyes, to the average position of the inferior half of the eyes. (This indicates ‘superiorly oriented orbits’) | $\left\vert\Delta_{z}\left( \begin{aligned} mean\left( B_{EyesSuperior} \right), \\ mean(B_{EyesInferior}) \end{aligned} \right)-\Delta_{z}\left( \begin{aligned} mean\left( A_{EyesSuperior} \right), \\ mean(A_{EyesInferior}) \end{aligned} \right) \right\vert$ |
| Nasal ridge narrow | A | Change in width of the nasal ridge | | | $\left\vert\sigma_{x_{NasalRidge}}^{B}-\sigma_{x_{NasalRidge}}^{A} \right\vert$ |
|  | B | AND | Change in curvature at the surface over the nasal ridge, compared to the change in surface curvature at the paranasal tissues | | $\left\vert\left( C_{NasalRidge}^{B}-C_{NasalRidge}^{A} \right)-\left( C_{ParanasalTissues}^{B}-C_{ParanasalTissues}^{A} \right) \right\vert$ |
|  | C |  | Change in surface curvature at the nasal ridge | | $\left\vert C_{NasalRidge}^{B}-C_{NasalRidge}^{A} \right\vert$ |
|  | D |  | Change in surface curvature at the paranasal tissues | | $\left\vert C_{ParanasalTissues}^{B}-C_{ParanasalTissues}^{A} \right\vert$ |
| Nasal ridge retruded | A | Inward/outward movement of the nasal ridge | | | $\left\vert N_{NasalRidge} \right\vert$ |
| Nasal bridge depressed | A | Inward/outward movement of the nasal bridge | | | $\left\vert N_{NasalBridge} \right\vert$ |
| Nasal bridge wide | A | Change in surface curvature at the nasal bridge | | | $\left\vert C_{NasalBridge}^{B}-C_{NasalBridge}^{A} \right\vert$ |
|  | B | Change in width of the nasal bridge | | | $\left\vert\sigma_{x_{NasalBridge}}^{B}-\sigma_{x_{NasalBridge}}^{A} \right\vert$ |
| Nose wide | A | Change in distance between alar curvature left and right | | | $\left\vert\Delta\left( \begin{aligned} \mathrm{LM}_{Right Alar Curvature}^{B} , \\ \mathrm{LM}_{Left Alar Curvature}^{B} \end{aligned} \right)-\Delta\left( \begin{aligned} \mathrm{LM}_{Right Alar Curvature}^{A} , \\ \mathrm{LM}_{Left Alar Curvature}^{A} \end{aligned} \right) \right\vert$ |
|  | B | Change in distance between alare left and right | | | $\left\vert\Delta\left( \mathrm{LM}_{Right Alare}^{B} , \mathrm{LM}_{Left Alare}^{B} \right)-\Delta\left( \mathrm{LM}_{Right Alare}^{A} , \mathrm{LM}_{Left Alare}^{A} \right) \right\vert$ |
| Nose snubbed | A | Change in angle between columella (represented as a vector from subnasale to pronasale) and philtrum (represented as a vector from subnasale to labiale superius) | | | $\left\vert\theta\left( \begin{aligned} v_{Subnasale\to Pronasale}^{B} , \\ v_{Subnasale\to Labiale Superius}^{B} \end{aligned} \right)-\theta\left( \begin{aligned} v_{Subnasale\to Pronasale}^{A} , \\ v_{Subnasale\to Labiale Superius}^{A} \end{aligned} \right) \right\vert$ |
| Nares anteverted | A | Change in distance along the vertical (longitudinal) axis between subnasale and pronasale | | | $\left\vert\Delta_{y}\left( \mathrm{LM}_{Subnasale}^{B} , \mathrm{LM}_{Pronasale}^{B} \right)-\Delta_{y}\left( \mathrm{LM}_{Subnasale}^{A} , \mathrm{LM}_{Pronasale}^{A} \right) \right\vert$ |
| Cleft lip | A | Area increase/decrease of the philtrum | | | $\left\vert-log \left( \frac{S_{Philtrum}^{B}}{S_{Philtrum}^{A}} \right) \right\vert$ |
|  | B | Inward/outward movement of the philtrum | | | $\left\vert N_{Philtrum} \right\vert$ |
|  | C | Change in surface curvature of the philtrum | | | $\left\vert C_{Philtrum}^{B}-C_{Philtrum}^{A} \right\vert$ |
|  | D | Area increase/decrease of the nose | | | $\left\vert-log \left( \frac{S_{Nose}^{B}}{S_{Nose}^{A}} \right) \right\vert$ |
|  | E | Inward/outward movement of the nose | | | $\left\vert N_{Nose} \right\vert$ |
|  | F | Change in surface curvature of the nose | | | $\left\vert C_{Nose}^{B}-C_{Nose}^{A} \right\vert$ |
| Mouth wide | A | Change in distance between left and right chelion | | | $\left\vert\Delta\left( \mathrm{LM}_{Right Chelion}^{B} , \mathrm{LM}_{Left Chelion}^{B} \right)-\Delta\left( \mathrm{LM}_{Right Chelion}^{A} , \mathrm{LM}_{Left Chelion}^{A} \right) \right\vert$ |
| Vermilion upper lip thick | A | Area increase/decrease of the upper lip (upper vermilion) | | | $\left\vert-log \left( \frac{S_{UpperLip}^{B}}{S_{UpperLip}^{A}} \right) \right\vert$ |
| Vermilion lower lip thick | A | Area increase/decrease of the lower lip (lower vermilion) | | | $\left\vert-log \left( \frac{S_{LowerLip}^{B}}{S_{LowerLip}^{A}} \right) \right\vert$ |
| Lips thick | A | Area increase/decrease of the lips | | | $\left\vert-log \left( \frac{S_{Lips}^{B}}{S_{Lips}^{A}} \right) \right\vert$ |
| Maxilla prominent | A | Movement of the midface along the antero-posterior (sagittal) axis | | | $\left\vert\Delta_{z}\left( mean\left( B_{Midface} \right) \right)-\Delta_{z}\left( mean\left( A_{Midface} \right) \right) \right\vert$ |
|  | B | Inward/outward movement of the medial part of the midface, in relation to the lateral part of the lower face | | | $\left\vert N_{MidfaceMedial}-N_{LowerFaceLateral} \right\vert$ |
| Philtrum long | A | Change in length of the philtrum (represented as a vector from subnasale to labiale superius) | | | $\left\vert\Delta\left( \mathrm{LM}_{Subnasale}^{B} , \mathrm{LM}_{Labiale Superius}^{B} \right)-\Delta\left( \mathrm{LM}_{Subnasale}^{A} , \mathrm{LM}_{Labiale Superius}^{A} \right) \right\vert$ |
| Sunken cheeks | A | Area increase/decrease of the cheeks | | | $\left\vert-log \left( \frac{S_{Cheeks}^{B}}{S_{Cheeks}^{A}} \right) \right\vert$ |
| Large eyes | A | Area increase/decrease of the eyes | | | $\left\vert-log \left( \frac{S_{Eyes}^{B}}{S_{Eyes}^{A}} \right) \right\vert$ |
|  | B | Area increase/decrease of the eyes, projected onto the coronal (frontal) plane | | | $\left\vert-log \left( \frac{S_{{xy}_{Eyes}}^{B}}{S_{{xy}_{Eyes}}^{A}} \right) \right\vert$ |
| Large nose tip | A | Area increase/decrease of the nasal tip | | | $\left\vert-log \left( \frac{S_{NasalTip}^{B}}{S_{NasalTip}^{A}} \right) \right\vert$ |
|  | B | Area increase/decrease of the nasal tip, projected onto the coronal (frontal) plane | | | $\left\vert-log \left( \frac{S_{{xy}_{NasalTip}}^{B}}{S_{{xy}_{NasalTip}}^{A}} \right) \right\vert$ |
|  | D | Inward/outward movement of the nasal tip | | | $\left\vert N_{NasalTip} \right\vert$ |

Notes: C = curvature, S = area, LM = landmark, N = normal displacement, P = point, B = shape transformation -X times the standard deviation of RIP values, A = shape transformation +X times the standard deviation of RIP values, σ_x,y,z_ the standard deviation of quasi-landmarks in the x,y or z direction (used as surrogate for length and width measurements), θ = angle measurement, $\Delta$ = distance between two points, $\Delta_{\boldsymbol{x},\boldsymbol{y},\boldsymbol{z}}$ = distance in the X, Y, or Z direction, $\boldsymbol{v}_{\boldsymbol{P}_{\boldsymbol{1}}\to\boldsymbol{P}_{\boldsymbol{2}}}$ = vector from point $\boldsymbol{P}_{\boldsymbol{1}}$ to point $\boldsymbol{P}_{\boldsymbol{2}}$.
