# Supplementary material for: Modeling 3D Facial Shape from DNA
Source: PLoS Genet. 2014 Mar 20;10(3):e1004224. doi: 10.1371/journal.pgen.1004224 (PMC3961191; doi:10.1371/journal.pgen.1004224)
Supplement: Table S2 — ANOVA analysis of genotypes on facial morphology using RIP variables. The Table is sorted from low to high p-value on the three group ANOVA results. (DOCX) [file pgen.1004224.s046.docx]

| Gene Symbol | SNP symbol in facial figures | reference SNP ID | chromo-some | position | F statistic | p-value | A | B | YRI | CEU | DELTA |
| --- | --- | --- | --- | --- | --- | --- | --- | --- | --- | --- | --- |
| *POLR1D* | POLR1Da | rs507217 | 13 | 27106517 | 6.98 | **0** | C | A | 0.858 | 0.398 | 0.46 |
| *CTNND2* | CTNND2a | rs2277054 | 5 | 11213043 | 5.84 | **0.004** | G | A | 0.013 | 0.535 | 0.522 |
| *SEMA3E* | SEMA3E | rs2709922 | 7 | 82863254 | 6 | **0.004** | G | A | 0.119 | 0.707 | 0.588 |
| *SLC35D1* | SLC35D1 | rs1074265 | 1 | 67263852 | 5.36 | **0.005** | T | A | 0.625 | 0.1 | 0.525 |
| *FGFR1* | FGFR1a | rs13267109 | 8 | 38496214 | 4.5 | **0.01** | G | A | 0.78 | 0.228 | 0.552 |
| *WNT3* | WNT3 | rs199501 | 17 | 42217772 | 4.62 | **0.011** | G | A | 0.252 | 0.788 | 0.536 |
| *LRP6* | LRP6b | rs2724626 | 12 | 11721982 | 4.19 | **0.015** | A | C | 0.108 | 0.883 | 0.775 |
| *SATB2* | SATB2b | rs1357582 | 2 | 200287112 | 4.27 | **0.015** | G | A | 0.143 | 0.761 | 0.618 |
| *EVC2* | EVC2 | rs10001971 | 4 | 5689700 | 4.26 | **0.016** | A | G | 0.796 | 0.042 | 0.754 |
| *RAI1* | RAI1d | rs4925108 | 17 | 17590148 | 4 | **0.017** | A | G | 0.009 | 0.677 | 0.668 |
| *ADAMTS2* | ADAMTS2 | rs3822601 | 5 | 178554182 | 3.75 | **0.021** | A | G | 0.587 | 0.066 | 0.521 |
| *ASPH* | ASPH | rs4738909 | 8 | 62650091 | 3.39 | **0.033** | C | A | 0.867 | 0.283 | 0.584 |
| *DNMT3B* | DNMT3Bb | rs2424905 | 20 | 30816588 | 3.42 | **0.037** | G | A | 0.95 | 0.358 | 0.592 |
| *RELN* | RELNa | rs471360 | 7 | 103168085 | 3.15 | **0.044** | A | G | 0.55 | 0.108 | 0.442 |
| *UFD1L* | UFD1L | rs2073730 | 22 | 17817946 | 3.18 | **0.045** | G | C | 1 | 0.491 | 0.509 |
| *SATB2* | SATB2d | rs6759018 | 2 | 200329008 | 3.12 | **0.045** | G | A | 0.115 | 0.695 | 0.58 |
| *SATB2* | SATB2c | rs4530349 | 2 | 200329665 | 2.88 | **0.054** | A | G | 0.204 | 0.704 | 0.5 |
| *ROR2* | ROR2a | rs7029814 | 9 | 93602502 | 2.74 | **0.064** | G | A | 0.978 | 0.389 | 0.589 |
| *SATB2* | SATB2e | rs4673339 | 2 | 199981621 | 2.68 | **0.068** | G | A | 0.004 | 0.456 | 0.452 |
| *FGFR2* | FGFR2 | rs2278202 | 10 | 123233187 | 2.55 | **0.081** | G | A | 0.925 | 0.42 | 0.505 |
| *FBN1* | FBN1b | rs6493315 | 15 | 46354306 | 2.33 | **0.1** | G | A | 0.774 | 0.196 | 0.578 |
| *DNMT3B* | DNMT3Bc | rs2424928 | 20 | 30852297 | 2.28 | **0.103** | G | A | 0.929 | 0.403 | 0.526 |
| *GDF5* | GDF5 | rs143384 | 20 | 33489170 | 2.24 | **0.106** | G | A | 1 | 0.358 | 0.642 |
| *COL11A1* | COL11A1a | rs11164669 | 1 | 103326755 | 2.25 | **0.106** | G | A | 0.064 | 0.629 | 0.565 |
| *DHCR7* | DHCR7 | rs11603330 | 11 | 70831107 | 2.06 | 0.122 | C | A | 0.889 | 0.279 | 0.61 |
| *FGFR1* | FGFR1b | rs7818839 | 8 | 38607758 | 2.03 | 0.134 | G | A | 0.106 | 0.681 | 0.575 |
| *RPS19* | RPS19 | rs7254214 | 19 | 47060578 | 2.03 | 0.135 | G | A | 0.959 | 0.509 | 0.45 |
| *ROR2* | ROR2b | rs7037255 | 9 | 93736775 | 1.98 | 0.136 | G | A | 0.052 | 0.619 | 0.567 |
| *DNMT3B* | DNMT3Ba | rs1997797 | 20 | 30851615 | 2.02 | 0.138 | G | C | 0.864 | 0.331 | 0.533 |
| *RAI1* | RAI1c | rs4315391 | 17 | 17270453 | 2 | 0.141 | G | A | 0.093 | 0.704 | 0.611 |
| *CHD7* | CHD7 | rs10092214 | 8 | 61889433 | 1.95 | 0.149 | G | A | 0.996 | 0.58 | 0.416 |
| *GNAS* | GNAS | rs6123837 | 20 | 56898966 | 1.88 | 0.152 | G | A | 0.991 | 0.588 | 0.403 |
| *NIPBL* | NIPBL | rs300063 | 5 | 37059655 | 1.68 | 0.185 | A | C | 0.903 | 0.414 | 0.489 |
| *SIL1* | SIL1 | rs10074485 | 5 | 138523427 | 1.63 | 0.192 | G | A | 0.31 | 0.903 | 0.593 |
| *CTNND2* | CTNND2c | rs2561627 | 5 | 11708192 | 1.62 | 0.201 | G | A | 0.844 | 0.261 | 0.583 |
| *RAI1* | RAI1a | rs2955382 | 17 | 17888435 | 1.6 | 0.201 | A | G | 0.137 | 0.642 | 0.505 |
| *RSPO2* | RSPO2 | rs2514838 | 8 | 109118075 | 1.63 | 0.203 | G | A | 0.996 | 0.518 | 0.478 |
| *WT1* | WT1 | rs5030317 | 11 | 32366913 | 1.63 | 0.204 | G | C | 0.849 | 0.248 | 0.601 |
| *SKI* | SKI | rs2843159 | 1 | 2225532 | 1.6 | 0.204 | A | G | 0.562 | 0.137 | 0.425 |
| *DPYD* | DPYDb | rs526645 | 1 | 97521968 | 1.56 | 0.209 | G | A | 0 | 0.417 | 0.417 |
| *GLI3* | GLI3a | rs10951667 | 7 | 42118246 | 1.5 | 0.225 | G | A | 0.155 | 0.611 | 0.456 |
| *FBN1* | FBN1a | rs16961205 | 15 | 46669609 | 1.44 | 0.239 | C | A | 0.491 | 1 | 0.509 |
| *FANCA* | FANCA | rs10852623 | 16 | 88392743 | 1.41 | 0.241 | G | A | 0.841 | 0.416 | 0.425 |
| *RELN* | RELNb | rs7799028 | 7 | 103133462 | 1.38 | 0.245 | A | C | 0.755 | 0.242 | 0.513 |
| *COL1A1* | COL1A1 | rs1934709 | 1 | 102740624 | 1.38 | 0.251 | A | G | 0.235 | 0.885 | 0.65 |
| *DPYD* | DPYDa | rs12568335 | 1 | 98046720 | 1.32 | 0.27 | G | A | 0.119 | 0.58 | 0.461 |
| *CTNND2* | CTNND2d | rs7733427 | 5 | 11896303 | 1.15 | 0.316 | G | A | 0 | 0.628 | 0.628 |
| *WT1* | WT1b | rs5030320 | 11 | 32366578 | 1.1 | 0.333 | G | A | 0.823 | 0.288 | 0.535 |
| *FREM2* | FREM2a | rs2496425 | 13 | 38162690 | 1.06 | 0.343 | G | A | 0.854 | 0.301 | 0.553 |
| *CCBE1* | CCBE1 | rs2564464 | 18 | 55387663 | 1.05 | 0.346 | C | A | 0.167 | 0.667 | 0.5 |
| *FLNB* | FLNB | rs1127745 | 3 | 58487277 | 1.04 | 0.359 | G | A | 0.677 | 0.088 | 0.589 |
| *LRP6* | LRP6c | rs3741800 | 12 | 12387226 | 0.99 | 0.364 | A | G | 0.035 | 0.535 | 0.5 |
| *FRAS1* | FRAS1 | rs345528 | 4 | 79448326 | 0.99 | 0.372 | G | A | 0.447 | 0.004 | 0.443 |
| *ECE1* | ECE1 | rs3026900 | 1 | 21432667 | 0.93 | 0.39 | G | A | 0.487 | 0.035 | 0.452 |
| *PEX3* | PEX3b | rs9403540 | 6 | 144334280 | 0.92 | 0.4 | C | A | 0.004 | 0.536 | 0.532 |
| *PEX3* | PEX3a | rs161062 | 6 | 143843526 | 0.82 | 0.441 | G | A | 0.177 | 0.69 | 0.513 |
| *SNRPN* | SNRPN | rs12591149 | 15 | 22727610 | 0.8 | 0.445 | G | A | 0.947 | 0.478 | 0.469 |
| *POLR1D* | POLR1Db | rs542610 | 13 | 27133069 | 0.76 | 0.471 | A | G | 0.398 | 0.805 | 0.407 |
| *HDAC4* | HDAC4a | rs10207474 | 2 | 239720868 | 0.69 | 0.504 | G | A | 0.425 | 0.019 | 0.406 |
| *GLI3* | GLI3b | rs11772482 | 7 | 42002148 | 0.64 | 0.536 | G | A | 0.85 | 0.434 | 0.416 |
| *FGFR2* | FGFR2b | rs2912755 | 10 | 123252698 | 0.53 | 0.596 | G | A | 0.531 | 0.035 | 0.496 |
| *TBX1* | TBX1 | rs2301558 | 22 | 18131829 | 0.45 | 0.638 | A | G | 0.593 | 0.181 | 0.412 |
| *ADAMTS10* | ADAMTS10 | rs10401300 | 19 | 8579881 | 0.44 | 0.65 | G | A | 0.554 | 0.018 | 0.536 |
| *SATB2* | SATB2a | rs1014497 | 2 | 199949474 | 0.3 | 0.741 | A | G | 0.264 | 0.708 | 0.444 |
| *BRAF* | BRAF | rs10487888 | 7 | 140145576 | 0.28 | 0.76 | A | G | 0.004 | 0.522 | 0.518 |
| *EVC2* | EVC2 | rs7670299 | 4 | 5627041 | 0.23 | 0.795 | G | A | 0.808 | 0.307 | 0.501 |
| *FGFR1* | FGFR1c | rs6474464 | 8 | 38617794 | 0.22 | 0.802 | A | G | 0.279 | 0.842 | 0.563 |
| *CTNND2* | CTNND2b | rs249237 | 5 | 11494822 | 0.22 | 0.805 | G | A | 0.721 | 0.084 | 0.637 |
| *COL11A1* | COL11A1b | rs6577351 | 1 | 103325508 | 0.13 | 0.878 | G | A | 0.606 | 0.088 | 0.518 |
| *RAI1* | RAI1b | rs8079502 | 17 | 17262921 | 0.11 | 0.89 | G | A | 0.199 | 0.883 | 0.684 |
| *GLI3* | GLI3c | rs6969239 | 7 | 42001356 | 0.11 | 0.895 | G | A | 0.933 | 0.526 | 0.407 |
| *HDAC4* | HDAC4b | rs7573680 | 2 | 239834014 | 0.1 | 0.9 | A | G | 0.633 | 0.205 | 0.428 |
| *LMNA* | LMNA | rs505058 | 1 | 154372809 | 0.1 | 0.908 | G | A | 0.73 | 0.066 | 0.664 |
| *FREM2* | FREM2b | rs990909 | 13 | 38188569 | 0.08 | 0.921 | A | G | 0.142 | 0.642 | 0.5 |
| *LRP6* | LRP6a | rs12823243 | 12 | 12220953 | 0.06 | 0.948 | T | A | 0.925 | 0.5 | 0.425 |
| *NSD1* | NSD1 | rs12660023 | 5 | 176633491 | 0.01 | 0.987 | G | A | 0.792 | 0.19 | 0.602 |
