# Supplementary material for: Modeling 3D Facial Shape from DNA
Source: PLoS Genet. 2014 Mar 20;10(3):e1004224. doi: 10.1371/journal.pgen.1004224 (PMC3961191; doi:10.1371/journal.pgen.1004224)
Supplement: Table S3 — RIP effect-size statistics. (DOCX) [file pgen.1004224.s047.docx]

|  | Overall partial R^2^ | mean | maximum | standard deviation | 1st quartile | median | 3rd quartile |
| --- | --- | --- | --- | --- | --- | --- | --- |
| POLR1Da | **1.24** | 1.58 | **7.06** | 1.42 | 0.55 | 1.15 | 2.10 |
| CTNND2a | **1.60** | 2.07 | **8.10** | 1.58 | 0.90 | 1.60 | 2.76 |
| SEMA3E | **1.42** | 1.65 | **6.39** | 1.27 | 0.77 | 1.35 | 2.18 |
| SLC35D1 | **1.44** | 1.88 | **11.68** | 1.55 | 0.84 | 1.42 | 2.45 |
| FGFR1a | **2.82** | 3.98 | **15.16** | 2.69 | 1.91 | 3.41 | 5.27 |
| WNT3 | **1.94** | 2.49 | **9.95** | 2.32 | 0.74 | 1.47 | 3.81 |
| LRP6b | **1.91** | 2.38 | **10.10** | 2.14 | 0.81 | 1.71 | 3.15 |
| SATB2b | **3.31** | 4.37 | **10.09** | 2.30 | 2.61 | 4.01 | 5.96 |
| EVC2 | **1.87** | 2.50 | **13.70** | 2.45 | 0.72 | 1.61 | 3.39 |
| RAI1d | **1.14** | 1.46 | **5.34** | 1.03 | 0.68 | 1.26 | 2.00 |
| ADAMTS2 | **1.60** | 2.08 | **15.28** | 2.21 | 0.59 | 1.31 | 2.68 |
| ASPH | **2.17** | 2.71 | **9.25** | 2.08 | 1.04 | 2.12 | 3.89 |
| DNMT3Bb | **1.35** | 1.81 | **9.91** | 1.62 | 0.65 | 1.33 | 2.48 |
| RELNa | **1.93** | 2.59 | **11.31** | 2.21 | 0.94 | 1.89 | 3.79 |
| UFD1L | **2.95** | 3.71 | **17.17** | 2.78 | 1.63 | 3.23 | 4.89 |
| SATB2d | **2.11** | 2.62 | **6.57** | 1.39 | 1.49 | 2.45 | 3.67 |
| SATB2c | **2.13** | 2.69 | **8.00** | 1.60 | 1.58 | 2.33 | 3.50 |
| ROR2a | **1.79** | 2.34 | **10.51** | 1.90 | 1.07 | 1.91 | 3.25 |
| SATB2e | **1.81** | 2.30 | **8.40** | 1.80 | 0.91 | 1.69 | 3.22 |
| FGFR2 | **1.56** | 1.90 | **4.66** | 0.93 | 1.15 | 1.76 | 2.48 |
| FBN1b | **1.24** | 1.63 | **9.60** | 1.26 | 0.67 | 1.43 | 2.24 |
| DNMT3Bc | **1.18** | 1.65 | **6.83** | 1.56 | 0.41 | 1.05 | 2.51 |
| GDF5 | **1.99** | 2.48 | **13.17** | 2.46 | 0.84 | 1.69 | 3.31 |
| COL11A1a | **0.93** | 1.22 | **4.64** | 0.76 | 0.67 | 1.09 | 1.58 |
| Sex | **12.92** | 14.08 | **38.21** | 9.29 | 6.99 | 12.47 | 20.23 |
| Ancestry | **9.55** | 9.86 | **40.83** | 7.97 | 3.78 | 7.82 | 13.55 |
