# Supplementary material for: Modeling 3D Facial Shape from DNA
Source: PLoS Genet. 2014 Mar 20;10(3):e1004224. doi: 10.1371/journal.pgen.1004224 (PMC3961191; doi:10.1371/journal.pgen.1004224)
Supplement: Table S4 — Empirical p-values under 10,000 permutations for the local FSCPs listed in TableS1 tested for the 24 candidate genes, sex and ancestry. (DOCX) [file pgen.1004224.s048.docx]

Notes: Green cell, significant (p<0.05) effect. Yellow cell, significant effect (p<0.05). Red cell, non-significant effect (p>=0.05). White cell, non-significant effect (p>=0.05)
