# Supplementary material for: Modeling 3D Facial Shape from DNA
Source: PLoS Genet. 2014 Mar 20;10(3):e1004224. doi: 10.1371/journal.pgen.1004224 (PMC3961191; doi:10.1371/journal.pgen.1004224)
Supplement: Text S1 — Supporting materials text (1) Bootstrapped response = based imputation modeling (BRIM), (2) Empirical Analysis of BRIM, (3) Facial Characteristics, (4) Extended Results: Sex, Ancestry, and Gene Effects. (DOCX) [file pgen.1004224.s049.docx]

## SUPPORTING materials TEXT 1 (Text S1)

Claes et al., “Modeling 3D facial shape from DNA”

## 1. Bootstrapped response-based imputation modeling (BRIM)

### *1.1 Regression Analysis*

Ordinary regression [1,2] only assumes or allows errors in the response variable(s) and will be detrimentally influenced by inaccurate predictor variables, lowering the stability, efficiency, and statistical power. More advanced regression models, such as, partial least squares regression (PLSR) [3,4], allow imprecision in predictors as well as in responses. It is the case with all regression methods that high levels of error in the predictors reduce the statistical power of the association testing and lowering predictive power. We developed a bootstrapped response-based imputation modeling (BRIM) technique to overcome the limitations of traditional relationship modeling methods. BRIM, an extension of current regression techniques, uses response variables to refine, filter, and transform one or more predictor variables. The output of BRIM is a new type of variable: the response-based imputed predictor (RIP) variable. This is a hybrid or bridging variable that combines information from both the predictor and response variables, creating a novel variable space. From the point-of-view of the response variable, the RIP variable is a supervised recoding of a multivariate response variable into a simpler univariate variable. Thus, the RIP variable can be used to test associations against the predictors and can also be used to visualize the predictor effects on the multivariate-response. From the point-of-view of the predictor, the RIP is a response-based transformation of a likely less precise predictor variable that recovers response-specific information and allows, for example, the transformation of discretely coded (categorical) predictors into continuously distributed RIP variables.

### *2.2 Generating RIP variables*

Assume a set of observations $O=\left\{ o_{k}| k=1,\ldots, K \right\}$ comprising a set of predictor values $X=\left\{ x_{kp}| k=1,\ldots, K; p=1,\ldots, P \right\}$ and a set of response values$Y=\left\{ y_{kq}| k=1,\ldots, K;q=1,\ldots, Q \right\}$. Without loss of generality, we assume a linear Partial Least Squares Regression (PLSR) model$Y=MX$. Here$M$, is the relationship between $X$ and$Y.$ Based on a “leave-N-out” (LNO) approach the set of observations is divided into a training set $O_{Tr}$ and a test set $O_{Te}$ as illustrated in Figure S1. In this work, a leave-one-out (LOO) scheme was applied: each observation is removed, in turn, from the set of observations and used as a test case, while the rest are used as training cases. The training set of observations$O_{Tr}$ is used to learn the PLSR model and to establish the relationship$M$. For every predictor in this relationship a “path is drawn” or a direction is established in the response-space, which explains the variation in the responses caused by the particular predictor, which is known as the regression line and referred to as a predictor-path. This concept is illustrated in Figure S2. Moving along such a path will change the response values in function of a particular predictor value. For example, if the predictor is sex and the response is facial morphology, moving along such a sex-path will cause the face to change from male to female and vice-versa.

For all the observations in the test set$O_{Te}$, a response-based predictor value is imputed as follows: first a point of reference or a reference-response on the predictor-path is chosen, for which we took the point of origin in the response-space after centering all the response variables. Subsequently, in a multivariate case, the vector of a test-response to this reference-response is decomposed into a component perpendicular and a component parallel to the predictor-path. The component perpendicular to the path is known as the response-residual. Because of the perpendicular nature the magnitude of this component, measures the difference between the reference-response and the test-response independent from any difference in predictor value (taking out the effect of the predictor). For example, in the case of sex and faces, the difference between a reference-face and a test-face would then be measured independent of their difference in sex. As such the distance between a brother and a sister, e.g., might be small. However, the component of interest is the parallel component, which measures the difference between both the test- and reference-response, solely in terms of the effect of the predictor. In the example of sex and faces, this parallel component’s signed magnitude measures the difference in sex independent from other facial differences and thus generates a facial-based sex difference. The signed Mahalanobis distance of the parallel component was taken as the response-based imputed predictor (RIP) value. Other distances as well as other measures like angles are possible as well.

*Bootstrapping:* The RIP variables replace the predictor variables and the whole process can be repeated again as depicted in Figure S1. After each repeating cycle or iteration, the predictor-path is refined and the RIP values are updated until no more change is observed and the whole process has converged. The advantage of bootstrapping is twofold. The estimated RIP values improve themselves over subsequent iterations leading to an increased correction of potential errors in the predictor values. This also leads to refined relationship estimation. Additionally, when conditioning on confounding variables an improved conditioning effect over subsequent iterations is observed.

*Nested imputation*: When iteratively constructing RIP values as outlined in Figure S3, there is a circular influence of test-responses on themselves creating an additional dependency, besides the true relationship, between the predictor values and the resulting RIP values. The additional dependency, notwithstanding the LOO setup, is due to the iterative nature of BRIM. Consider two responses A and B. In the first iteration A is influencing B, when B is used as test-response. In the subsequent iteration the RIP value of B, which was influenced by A, is influencing A when A is used as a test-response, hence the circular influence. The solution to avoid the additional dependency is to create a “true” test set in a “BRIM” analysis. Each observation is removed, in turn, from the set of observations and used as a test observation, while the rest are training observations. To be valid, the full BRIM analysis must be completed using the training set ONLY, which requires a second or nested LOO. The BRIM analysis is a necessary component of the technique; otherwise relationships between predictors and responses are artificially “boosted”, such that significant relationships cannot be separated from non-significant relationships and the true relationship cannot be obtained. Consequently, all of the BRIM analyses we perform are BRIM.

*Multiple and partial BRIM:* When presented with more than one predictor, both multiple and partial BRIM analyses are possible. This is similar to a traditional multiple and partial regression analysis. A multiple BRIM analysis implies the joint “brimming” of more than one predictor variable. The resulting RIP variables are uncorrelated and provide the means to analyze the multiple (joint) effects of the predictors onto the responses. For example, the multiple effects of sex and genomic ancestry on facial morphology were obtained by using a multiple BRIM analysis. The resulting RIP variables for both sex (RIP-S) and genomic ancestry (RIP-A) are uncorrelated and code for facial sex and facial ancestry effects respectively. A partial BRIM analysis implies the single “brimming” of one predictor variable conditioned on other predictor variables. The conditioning predictors can either be predictor variables or previously “brimmed” RIP variables. The resulting RIP variable is uncorrelated to the conditioning predictors and provides the means to analyze the partial effect of the predictor onto the responses. Note that in a partial BRIM analysis the conditioning predictors themselves are not updated, forcing the predictor of interest to update such that it becomes as independent as possible from the conditioning predictors. This is in contrast to the multiple BRIM analysis in which all predictors are updated in regard to each other. For example, finding the effect of a genotype independent from sex and ancestry onto facial morphology was obtained using a partial BRIM analysis. Here, sex and genomic ancestry or RIP-S and RIP-A can be used as conditioning variables and are not allowed to change. The resulting RIP variable for the genotype (RIP-G) of interest will be uncorrelated to the conditioning variables and codes for the gene effect on facial morphology independent of the effects of sex and ancestry.

### *1.3 Statistical significance of effects on facial morphology using RIP variables*

Facial morphology coded using principal component projections of spatially-dense symmetrized quasi-landmark configurations implies a multivariate variable. As such, testing for association with sex, genomic ancestry, and genotypes, requires multivariate statistical techniques. However, the RIP variable is a supervised recoding of a multivariate response into a simpler univariate variable. Indeed, a RIP-variable codes for the effect of a predictor onto a response while at the same time projecting the multivariate response values onto a single direction through the response-space. Due to the nested LOO structure of BRIM, the statistical significance for the association between predictors and responses can be indirectly tested using powerful univariate statistical techniques that are not as stringent in their assumptions compared to their multivariate analogues.

Testing the significance of effects of sex, genomic ancestry and genotype on facial morphology was done under permutation in an receiver operating characteristic (ROC) curve analysis, correlation analysis and ANOVA analysis respectively. Each of these analyses generated an observed test-statistic between the predictor and RIP values. Subsequently, RIP values were permuted and the test-statistic under permutation was compared against the observed value. This was repeated 10,000 times and the number of times the permuted values were bigger or equal to the observed values divided by the total number of permutations, generated a *p*-value. For the ROC analysis, the self-reported sex defined two classes and the RIP-S values are tested to see how well they could classify faces by sex. Here, the “Area-Under-The-Curve” (AUC) was used as the test-statistic. For the correlation analysis, genomic ancestry was tested against the RIP-A values with the correlation value used as test-statistic. For the ANOVA analysis, the genotype, coded as an additive model, defined three groups and the associated RIP-G distributions were tested for different means where the F-statistic was used as test-statistic. Additionally, pair-wise ANOVA analyses between all three groups were performed in a similar way.

### *1.4 Visualizing and analyzing effects on facial morphology using RIP variables*

*Effect and effect-size analysis:* Principal components analysis on the 7,150 quasi-landmarks coordinates across the set of 592 research participants results in a series of orthogonal PCs. The first 44 PCs explain 98% of the total facial variation in this set of faces. These 44 PCs are the responses variable matrix used to compute the RIP variables for sex, ancestry, and genotype. To visualize and analyze their effect on facial morphology, first quasi-landmark configurations of faces are reconstructed from the 44 PCs. Subsequently; these are directly regressed on the RIP variables using PLSR. The effect on a particular quasi-landmark is then measured as the magnitude or Euclidean distance of its displacement in 3D space. The effect-size or strength of the relationship is reported as the variance explained by the PLSR model (R^2^). The partial effects (one variable independent from the others in a multiple regression) are coded in the partial regression coefficients. The partial effect-sizes are reported as the partial R^2^ values obtained from a reduced regression model. This reduced model is the regression model for a single independent variable after statistically removing the effect of all the other independent variables onto both the single independent variable itself and the dependent variable [4]. Statistical significance of both multiple and partial effects are tested under permutation for multivariate regressions [5]. Here, the respective multiple and partial R^2^ values are used as test-statistics with 10,000 permutations. For significance of the partial effects, permutation is performed under the reduced model [4].

Localized effects and effect-sizes per quasi-landmark are visualized using heat maps, while localized significance per quasi-landmark is plotted as significance maps using binary colors coding for being significant (yellow) or not (green) according to a *p*-value<0.001 [6]. Additionally, shape transformations across the range of RIP values provide visual changes illustrating the effect on facial morphology. Consistent with the visualization of PC transformations in Fig 2A and 2B, two shape transformations are constructed from the average face in the direction of the regression-line at -X and +X times the standard deviation of the RIP values. Transformations for the RIP-G variables were scaled to -6 and +6 times the standard deviation to make the effects visually evident. The effects of sex and genomic ancestry as represented by the RIP-S and RIP-A variables, respectively, are shown in transformations that are -3 and +3 standard deviations from the mean.

*Facial characteristic analysis:* Facial characteristics are typically used in clinical and anthropological descriptions of faces (e.g., long face, wide mouth, flat mid-face, etc.). While the effect (r) and effect-size (r^2^) analysis illustrate which quasi-landmarks are being affected, these fail to usefully communicate how these are changing and what is happening to the face as a consequence. In order to illustrate the effects on facial morphology of changes is PC scores and RIP variables, we used a range of facial shape change parameters (FSCP) in Supporting material Section 3.1. The effect on these FSCPs was tested within the same regression framework under permutation. The FSCP was measured between the two shape transformations at -X and +X times the standard deviation of the RIP values and served as an observed test-statistic. Under each permutation of the RIP-values, the shape transformations at -X and +X times the standard deviation of the RIP values were created again and the FSCP under permutation was compared to the observed value. This process is repeated 10,000 times and the number of times the permuted values are greater than or equal to the observed values divided by the total number of permutations provides an empirical *p*-value for a one-sided test of the null that there is no effect on the FSCP. Positive (H1+) and negative (H1-) one-sided tests and two-sided tests are similarly calculated (H2). In almost all cases, the direction in which the change in facial characteristic between the two shape transformations is irrelevant, such that the absolute permuted FSCP values was compared to the absolute observed value, generating the *p-value* for the two-sided test (H2). Similar to above, for the significance of partial effects, permutation was performed under the reduced model [30].

## 2. Empirical Analysis of BRIM

The behavior of RIP variables and statistical power of BRIM was investigated using controlled experiments. Using genomic ancestry, an example is provided for the response-based predictor information recovery ability of BRIM using noise-injected predictors. Additionally using alternate AIMs subsets as well as skin pigmentation (a proxy for genomic ancestry) and alternate population samples the robustness of the estimated ancestry effect on facial morphology using BRIM is tested. Using self-reported sex, an example is provided for the response-based predictor information recovery ability of BRIM when predictors have been partly misclassified. Finally, using a candidate gene SNP genotype (rs13267109 in *FGFR1*), an example is provided showing the enhanced conditioning power of BRIM on ancestry and sex when analyzing genotypes.

### 2.1 Genomic Ancestry

#### 2.1.1 Experiment: Noise injection

*Experimental setup:*

- Step1: Both genomic ancestry ($A$) and self-reported sex ($S$) were used as predictors in a multiple BRIM analysis on facial shape. This generates two RIP variables one for sex (RIP-S) and one for ancestry (RIP-A).
- Step 2: *A* was injected with noise according to$A^{'}=A+c*\left( -1+2*rand \right)$, where $rand$ is a uniform random generator between 0 and 1. And $c$ is a noise magnifying constant. The correlation between $A^{'}$ and $A$ was used to represent the magnitude of the injected noise.
- Step 3: $A^{'}$ was used as input into a partial BRIM analysis with conditioning variable RIP-S using 5 iterations. This generated a new RIP variable: RIP-A’.
- Step 4: The correlation between RIP-A’ and RIP-A as well as the correlation between RIP-A’ and RIP-$A$ were measured to determine the information recovery. This was done for each of six iteration steps in the partial BRIM analysis.

The magnifying constant $c$ ranged from 0 to 2 in steps of 0.1, resulting in 21 different levels of injected noise. For each level of noise injection the experiment was repeated 20 times and the average correlation values are reported along with other summary statistics and presented in box plot format.

*Results:* The correlation between $A^{'}$ and $A$ in function of the magnifying constant $c$ is depicted in Figure S4. It can be seen that with an increase of noise magnification the correlation drops as expected. The correlations of RIP-A’ in each iteration and for each level of noise with $A$ and RIP-A are shown in Figure S5 and Figure S6, respectively.

Finally, the correlations of A’ and RIP-A’ (after three iterations) with A and RIP-A are plotted in Fig S7 and Fig S8. It can be seen that the BRIM is able to recover information from the response variable improving a noisy predictor variable to an extent that the correlations with the original variables vastly improves. This even to the extent that a noisy variable (A’) with only 0.5 correlation with the original variable (A) results in a RIP-variable (RIP-A’) showing about 0.75-0.8 correlation with the original predictor variable (A) and 0.9-1.0 correlation with RIP-A.

Two conclusions can be drawn from these results. 1) The BRIM analysis is able to recover information in the presence of noise injected into the predictor variable values. The level of noise injected in this example (facial response variables and individual genomic ancestry) that can be tolerated by the system is quite high: Noisy variables that show correlations as low as 0.5 with the original predictor variables produce acceptable results (resulting in a correlation of > 0.9 against RIP-A). 2) BRIM converges rapidly in this example. The main improvement is gained in the first iteration and no more than three iterations are required for this particular variable (RIP-A) in these conditions. It is important to recognize that the ideal performance parameters of BRIM will likely vary from dataset to dataset and further research will be required to understand its functions.

#### 2.1.2 Experiment: Alternate AIMs subsets and population samples

*Experimental Setup:* Alternate AIMs subsets and population samples were used to test the robustness of the estimated RIP variables. A total of 176 AIMs were assayed in the United States and Brazilian participants, and a common core set of 68 AIMs were assayed in all participants. We first tested the effect of the number of AIMs used to estimate individual ancestry from DNA using the United States and Brazilian participants who were genotyped for the common panel of 176 AIMs. Various subsets of non-overlapping and overlapping AIMs (N=3, 15, 30, 50, 68, 77, and 176) were used as well as skin pigmentation as measured by the M-index were used in turn as initial predictor variables. Skin pigmentation was used as an initial predictor variable in these experiments as it is dependent on ancestry in West African/European population samples. Subsequently, a correlation matrix between all the predictor variables with varying precision and their resulting RIP variables was computed for each of the six iterations in the BRIM analysis. The results are depicted in Figure S9.

BRIM results in nearly identical RIP-A scores regardless of the size of the ancestry informative marker panel that is used to estimate genomic ancestry. The alternate AIMs panels do not only differ in the particular composition, but also in levels of ancestry information. That the AIMs panel with the least ancestry information (AIM panel 15) results in nearly identical RIP-A scores to the AIMs panel with the greatest ancestry information (AIM panel 176) illustrates the capacity of the BRIM method at recovering latent information in the covariance of facial traits and ancestry. The robustness of RIP-A estimates substantiates the generality of these models.

We next addressed the question of how the RIP variables depend on the population sample being analyzed. Using a common set of 68 AIMs, we estimated ancestry from DNA in the three populations with a dihybrid (West African/European) admixture model. RIP variables were estimated through the multidimensional face space for alternate sets of populations, namely, each population (United States, Brazil, and Cape Verde) alone, each of the three combinations of two populations, and then all three populations together. The same was also done for skin pigmentation as measured by the M-index. As above for the AIMs panel comparison, we computed correlation matrices. These matrices were computed for all RIP variables constructed from different population samples plus genomic ancestry estimated from 68 AIMS on the one hand and skin pigmentation on the other hand.

Figures S10 and S11 illustrate the correlation matrices over different population samples for genomic ancestry based on 68-AIMs and skin pigmentation, respectively. The lowest correlation is between the Cape Verdean and Brazilian population samples (r=0.70), the two population samples that are largely non-overlapping in their distributions of ancestry from DNA (see Figure 4A). These results illustrate the robustness of the RIP-A to the particular population used to model the ancestry/facial feature relationships. It is interesting to see that from the moment two populations are combined there are improvements. It may well be that there are significant differences in either the patterns of admixture stratification or the parental populations within or among these three countries and the differences here may be due more to biology than to analysis. Practically, one should include as large a sample of subjects as possible with the widest span on the genomic ancestry and population origins such that the most robust model can be produced. Additional work on comparisons across populations will be needed to clarify the extent to which regionally-specific models are needed. Likewise, experiments involving the derivation of RIP-A scores in different types of mixed population samples are required.

### 2.2 Self-reported Sex

#### 2.2.1 Experiment: Misclassification

*Experimental setup:*

- Step1: Both genomic ancestry ($A$) and self-reported sex ($S$) were used as predictors in a multiple BRIM analysis on facial shape. This generated two RIP variables one for sex (RIP-S) and one for ancestry (RIP-A).
- Step 2: A percentage$(p)$ of the self-reported sex values were inverted (1 becomes -1 and -1 becomes 1). This generated$S^{'}$. An ROC analysis was performed using S’^’^ as input variable and S as grouping variable and the area-under-the-curve (AUC) was reported to represent the magnitude of the misclassification error.
- Step 3: $S^{'}$ was used as input into a partial BRIM analysis with conditioning variable RIP-A using 6 iterations. This generated a new RIP variable: RIP-S’.
- Step 4: An ROC analysis was performed using RIP-S’^’^ as input variable and S as grouping variable and the area-under-the-curve (AUC) was reported. This was done for each iteration step in the partial BRIM analysis.

The percentage of misclassifications $p$ ranged from 0% to 70% in steps of 5%, resulting in 15 different levels of misclassification. For each level of misclassification the experiment was repeated 20 times and the average AUC values were reported.

*Results:* The AUC between $S^{'}$ and $S$ as a function of the percentage of misclassifications is depicted in Figure S12. It can be seen that increasing the percentage of observations that are misclassified reduced the AUC to 0.5 (which is equal to a classification by chance only) and then increases when more than 50% of the observations are misclassified, as expected. Misclassifying more than 50% results simply in re-coding a dichotomous variable like sex. The ROC results of RIP-S’ in each iteration and for each level of misclassification with $S$ is shown in Figure S13.

Finally, The ROC analysis after three iterations is depicted in Figure S14. It can be seen that BRIM is able to recover a substantial amount of the information that is lost through the misclassification the predictor. Much of the predictor information can be recovered: the noisy variable to an extent that the classification with the original variables vastly improves and this up to 25% of misclassifications.

Three conclusions can be drawn from these results. 1) The BRIM analysis is able to recover information in the presence of misclassification errors. A rate of up to 30% misclassification is tolerated with an acceptable result (namely the AUC drops to 95% of it’s maximum value at this point). For example, 177 observations out of the 592 observations in this sample were misclassified for sex and only 18 faces were not categorized correctly by RIP-S. 2) BRIM estimation of RIP-S in the context of facial response variables converges fast. The main improvement is already gained in the first iteration and no more than three iterations are required. 3) When more than 50% of the observations are misclassified, BRIM will start to correct the ones that were not misclassified, such that a consistent re-coding of all observations results.

### 2.3 Genotypes

#### 2.3.1 Experiment: Conditioning on genomic ancestry

*Experimental setup:*

- Step1: Both genomic ancestry (A) and self-reported sex (S) were used as predictors in a multiple BRIM analysis on facial morphology. This generated two RIP variables one for sex (RIP-S) and one for ancestry (RIP).
- Step 2: For each available genetic marker a partial BRIM analysis on facial morphology was performed **conditioned only on RIP-S**. For each bootstrap iteration the gene coding (G (additive model; AA=1, AB=0 and BB=-1) and subsequent RIP-G values) were tested for correlation with A and RIP-A.
- Step 3: For each available genetic marker a partial BRIM analysis on facial shape was performed **conditioned on** **RIP-S and A**. For each bootstrap iteration the gene coding (G (additive model; AA=1, AB=0 and BB=-1) and subsequent RIP-G values) were tested for correlation with A and RIP-A.
- Step 4: For each available genetic marker a partial BRIM analysis on facial morphology was performed **conditioned on** **RIP-S and RIP-A**. For each bootstrap iteration the gene coding (G (additive model; AA=1, AB=0 and BB=-1) and subsequent RIP-G values) were tested for correlation with A and RIP-A.

*Results:* The correlation results of the individual 144 RIP-G variables with A and RIP-A, after each iteration, without conditioning on ancestry are depicted using boxplots in Figures S15 and S16. It can be seen that there is a significant correlation between the original genotype G (Iter 0) and both A and RIP-A. It is also seen that without conditioning on ancestry in effect BRIM is transforming the initial predictor genotype variables (G) into RIP-G variables that are even more correlated with genomic ancestry effectively making them estimates of facial ancestry.

The correlation results of the individual 144 RIP-G variables (for each of the bootstrap iterations including genomic ancestry (A) as a conditioning variable) with A and RIP-A are depicted in Figures S17 and S18, respectively. It can be seen that after each iteration the correlation between RIP-G and both A and RIP-A becomes negligible. This implies that the facial effect measured by RIP-G variables in later iterations is largely independent from ancestry as required for valid genotype/phenotype association analysis. We also see that the effectiveness of conditioning to remove confounding improves with increasing bootstrap iterations. In this particular situation, the RIP-G estimates appear to stabilize or converge by about the fourth bootstrap iteration.

The correlation results of the individual 144 RIP-G variables (for each of the bootstrap iterations including the previously estimated RIP-A as a conditioning variable) with A and RIP-A are depicted in Figures S19 and S20, respectively. As previously it can be seen that after each bootstrap iteration the correlation between RIP-G and both A and RIP-A decreases. However it is also notable that this drop is achieved faster in the second iteration compared to conditioning on genomic ancestry, favoring RIP-A over A as conditioning variable.

Several conclusions can be drawn from these results. 1) Conditioning on individual genomic ancestry in an admixed population is required for traits that are differentially distributed between the parental populations, like facial features. Without such conditioning, the RIP-G variables derived from a BRIM will primarily model ancestral facial variation. 2) Bootstrap iterations are beneficial in reducing the correlation of these RIP-G variables with ancestral variables such as A and RIP-A: The conditioning effect improves over subsequent iterations. 3) The results of using A and RIP-A as conditioning variables are comparable. However, conditioning on RIP-A requires fewer iterations compared to conditioning on A, to reduce if not eliminate all ancestral facial variation from the measured RIP-G variables. Combined with the information recovery capabilities of RIP-A shown in experiments on genomic ancestry, we conclude that, compared to A, RIP-A is the preferred conditioning variable.

#### 2.3.2 Experiment: Conditioning on Self-reported Sex

*Experimental setup:*

- Step1: Both genomic ancestry (A) and self-reported sex (S) were used as predictors in a multiple BRIM analysis on facial morphology. This generates two RIP variables one for sex (RIP-S) and one for ancestry (RIP-A).
- Step 2: For each available genetic marker a partial BRIM analysis on facial morphology was performed **conditioned only on RIP-A**. For each bootstrap iteration the gene coding (G (additive model; AA=1, AB=0 and BB=-1) and subsequent RIP-G values) were tested for correlation with S and RIP-S.
- Step 3: For each available genetic marker a partial BRIM analysis on facial morphology was performed **conditioned on** **RIP-A and S**. For each bootstrap iteration the gene coding (G (additive model; AA=1, AB=0 and BB=-1) and subsequent RIP-G values) were tested for correlation with S and RIP-S. Note that S as a conditioning variable is not “brimmed” and does not change in the analysis.
- Step 4: For each available genetic marker a partial BRIM analysis on facial morphology was performed **conditioned on** **RIP-A and RIP-S**. For each bootstrap iteration the gene coding (G (additive model; AA=1, AB=0 and BB=-1) and subsequent RIP-G values) were tested for correlation with S and RIP-S.

*Results:* The correlation results of the individual 144 RIP-G variables with S and RIP-S, after each iteration, without conditioning on sex are depicted using boxplots in Figures S21 and S22 respectively. It can be seen that there is a correlation between the original genotypes G (Iter 0, a measure of the sex-information content of the G variable) and both S and RIP-S. It is also seen that without conditioning on sex in effect BRIM is transforming the initial predictor variable genotype (G) into RIP-G is to create response-based imputed variables that are even more highly correlated with sex.

The correlation results of the individual 144 RIP-G variables (for each of the bootstrap iterations including self-reported sex (A) as a conditioning variable) with S and RIP-S are depicted in Figure S23 and S24, respectively. It can be seen that after each iteration the correlation between RIP-G and both S and RIP-S drops to a situation where there is hardly any correlation left. This implies that the facial effect measured by RIP-G variables in later iterations is largely independent from sex as required for valid genotype/phenotype association analysis. It is also shown that the iterative improvements clearly increase the conditioning effect as claimed. In this particular situation, the RIP-G estimates appear to stabilize or converge by about the third bootstrap iteration.

The correlation results of the individual 144 RIP-G variables (for each of the bootstrap iterations including the previously estimated RIP-S as a conditioning variable) with S and RIP-S are depicted in Figures S25 and S26, respectively. As previously it can be seen that after each bootstrap iteration the correlation between RIP-G and both S and RIP-S drops to a situation where there is hardly any correlation left. However it is also notable that this drop is achieved faster and stronger compared to conditioning on self-reported sex, favoring RIP-S over S as conditioning variable.

Several conclusions can be drawn from these results. 1) Conditioning on sex is required for traits that are differentially distributed between the sexes, like facial features. When conditioning on genomic ancestry or RIP-A, but without conditioning on sex, the RIP-G variables derived from BRIM will primarily model sexual dimorphism in facial variation. 2) Bootstrap iterations are beneficial in reducing the correlation of these RIP-G variables with sex variables such as S and RIP-S: The conditioning effect improves over different iterations. 3) The results of using S and RIP-S as conditioning variables are comparable. However, conditioning on RIP-S requires fewer iterations and is stronger compared to conditioning on S, to reduce if not eliminate all sexual dimorphism in facial variation from the measured RIP-G variables. Combined with the information recovery capabilities of RIP-S shown in Supporting material Section 2.2.1, we conclude that, compared to S, RIP-S is the preferred conditioning variable.

#### 2.3.3 Experiment: Conditioning with traditional regression techniques

Here we illustrate the benefit of using RIP variables and the framework of BRIM in the context of modeling the effect of genes on facial morphology while conditioning on ancestry and sex. The SNP rs13267109 in *FGFR1*, a gene that showed a significant association with facial morphology in a normal range (Table S1). Since alleles at SNP rs13267109 are ancestry informative, they can also be shown to correlate with genomic ancestry. In this experiment we compare the effect of rs13267109 on facial morphology using four approaches highlighting why proper conditioning on ancestry is critically important.

*Experimental setup:*

1. A standard regression technique without conditioning on ancestry. The independent variables are, self-reported sex and genotypes for rs13267109 coded as an additive model (AA = 1, AB = 0, BB = -1). The comparable (to the current implementation of BRIM) standard technique used was a linear PLS regression.
2. A standard regression technique while conditioning on genomic ancestry. The independent variables are, self-reported sex, genomic ancestry estimated from 68 AIMS and rs13267109 genotypes modeled additively (AA = 1, AB = 0, BB = -1).
3. A standard regression technique while conditioning on facial ancestry. Facial ancestry is the RIP-A variable obtained using a BRIM analysis of genomic ancestry on facial morphology. The independent variables are, self-reported sex, facial ancestry (a RIP variable) and rs13267109 genotypes modeled additively (AA = 1, AB = 0, BB = -1).
4. BRIM while conditioning on facial ancestry and sex. This is the approach we propose. The independent variables are facial sex (a RIP variable), facial ancestry (a RIP variable) and rs13267109 genotypes modeled additively (AA = 1, AB = 0, BB = -1). The BRIM analysis will create a continuous RIP variable for rs13267109, and the effect of this variable is given as an output.

*Results:* The results of the effect of rs13267109 on facial morphology for all four approaches are illustrated in Figure S27. From left to right, approach 1 to 4 respectively. It is seen that: 1) without conditioning on ancestry, the effect of the gene is picking up ancestral facial differences comparable to Figure 3A, hence the need to condition on ancestry. 2) By conditioning on genomic ancestry using BRIM, we observe residual variation in the lips, chin and nose that is consistent with ancestral differences in facial shape. 3) By conditioning on the RIP variable coding for facial-ancestry instead of genomic-ancestry, these residual variations are downscaled. This illustrates the advantage of using a RIP-A for ancestry as conditioning variable. 4) Using the complete BRIM framework the residual ancestral variations are eliminated completely to an extent that the true effect of rs13267109 independent from ancestry is obtained. This illustrates the advantage of recoding rs13267109 by a RIP variable using BRIM in which iterations allow to improve the conditioning of covariates, as demonstrated in the previous experiments.

## 3. Facial Characteristics

### *3.1 Facial Shape Change Parameters*

Given two particular faces, such as two shape transformations at opposite sides of the range of RIP values, facial shape change parameters (FSCPs) are either obtained as the difference/ratio between measured features on both facial shapes or as a directed change from one facial shape to the other. Features and/or directed changes can be defined on the level of quasi-landmarks as well as on the level of specific facial regions, which are regionally defined subgroups of quasi-landmarks (Figure S28). The following categories of shape features and directed changes were used:

- *Curvature*: The signed mean curvature in each quasi-landmark is used where a negative and positive sign indicate a concave and convex local shape, respectively. A curvature of zero indicates a locally flat shape. On the level of a facial region, the average of all signed mean curvatures of the quasi-landmarks within the facial region is taken. A curvature-based FSCP is obtained by taking the difference between corresponding curvature measurements on both facial shapes. These types of FSCPs provide insight whether or not facial shape is changing in aspects of flatness (concavity/convexity).
- *Area:* The average area of all polygons in which a quasi-landmark participates as a vertex is used to summarize the local area in each quasi-landmark. On the level of a facial region, the sum of all polygon areas within that facial region is used to summarize the area for the region. An area-based FSCP is obtained by taking the negative log ratio between corresponding area measurements on both facial shapes. These types of FSCPs provide insight whether or not facial shape is changing in aspects of changes in the local surface area between the two reference faces.
- *Directed Displacements:* The directed displacement is measured as the signed magnitude of the positional change of a quasi-landmark in space from the first facial shape to the second facial shape. The displacement is measured in reference to four directions as listed below. These types of FSCPs provide summaries of how face shape is changing with respect to particular spatial directions and a variety of different directions can be defined including:
  - *Normal direction:* Here the displacement is projected onto the direction of the normal plane through the quasi-landmark in the first facial shape. It provides insight whether or not facial shape is locally changing inwards or outwards.
  - *Vertical direction:* Here the displacement is projected onto the vertical principal axis of the average face, against which all shape transformations are aligned. It indicates whether or not facial shape is changing upwards or downwards along the longitudinal or coronal axis (in anatomical terms superiorly or inferiorly, respectively). Note that for these and the following two computations the three principal axes of the average face are aligned with the X, Y and Z axis of the 3D Euclidean space. As such the vertical direction coincides with the Y axis.
  - *Horizontal direction:* Here the displacement is projected onto the horizontal principal axis (X axis) of the average face. It indicates whether or not facial shape is changing bilaterally towards the left or right along the horizontal or transverse axis (in anatomical terms medially or laterally would describe these positions).
  - *Depth direction:* Here the displacement is projected onto the depth principal axis (Z axis) of the average face. It shows how facial shape is changing along the sagittal axis (in anatomical terms anteriorly and posteriorly, respectively).

On the level of facial region, the average of the displacements of the quasi-landmarks within a facial region is taken.

- *Conventional morphometric features (CMF):* A variety of conventional morphometric features, such as distances and angles between anatomical landmarks exist in the literature. A CMF-based FSCP is obtained by taking the difference or ratio between corresponding CMF measurements on the two facial shapes. These measures provide insight whether or not facial shape is changing in a variety of aspects. Note that, anatomical landmarks in contrast to quasi-landmarks are typically indicated manually. However, manual indication of anatomical landmarks is prone to operator error and is also impractical in the permutation framework used. Therefore, placement of such landmarks was automated in the following way: Anatomical landmarks (Figure S29) were first manually indicated onto 24 individual faces with homologous quasi-landmark configurations. After indication the anatomical landmarks are expressed as a function of the quasi-landmarks using barycentric coordinates. This allows the mapping of the anatomical landmarks from each of the individual configurations to any other facial quasi-landmark configuration. To incorporate indication error, the indication of the anatomical landmarks onto the average facial shape was done by three observers, generating a distribution of 72 measurements per CMF. The average measurement per CMF was subsequently used.

Curvature changes, area changes, and normal directed displacements are summarized per quasi-landmark and are visualized using heat maps. Positive (H1+) and negative (H1-) one-sided tests as well as the two-sided tests (H2) per quasi-landmark are plotted as significance maps using binary colors: Quasi-landmarks showing statistically significant (*p*-value<0.001) FSCP are colored yellow and non-significant quasi-landmarks are colored green (Figures S33-S44 below).

Using the measurement machinery as presented in this section the following list of additional FSCPs in Table S1 were defined. It should be noted that some facial characteristics or traits were straightforward to measure such as mouth width. However, more subjective, descriptive, and complex facial characteristics such as cleft lip, frontal bossing (a trait that involves relative changes in different parts of the upper face) and flat midface (a trait that involves both relative changes in different parts of the face and can result from different relative changes), is more challenging. Here often multiple measurements have been defined measuring different aspects of the same facial characteristic. However, the measurement of these remains an oversimplification.

4. Extended Results: Sex, Ancestry, and Gene Effects

### 4.1 Significant effects on facial morphology

The reporter operating characteristic (ROC) curve and permuted null distribution for the effect of self-reported sex on facial morphology are depicted in Figure S32 and show an observed AUC=0.994 with a permuted *p*-value<0.0001, which indicates a strong effect of sex on facial morphology as expected. It also means that based on the resulting RIP-S values, 588 out of the 592 individuals were classified correctly.

The correlation analysis between genomic ancestry and RIP-A shows a correlation r=0.8 with a permuted *p*-value=0 and similar to results obtained with sex, this indicates highly significant relationship between genomic ancestry and facial ancestry (RIP-A). For each SNP tested we calculated a RIP-G variable using BRIM conditioning the RIP-Gs for the effects of RIP-A and RIP-S to create a valid model. These RIP-G values were tested for significant differences among genotype categories using ANOVA (Table S2). Several SNPs show significant effects on facial morphology in this sample. SNP selection involved three factors, 1) The SNPs typed are ancestry informative markers (AIMs) which were located in genes which are associated with craniofacial dysmorphologies (or animal model effects), and 3) show patterns of accelerated evolution in either European or African populations. It is reasonable to propose that they might affect normal-range craniofacial variation to an extent as well. The three-group ANOVA conditioning on RIP-A and RIP-S gave 24 SNPs (shown yellow font in Table S2) using the significance level ($\alpha$) of 0.1, about double the traditional level, 0.05. The effects of these 24 candidate genes in conjunction with sex and genomic ancestry are analyzed and visualized in depth in the next section.

4.2 Visualization and Analysis of effects on facial morphology

#### 4.2.1 Effect and effect-sizes

The effect-size and statistical significance per quasi-landmark along with alternate shape transformations for sex and ancestry are depicted in Figure S33. The effects of sex observed here are consistent with the effects found in a recent study on sexual dimorphism in facial symmetry [5] and are primarily on the supraorbital ridges, nose, cheeks, mandible, and midface. The effects on the West African/European axis of ancestry mainly involve changes in the nose, lips, chin, mandible and supraorbital ridges.

Sex and genomic ancestry have clear effects on facial morphology, the results of which are interesting for a variety of reasons. Foremost among these is the fact most people are quite familiar with the facial effects of these variables. Given that quasi-landmark remapping, PCA, and BRIM are abstract and relatively complex statistical methods, it is encouraging to observe familiar results for variables like sex and ancestry. Observing the shape transformations in Figure S33 for example, one can clearly recognize which faces result from transformations in the male, female, European and African RIP variable directions. It is notable that the perception study experiments described above support more formally the concordance between RIP-A and RIP-S variables perceptions of facial ancestry and facial sex.

The effect, effect-size and statistical significance per quasi-landmark along with alternate shape transformations for the 24 candidate-gene SNPs are shown in Fig S34and Fig S35. A variety of effects, often highly localized in different parts of the face, are seen throughout these results. In some genes, multiple SNPs in the same gene show significant effect on facial morphology and these typically show a similar effect pattern, for example, DNMT3Bb and c as well as SATB2b, c, d, and e. The maximum value of the effect-size is dependent on the SNP. Exact values of this maximum and the distribution of the effect-size over the quasi-landmarks as well as an overall partial effect-size (all quasi-landmarks combined) can be found in Table S3. In essence the overall partial effect size is the amount of facial variation coded in all quasi-landmarks that is explained by a RIP-G, independent of Sex and Ancestry.

#### 4.2.2 Facial characteristics

The effects of sex and ancestry in terms of area, curvature and normal displacement on the level of quasi-landmarks are shown in Figure S36, S37, and S38.

Facial regions primarily affected by sex include the midface, chin, nose and supraorbital ridges, which are very similar to patterns of facial sexual dimorphism recently reported [5]. In that study the same type of 3D facial images and phenotyping was used. However, a more traditional geometric morphometric approach, in contrast to BRIM, was used. Using the facial shape change parameters (FSCPs) described in this work, additional insights into the sexual dimorphism of the face can be made. Males exhibit a larger nose, chin, mandible, upper lip, philtrum, inner upper canthic region, and supraorbital ridges, while having a smaller midface and smaller eyes in terms of surface area. Curvature differences mainly occur in the orbital regions and around the mouth, with the nasal bridge and supraorbital ridges standing out as showing the most significant differences in local curvature. An outward movement of the entire nose, chin, supraorbital ridges, and philtrum and an inward movement of the cheekbones, cheeks and eyes are seen moving from the female to male transformed face. The local FSCP defined in Table S1 show patterns of sex effect that include characteristic changes throughout the face (see Table S4).

Facial regions primarily affected by ancestry include the chin, mandible, lips, nose and supraorbital ridges. In terms of surface area, the European transformed face shows larger paranasal tissues and inner canthic regions, and a larger midface and chin. The European transformed face also shows smaller lips, philtrum, alae nari and nares as well as a smaller central forehead and smaller eyes. The main curvature differences are located at the nasal bridge, supraorbital ridges, columella, philtrum, and chin all of which show greater convexity in the European transformation than in the African transformation. An outward movement of the nasal bridge, nasal ridge, supraorbital ridges, chin, and mandible and an inward movement of the alae nari, lips, perioral region, cheeks, and orbital regions and are seen moving from the African to the European transformed face. Similar to sex, a range of characteristic changes throughout the face are seen in local FSCP summaries (Table S4).

Some of the facial characteristics in Table S4 affected by sex and ancestry are not directly associated with the regions affected by sex and ancestry as shown in Figure S37. For example the thickness of the lips is affected by sex as noted in Table S4. This is highly due to the fact that the face is a multipartite phenotype consisting of connected facial regions or modules that interact with each other. Hence changes in certain facial regions, will inevitably affect aspects of neighboring regions or other regions even in more distant parts of the face. For example, it has recently been shown that asymmetry in the lower face introduces a counteracting asymmetry in the upper face [7]. Furthermore, as noted previously, the FSCPs listed in Table S1 are often oversimplifying measurements that are seen to be easily affected in a variety of ways. The interaction between different facial regions is also supported by the manner in which the face was phenotyped and analyzed. Both PCA and PLSR focus on the covariance structure of the quasi-landmarks. In the case of PCA this results in principal components coding for facial shape variations in which facial shape as a whole varies in harmony. From a technical point of view this can be seen as a global shape model in contrast to local shape models [8]. In the case of PLSR, the covariance structure of the quasi-landmarks leads to model stabilization, which is required when the number of observations (faces) is smaller than the number of highly correlated dependent variables (quasi-landmarks).

The effects of the 24 candidate genes in terms of area ratio, curvature difference, and normal displacement on the level of quasi-landmarks are shown in Figures S39 and S40, Figures S41 and S42, and Figures S43 and S44, respectively.

#### 4.2.3 Comparing and contrasting facial changes in the clinical and normal range

We carefully examined the RIP-G transformations and FSCP results for the suggestive SNPs (*p*<0.1) in the ANOVA test. Some striking correspondence with clinical dysmorphology reported in the human syndromes associated with mutation of the respective genes or with relevant animal models is observed. In the context of these observations, below we review the results of the analysis the 24 candidate genes in the order of increasing p-value as shown in Table S2 (note that when facial characteristic changes or effects are mentioned or noted, we refer to statistically significant effects and FSCPs):

- Mutations in the human RNA polymerase I subunit D (***POLR1D***; OMIM#613715) gene on chromosome 13q12.2 can lead to the autosomal dominant condition Treacher-Collins syndrome-2 (TCS2; OMIM#613717). The facial phenotype in TCS2 includes a distinctive pattern of facial bone hypoplasia associated with bilateral downward slanting palpebral fissures and symmetric convex facial profile resulting from hypoplasia of the zygomatic bones. Affected persons may also manifest colobomas of the lower eyelids, and mandibular hypoplasia.
- The normal-range results of the SNP in rs507217 in *POLR1D* depicted in Fig S39 indicate strong effects in the eyes as well as the forehead and mandible. When observing the shape transformations associated with this SNP, downward slanting palpebral fissures can be perceived in shape transformation “B”. The curvature and the normal displacement of the eyes are affected in Fig S41 and Fig S43, respectively and many local FSCPs related to the eyes including downward slanted palpebral fissures are noted in Table S4. The bilateral parts of the mandible are affected in Fig S39 and this mainly in terms of area (Fig S39) and normal displacement (Fig S43). Finally, the cheekbones are significantly different in terms of area (Fig S39), which also results in associated changes like malar flattening and midface retrusion (Table S4). It is highly possible that these characteristics are associated with differences in zygomatic bone development.
- Genomic deletions of chromosome 5p15.2, which can include the human delta-catenin 2 (***CTNN2D***; OMIM#604275), result in Cri-du-chat syndrome. The craniofacial features of Cri-du-chat syndrome include a round face, hypertelorism, a very wide nasal bridge, downward slanting palpebral fissures, a wide mouth, down-turned corners of the mouth, micrognathia and epicanthal folds.
- The normal-range effects of the SNP in rs2277054 in *CTNN2D* shown in Figure S39 are found in the midface, nose, eyes (with an emphasis on the epicanthic region), lower mandible and forehead. Wide nasal bridge and orbital hypertelorism can be perceived in the shape transformations and also Table S4 (eyes widely spaced). The nose in general appears to be different in width in each of the three primary FSCPs as well as in the local FSCPs like narrow nasal ridge, wide nose and wide nasal bridge as listed in Table S4. Other nasal features in Table S4 are affected as well. The curvature of the nasal ridge (Fig S41) and the normal displacement of almost the entire nose region (Fig S43) are significantly different. Consistent with the results in Table S4, the normal displacement results indicate a nose that is more prominent in the anterior-posterior plane and wider versus a narrower and more retruded nose. The area of the nasal bridge and the region above it are affected (Fig S39). The shape transformation “B” (Fig S39) appears to be rounder and a difference in facial roundness is noted in Table S4. The chin is more prominent in shape transformation “A” compared to “B” and only one out of the three FSCPs for micrognathia appears to be significant. However, this particular FSCP, measures the normal displacement of the chin region, which is confirmed in Fig S43. It is interesting to note that there is a change in the area FSCP for the entire midface and cheek region as well as the chin. This area change might underlie our perception of a prominent (forwardly placed) versus less prominent (inwardly placed) chin, which might illustrate that apparent facial characteristics are modulated by their local morphological context. This same change in the area of the mandible and cheeks, might promote the perception of a wider mouth, however the mouth itself is only slightly affected in terms of area, curvature, and normal displacement and no change in mouth width was noted in Table S4. Hence we may not conclude that the normal-range results include an affected mouth width.
- Mutations in the human semaphorin 3E (***SEMA3E***) gene (OMIM# 608166) located on 7q21.11 are associated with CHARGE syndrome (OMIM# 214800). The facial features associated with this condition include: a square face with a broad and prominent forehead, a prominent nasal bridge and columella, a flat midface, cleft lip and/or palate and facial asymmetry.
- The normal-range results of the SNP rs2709922 in *SEMA3E* depicted in Fig S39 indicate effects in the lower orbits, midface, nose, nostrils, philtrum, the mandible, lower lip and chin. The shape transformations indicate a change in overall facial shape, and changes in facial squareness/roundness are noted in Table S4. Although the forehead is not affected in Fig S41, a broad/narrow forehead as well as changes in head circumference (microcephaly) are noted in Table S4. This may be due to the area changes in the metopic ridge (Fig S39) and the normal displacements of the forehead Fig S43). Both the nasal bridge and nasal ridge in shape transformation “B” appear to be wider, which is confirmed in Table S4. Perceptually the midface is different between the two shape transformations. However, area changes (Fig S39), curvature changes (Fig S41) and normal displacement changes (Fig S43) are only noted in small some regions of the midface. It is interesting to note that regions adjacent to the midface, especially the philtrum and upper lip, are affected in terms of area, curvature, and normal displacement. A relative interplay between facial regions might be consistent with the noted malar flattening and midface retrusion in Table S4. Furthermore, the palate, philtrum, and upper lip are typical regions affected by cleft lip and palate, and the activity within these regions is confirmed in Table S4, for half of the cleft lip related FSCPs. Furthermore, the thickness of the lips, the width of the mouth and the length of the philtrum are also noted in Table S4. Due to the fact that only the symmetry component of faces was modeled in this work, the normal range effects are not able to reflect any asymmetry related facial characteristics.
- The gene solute carrier family 35 member D1 gene (***SLC35D1***; OMIM#610804) is located on human chromosome 1p31.3. Mutations in *SLC35D1* have been shown to result in Schneckenbecken dysplasia (OMIM#269250) which has a characteristic facial feature of “superiorly oriented orbits”.
- The normal-range results of the SNP in rs1074265 in *SLC35D1* depicted in Fig S39 indicate strong effects at the eyes and orbital regions, as well as the midface and the chin. In accordance with classic phenotypic descriptions of superiorly oriented orbits, one can readily perceivea difference in the orientation of the eyes and orbits between the shape transformations, the eyes appear to be looking downwards “A” or upwards “B”. Dividing the eyes and orbits into upper and lower regions, we see opposite changes in terms of area (Fig S39), curvature (Fig S41) and normal displacement (Fig S43). Additionally, the results of the local FSCPs measuring superiorly oriented orbits (Table S4) are consistent with this facial characteristic . The effects in the midface and the chin are mainly changes in terms of curvature (Figure S41) and normal displacement (Figure S43 leading to malar flattening and along versus short face (Table S4).
- Mutations in the human fibroblast growth factor receptor 1 (***FGFR1***;OMIM#136350) gene located on chromosome 8p21.23-p21.22 can result in four autosomal dominant craniofacial disorders: Jackson-Weiss syndrome (OMIM#123150), which is characterized by craniosynostosis and midfacial hypoplasia; trigonocephaly (OMIM#190440), which is characterized by a keel-shaped forehead resulting in a triangle-shaped cranium when viewed from above; osteoglophonic dysplasia (OMIM#166250), which is characterized by craniosynostosis, a prominent supraorbital ridge, a depressed nasal bridge; and Pfeiffer syndrome (OMIM#101600), which is characterized by midface hypoplasia, and depending on the subtype, ocular proptosis, a short cranial base, and a cloverleaf skull.
- The normal-range results of the SNP rs13267109 in *FGFR1* depicted in Fig S39 indicate the strongest effects in the supraorbital ridges, the forehead, the eyes, midface, nose and the corners of the mouth. It should be noted that most of the face is significantly affected. Perceptually, the strongest differences in the shape transformations are indeed the forehead, supraorbital ridges and nasal bridge. Area changes (Fig S41) occur in the forehead, nasal tip, nasal bridge/root, midface, cheeks and the chin. The curvature changes (Fig S41) are located in the supraorbital ridges, with opposite changes on the forehead slightly above them (indicating prominent supraorbital ridges), and in the nasal bridge and inferior half of the eyes, with opposite changes in the cheekbones (indicating midface hypoplasia). Normal displacements (Fig S43) occur in the forehead, supraorbital ridges, nasal tip, paranasal tissues and cheeks. Focusing on the forehead as one of the most prominent changing regions, noted related FSCPs in Table S4 include microcephaly, frontal bossing, prominent metopic ridge, forehead short/long, forehead broad/narrow, and forehead sloping. Supraorbital ridges under/overdeveloped is also noted in Table S4. With regard to the midface, FSCPs noted in Table S4 include malar flattening, midface retrusion/flat midface (midfacial hypolplasia) and prominent maxilla. Finally for the nose, noted FSCPs in Table S4 include wide nasal bridge, wide nose, large nasal tip and retruded nasal ridge. Although area and curvature changes clearly occur in the nasal bridge and affect its appearance, the FSCP for depressed nasal bridge is not noted in Table S4, because there was no normal displacement measured in this region (Fig S43).
- Mutations in the human WNT 3 protein, which is encoded by the ***WNT3*** gene (OMIM#165330) located on chromosome 17q21.31, can result in an autosomal recessive condition, Tetra-Amelia syndrome (OMIM#273395). Infants with Tetra-Amelia are generally stillborn or die as neonates. In addition to having no limbs or pelvis, they have many other anatomical problems including numerous craniofacial anomalies: cleft lip/cleft palate, micrognathia, microtia, single naris, prominent nose, no nose, microphthalmia, microcornea, coloboma, and palpebral fusion. Note that many of these features are associated with the eyes.
- The normal-range results of the SNP rs199501 in *WNT3* shown in Fig S39 indicate effects in the eyes, forehead towards the nasal bridge, philtrum, lips, and chin. The shape transformations are clearly distinct with several characteristic facial changes. The eyes, similar to the results of *SLC35D1*, show opposite changes in area (Fig S39), curvature (Fig S41), and normal displacement (Fig S43) for the upper and lower parts of the eyes, leading to a wide range of FSCPs noted in Table S4 that are related to the eyes, such as superiorly oriented orbits, shallow orbits, palpebral fissures downslanted, eyes large and eyes widely spaced. Similar to *SEMA3E*, the philtrum and upper lip are typical regions affected by cleft lip and palate, and a strong change in terms in area (Fig S39), curvature (Fig S41), and normal displacement (Fig S43) is observed within these regions for the normal-range results. Half of the cleft-lip related FSCPs are significant (Table S4). The thickness of the lips, the width of the mouth, and the length of the philtrum are also noted in Table S4. The chin exhibits changes in area (Fig S39) and curvature (Fig S41) as well as normal displacement (Fig S43) all three FSCPs for micrognathia are significant (Table S4). The entirety of the of the nose, through the nasal bridge and toward the inferior limit of the forehead is affected and some nose related FSCPs are noted in Table S4, such as width of the nasal ridge and bridge, snubbed nose and anteverted nares.
- The mouse homologue of the human low density lipoprotein receptor-related protein 6 (***LRP6;*** OMIM#603507) gene is critical for mouse lip development and bilateral cleft lip is seen in *LRP6* knockout mice (*26*). LRP6 is known to interact with the WNT signaling pathway. However, no human craniofacial diseases have yet been linked to the *LRP6* gene or to the gene region on human chromosome 12p13.2.
- Observing the shape transformation in Fig S39, a change from prominent lips with a thick and convex vermillion to less prominent lips with a thin and more concave vermillion. This is confirmed by looking at the normal displacement results (Fig S43). Interestingly, the lips appear to be perfectly segmented out in the H_1_- significant map (Fig S43). Besides normal displacements, some curvature changes in the lips are observed (Fig S41) and area changes in the regions surrounding the lips (Fig S39). All but one cleft lip related, and several nose and eye/orbit related FSCPs are significant (Table S4).
- Mutations in the human special AT-rich sequence binding protein 2 gene (***SATB2***; OMIM#608148) located on 2q33.1 can result in cleft palate with mental retardation (OMIM#119540). Craniofacial features of deletions of the *SATB2* gene include prominent forehead, prominent nasal bridge, wide columella, micrognanthia, microcephaly, and cleft palate [9].
- Statistically significant normal-range effects of the four (rs1357582, rs6759018, rs4530349, and rs4673339) of the five SNPs tested in *SATB2* are depicted in Fig S39 (rs1357582) and Fig S40 (rs6759018, rs4530349, and rs4673339). The effects, FSCP results, and the shape transformations of the different SNPs in *SATB2* are very similar. In the shape transformations, the shape of the nose as well as the chin and overall head and forehead are distinctively different. All four SNPs show frontal bossing, a prominent metopic ridge, forehead sloping and forehead width change (Table S4). Two out of four SNPs also exhibit a change in forehead length. Related to the forehead, all four SNPs are significant for the microcephaly FSCP. All four SNPs also show area and (Figs S39-S44) curvature changes (Figs S41-S42) as well as normal displacements (Figs S43-S44) in at least some part of the forehead. All four SNPs show curvature changes (Figs S41-S42) and normal displacements (Figs S43-S44) of the nasal ridge and bridge, and all but rs4673339, also show area changes (Figs S39-S40) in these regions. The FSCPs related to the nose (Table S4), including nasal ridge narrow and retruded, wide nasal bridge, snubbed nose, and anteverted nares, are also significant for all four SNPs. Some SNPs also show a large nasal tip and a wide nose, concluding that the nose is clearly affected by this gene. The chin is alternatively affected in terms of area, curvature, and normal displacement depending on the SNP analyzed, which is also seen in Table S4 where different FSCPs for microganthia are noted across the four SNPs with some overlap present. There are changes of the curvature of the philtrumfor all SNPs and for some SNPs the normal displacement and area are altered as well. For all SNPs most of the cleft lip and palate related FSCPs are significant.
- Mutations in the human ***EVC2*** gene (OMIM#607261) located on chromosome 4p16.2 can lead to the autosomal recessive condition known as Ellis-van Creveld syndrome (OMIM#225500), which is characterized craniofacially a “partial hare-lip” (short upper lip) or “lip-tie” (upper lip frenulum). Mutations in *EVC2* can also lead to an autosomal dominant disorder called Weyers acrofacial dysostosis (OMIM#[193530](http://www.omim.org/entry/193530)), which has some facial phenotypic overlap.
- The normal-range results of the SNP rs1001971 in *EVC2* shown in Fig S39 indicate effects in the alae nasi, cheekbones and lateral orbits, affecting the upper lip and philtrum, nose, orbits and the forehead and the lower chin. Perceptually, the strongest difference is indeed located in the lower nose area, philtrum and upper lip, and additionally an overall long/ short face difference is noted. Also notable are area changes is the region around the lips. In fact the lips appear to be delineated in the H1- significance map (Fig S39). The most prominent curvature changes occur in the orbits, cheekbones and chin. An interesting opposite change in curvature is noted in the lower and upper part of the upper lip. The normal displacement (Fig S43) is clearly noted in the eyes, cheekbones, forehead, nares, columella, and lower chin. A number of the FSCPs related to the orbits and forehead are noted in Table S4. The same is true for the nose. Regarding lip variation, half of the cleft lip and palate FSCPs are noted; thickness of the lips, mouth width, and a borderline (*p*=0.052) change in philtrum length are observed.
- Deletions of human chromosome 17p11.2 and point mutations in the gene ***RAI1*** can cause Smith-Magenis Syndrome (SMS; OMIM#182290). The facial characteristics are perhaps best summarized by [10]:

*“The facial phenotype of SMS is quite distinctive, even in the young child. The overall face shape is broad and square. The brows are heavy, with excessive lateral extension of the eyebrows. The eyes slant upwards and appear close set and deep set. The nose has a depressed root and, in the young child, a scooped bridge. With time, the bridge becomes more ski jump shaped. The height of the nose is markedly reduced while the nasal base is broad and the tip of the nose is full. The shape of the mouth and upper lip are most distinctive. The mouth is wide with full upper and lower lips. The central portion of the upper lip is fleshy and everted with bulky philtral pillars, producing a tented appearance that, in profile, is striking. With age, mandibular growth is greater than average and exceeds that of the maxilla. This leads to increased jaw width and protrusion and marked midface hypoplasia.”*

- The normal-range results of the SNP rs4925108 in *RAI1* depicted in Fig S39 indicate effects in the nasal tip and nasal bridge, eyes, midface, cheeks, chin, and the top of the forehead. The shape transformations both frontal (Fig S41) and lateral (Fig S39) are quite distinct and the most remarkable perceived differences include a ski jump shaped nose (shape transformation “A”) with bulky philtral pillars/ridges. The eyes exhibit small area (Fig S39) and curvature changes (Fig S41) and more substantial normal displacement changes (Fig S43). The FSCP for palpebral fissures downslanted (being the opposite of slanting upwards) and proptosis (related to forward displacement of the eyes) are noted in Table S4. The effects in the nose are clearly interesting and distinct, especially the opposite normal displacement (Fig S43) of the nasal bridge and the area slightly above the nasal tip, creating the appearance of an upturned nasal tip. Significant nose-related FSCPs in Table S4 include, nasal bridge depressed and nasal bridge/ridge width (one out of two measures), nose snubbed, and nares anteverted. The shape of the lips and mouth differ in terms of curvature (Fig S41) and in terms of area (Fig S39). Related FSCPs noted in Table S4 include thickness of the lips and the length of the philtrum. The midface and the cheeks are most substantially affected in terms of normal displacement (Fig S43), and are consistent with the significant local FSCPs in Table S4, such as malar flattening and midface retrusion/hypoplasia.
- Mutations in the human ***ADAMTS2*** (OMIM#604539) gene located on 5q35.3 can cause Ehlers-Danlos syndrome, type VIIC (OMIM#225410). The facial features of Ehlers-Danlos VIIC include epicanthal folds, a depressed nasal bridge, micrognathia, large eyes, a small chin, sunken cheeks, a thin nose and thin lips.
- The normal-range results of the SNP rs3822601 in *ADAMTS2* shown in Fig S39 indicate effects in midface, lower nose, and lips as well as in the nasal bridge and forehead. The most prominent perceptual differences between both shape transformations include the width of the nose, the size and spacing of the orbits and eyes and the shape of the lower face. Focusing on the eyes and orbits, there are changes in orbital curvature (Fig S41) and they exhibit a normal displacement (Figure S43). Related FSCPs noted in Table S4 include, shallow orbits, downslanted palpebral fissures, eyes widely spaced, and one out of two FSCPs for large eyes. The cheeks are mainly changed in terms of area (Fig S39) and the FSCP for sunken cheeks is significant. The nose is affected in many ways and it is one of the most striking features of this RIP-G (Figs S39, S41 and S43), clearly progressing from a wide to a thin nose in the shape transformations. All nasal related FSCPs in Table S4 are noted for this SNP including nasal bridge depressed and nose width. Area changes (Fig S39), curvature changes (Fig S41) and normal displacements (Fig S43) are noted for the lips or parts of the lips and the noted related FSCPs in Table S4 including lip thickness and mouth width. Although perceptually different in the shape transformations, the chin itself, The FSCP for micrognathia is not significant nor is the chin region significant for the area FSCP.
- The mouse homologue for the human aspartate beta-hydroxylase (***ASPH****)* gene (OMIM#600582) when knocked out leads to a shortening of the length of the snout, mild palatal changes, and syndactyly of both front and rear paws [11].
- Statistically significant normal-range effects of the SNP rs4738909 in *ASPH* are seen in Figure S40 with the strongest effects located in the midface/philtrum area and the mandible as well as the eyes and orbits. We observe a strong change of the facial profile from concave to convex in the lateral shape transformations (Fig S39). Accordingly, the relative inward/outward movement of the midface is opposite in sign to the inward/outward movement of the upper and lower face (Fig S43) and the results on mid-face retrusion in Table S4, support differences in this region.
- Mutations in the human gene DNA methyltransferase 3B (***DNMT3B***;OMIM#602900) located on chromosome 20q11.21 are associated with immunodeficiency-centromeric instability-facial anomalies syndrome 1 (ICF1; OMIM#242860). The facial phenotype of this autosomal recessive disease includes hypertelorism, a flat nasal bridge, epicanthal folds, mild micrognathia, a high forehead, and a small upturned nose.
- Statistically significant normal-range effects of the SNPs rs2424905 and rs2424928 in *DNMT3B* are depicted in Figure S40. The effects of both SNPs are highly similar and mainly focus on the eyes, orbits, nose, forehead, and mouth. In the lateral views of the shape transformations (Fig S40) a small and upturned nose with a flat nasal bridge are perceptually noted. The nose and in particular the nasal bridge exhibits area (Fig S40) and curvature changes (Fig S42) as well as normal displacement changes (Fig S44). Significant nose-related FSCPs include, nasal ridge narrow and retruded, nasal bridge depressed, nose snubbed, nares anteverted, and nasal tip size. Many of them are concordant with the small and upturned nose with flat nasal bridge perceived in the shape transformations. The lower parts of the eyes and orbits are perceived to be retrusive and related FSCPs noted in Table S4 include, shallow orbits, superiorly oriented orbits, palpebral fissures downslanted, proptosis, and eyes widely spaced (rs2424928 only). Some cranial aspects are affected such as microcephaly (head circumference), frontal bossing, prominent metopic ridge, and forehead sloping. However, the actual height of the forehead is not significantly changed. Finally, for both SNPs, one out of three measures for micrognathia is noted in Table S4.
- Mutation in the human gene ***RELN*** (OMIM#600514) located on human chromosome 7q22.1 can lead to the autosomal dominant condition Lissencephaly 2 (Norman-Roberts type; OMIM#257320), which is characterized by severe microcephaly, bitemporal hollowing, a sloping forehead, hypertelorism, and a broad and prominent nasal bridge.
- The normal-range effects of the SNP in rs471360 in *RELN* are shown in Fig S40 with the strongest effects located in the eyes and nasal bridge, as well as the lip and philtrum. Perceptually, the strongest differences between the shape transformations include an overall facial shape change and a variation in nasal shape, most strikingly in nasal bridge. Area changes and curvature changes are given in Fig S40 and S42, respectively. Several aspects of facial characteristic area and curvature changes are noted, which coincides with overall facial shape changes as noted in the shape transformations. Also notable, are the normal displacements (Fig S44) in the forehead. An opposite change in movement in the lateral parts of the forehead compared to the metopic ridge is seen. The interesting FSCPs related to the forehead in Table S4 include, frontal bossing, forehead sloping, forehead width, and microcephaly. The ones related to the nose include depressed and wide nasal bridge (both linked with broad and prominent nasal bridge), retruded nasal ridge, snubbed nose and anteverted naris and nasal tip size. The FSCPs for eyes widely spaced (associated with hypertelorism) are not noted. In contrast, multiple FSCPs at the eyes and orbital regions are noted.
- The ***UFD1L*** gene located on 22q11.22 and it is found within the region commonly deleted in 22q11.2 deletion disorder. Deletions in this region can be associated with DiGeorge syndrome (DGS) (OMIM#188400) and Velocardiofcial syndrome (VCFS) (OMIM#192430). Some craniofacial features reported in DGS patients include telecanthus (shortening of the distance between the eyes), short palpebral fissures, upward or downwards slanting eyes,a short philturm, a small mouth, a bulbous nose, a square tip of the nose, cleft palate, a broad nasal base, retrognathia, and narrow alae nasi. Patients with VCFS have craniofacial phenotypic features that can include a cleft palate, a tubular nose, short or almond-shaped palpebral fissures, retrognathia, small alae nasi, a bulbous nasal tip, a small mouth, and a broad nasal base.
- The statistically significant normal-range effects of the SNP rs2073730 in *UFD1L*, as depicted in Fig S40, are located in the nose and philtrum, as well as in the eyes, orbits, mouth and cheeks. Changes in positioning of the eyes and chin, and changes in size of the nose and mouth can be noted perceptually when looking at the shape transformations. In the nose a notable change in area (Fig S40) and normal displacement (Fig S44) is observed. Additionally, half of the FSCPs for cleft lip and palate are noted (Table S4). Normal displacements, opposite in direction tothose of the nose, are present in the cheeks and in the eyes. These normal movements all together have the effect of malar flattening, which can also be seen in Table S4: two out of three FSCPs are significant for malar flattening. In the lower face, significant area changes are observed. Significant FSCPs here include mouth width and thickness of the lips. However, even though clear area changes are noted in the chin region and retrognathia is visually observed in the shape transformations (Figure S40), only one of the three FSCPs is noted for retrognathia.
- Mutations in the human ***ROR2*** gene (OMIM#601227) located on human chromosome 9q22.31 can lead to two disorders with a t craniofacial phenotype: Robinow syndrome (OMIM#268310) is an autosomal recessive disorder characterized by macrocephaly, a broad and prominent forehead, low-set ears, ocular hypertelorism, prominent eyes, midface hypoplasia, a short upturned nose with depressed nasal bridge, flared nostrils, a large and triangular mouth with exposed incisors and upper gums, gum hypertrophy, misaligned teeth, ankyloglossia and micrognathia; Brachydactyly type B1 (OMIM#113000) is an autosomal recessive disorder facially characterized by a prominent nose, a high nasal bridge, and hypoplastic alae nasi.
- The normal-range results of the SNP rs7029814 in *ROR2* depicted in Fig 40 indicate effects in the midface, eyes and lower face. Perceptually, the strongest differences in the shape transformations include changes in midfacial prominence, nasal width, eye spacing, and eye prominence. Area changes (Figure S40) are most evident in the midface, with opposite changes in between the lips and beneath the lower lip as well as the eyes and orbits and lower mandible border. Curvature changes (Fig S42) are most prominent in the orbits and eyes as well as the cheekbones. Normal displacements (Fig S44) are observed in the midface and lower chin border, with opposite movements in the eyes, upper and lateral orbits, cheeks and the area between the lips. Regarding the forehead, the following FSCPs in Table S4 are noted: one out of two measures for microcephaly, a possible, but non-significant, tendency (p=0.064) for frontal bossing, a prominent metopic ridge, and one of two measures for forehead sloping. For the orbits and eyes, the noted FSCPs in Table S4 include, all but one FSCPs for shallow orbits, eyes widely spaced, proptosis, and large eyes. Midface retrusion, malar flattening, and prominent maxilla are noted for the midface FSCPs as is one of two measures for nasal bridge width, and a wide nose with nares anteverted. Finally, a long philtrum, wide mouth and micrognathia are noted.
- ***FGFR2*** is located on the chromosomal locus 10q26.13. Mutations in *FGFR2* are implicated in a number of craniofacial disorders with overlapping features: Antley-Bixler syndrome without genital anomalies or disordered steroidogenesis (OMIM#207410) is characterized by craniosynostosis, midfacial hypoplasia, proptosis, frontal bossing, and depressed nasal bridge. Other features include a pear shaped nose. Crouzon syndrome (OMIM#23500) is characterized by hypertelorism,, a beaked nose, a short upper lip and mandibular prognathism. Apert syndrome (OMIM#101200) is characterized by craniosynostosis midfacial hypoplasia. Other features include retrusion and elevation of the supraorbital ridge and strabismus. Characteristic features of Beare-Stevenson cutis gyrata syndrome (OMIM#123790) include craniosynostosis, hypertelorism, midfacial hypploasia, a low nasal bridge, anteverted nares, downward slanting palpebral fissures, and a small mouth. Bent bone dysplasia syndrome (OMIM#614592) is characterized by craniosynostosis, midfacial hypoplasia and hypertelorism. Features of Jackson-Weiss syndrome (OMIM#123150) include craniosynostosis and midface hypopasia. Some patients of Lacrimoauriculodentodigital syndrome (OMIM#149730) show features such as a broad anterior fontanelle, a high forehead and micrognathia. Craniofacial features of Pfeiffer syndrome (OMIM#101600) include craniosynostosis, midface deficiency, a prominent anterior fontanelle, scaphocephalymaxillary retrusion., and mental retardation (OMIM#609579) is characterized by macrocephaly, hypertelorism, and maxillary retrusion.
- The normal-range results of the SNP rs2278202 in *FGFR2* are shown in Fig 40. Affected areas include the nasal tip, lips, cheek, paranasal tissues, eyes and forehead. Perceptually, the strongest effects are located in the nose (anteverted nares), the eyes (downslanting and protruding eyes) and the mouth. Moreover, the length of the face appears to change. In Table S4, different FSCPs can be noted supporting these observations. Area changes (Fig S40) occur in the lower lip, the nose and the forehead, with opposite changes in the philtrum and parts of the midface. Curvature changes (Fig S42) are predominantly observed at the cheeks, the lips, philtrum, and parts of the nose, and around the orbits. Normal displacements (Fig S44) are located at the chin, cheeks, supraorbital ridge, the superior/medial part of the forehead, the nares and the lips. These movements induce effects such as micrognathia, flat midface, underdeveloped supraorbital ridge, frontal bossing and anteverted nares. Numerical results for these effects, in terms of FSCPs, can also be found in Table S4. Also, due to the activity in the nose and philtrum, 5 out of the 6 FSCPs for cleft lip and palate are noted.
- Mutations in the gene *FBN1*, located on 15q21.1, can lead to Marfan Syndrome (OMIM#154700). Some craniofacial features associated with these disorders include: a long and narrow face, malar hypoplasia, micrognathia, retrognathia, enophthalmos, shallow orbits, hypertelorism, downslanting palpebral fissures, a high-arched palate, micrognathia, an upturned nose, and posteriorly rotated ears.
- The normal-range effects of the SNP rs6493315 in *FBN1* shown in Fig S40, are in the mouth corners, the lateral parts of the mandible, the philtrum, and the medial parts of the midface, the eyes and the forehead. Perceptually, the strongest differences are observed in the chin, the eyes and the midface (protrusion of the cheekbones). On the shape transformations, when viewed from the side in Fig S40, an upturning of the nose is also visible. Significant area changes (Fig S40) and normal displacement (Fig S44) are observed in the chin and the forehead and noted FSCPs include micrognathia and sloping forehead (Table S4). Normal displacement is also present in the cheekbones, with opposite movement in the cheeks, which could be consistent with malar flattening. Moreover, in the midface a change in area is also observed. The FSCP for eyes widely spaced is noted.
- Even though mutations in ***GDF5***, located on 20q11.22 are associated with limb dysmorphology and Campiella and Martinelli reported a snubbed nose as a feature of 2 sibs with acromesomelic dwarfism (OMIM#201250).
- The normal-range results of the SNP rs143384 in *GDF5* depicted in Fig S40, indicate strong effects in the nose, as well as in the in the chin, eyes and cheekbones. When observing the shape transformations, main effects appear to be an upturning of the nose, a depression of the cheekbones and of the chin. These effects are also noted in terms of the FSCPs: nose snubbed, malar flattening and micrognathia. In terms of area changes (Fig S40), the cheekbones, chin and metopic ridge are affected, with opposite changes in the nasal tip, the philtrum and the upper lip. The strongest curvature changes (Fig S42) are located at the columella, the eyes and the lower lip, as well as at the orbital ridges and the chin. Normal displacements (Fig S44) are present mostly in the nose, the philtrum and the eyes, and in the cheekbones and the chin in the reverse direction. Significant FSCPs are noted in the nose and the eyes (Table S4). Presumably du to the activity in the nose and philtrum, all of the FSCPs for cleft lip and palate are significant.
- Mutations in the human gene ***COL11A1*** located on 1p21.1 can lead to three human diseases showing craniofacial involvement: Fibrochondrogenesis (OMIM#228520), Marshal Syndrome (OMIM#154780), and Stickler Syndrome type II (OMIM#604841). The first is autosomal recessive and the latter two conditions are autosomal dominant. Features of fibrochondrogenesis are a flat midface with a small nose and anteverted nares. There has been much discussion over the distinctiveness of Marshal and Stickler syndrome both of which can show a flat midface, a flat malar region, frontal bossing, micrognathia, a depressed nasal bridge, anteverted nares, cleft palate, a short depressed nose, long philtrum, hypertelorism, epicanthal folds, and thick lips.
- The normal-range effects of the SNP in rs11164669 in *COL11A1* are shown in Fig S40 and mainly focus on the eyes, orbits, nose tip, lips, and philtrum, and the lateral parts of the mandible. Perceptually in the shape transformations, the eyes and orbits change the most, with on one end protruding eyes with downslanting palpebral fissures and on the other end prominent cheekbones. Area changes (Fig S40) are observed for the lower lip, cheeks, lateral/upper orbits and eyes. Curvature changes (Fig S42) and normal displacements (Fig S44) are strong in the eyes and orbits with other regions affected as well. One out of two FSCPs for flat midface is noted, while the other shows borderline significance (p=0.077). The flat midface however is perceptually evident in the shape transformations, however we suspect this perception is partly due to changes in the eyes/orbits and mandible. For the midface, one out of three FSCPs for malar flattening and one out of two FSCPS for maxilla prominent are noted. One out of three FSCPs for micrognathia is noted. For the eyes and orbits, the FSCPs for shallow orbits, downslanted palpebral fissures, widely spaced eyes (one out of three FSPCs), large eyes and proptosis are noted. For the nose, significant results in Table S4 include depression and width of the nasal bridge, nose width, and two out of four FSCPs for nasal ridge width. The nasal tip size and anteverted nares are not noted in Table S4. Frontal bossing is noted and the FSCP for long philtrum has a suggestive but non-significant p-value (*p*=0.052). Only two out of six FSPCs for cleft lip and palate are noted, as there is minimal activity in terms of area change (Fig S40) and normal displacement (Fig S44) and only some activity in terms of curvature change (Fig S44) in the philtrum and palate region. Finally, the FSCP for lip thickness, and in particular the lower lip, is noted.

**REFERENCES:**

1. Lai TL, Robbins H, Wei CZ (1978) Strong consistency of least squares estimates in multiple regression. Proceedings of the National Academy of Sciences of the United States of America 75:3034–3036.

2. Abdi H (2003) Partial Least Squares ( PLS ) Regression. Encyclopedia of Social Sciences Research Methods (Eds. Lewis-Beck M, Bryman, A, Futing T) pp 1-7.

3. Yeniay O, Goktas A (2002) A comparison of partial least squares regression with other prediction methods. Journal of Mathematics and Statistics 31:99–111.

4. Anderson MJ, Legendre P (1999) An empirical comparison of permutation methods for tests of partial regression coefficients in a linear model. Journal of Statistical Computation and Simulation 62:271–303.

5. Claes P, et al. (2012) Sexual dimorphism in multiple aspects of 3D facial symmetry and asymmetry defined by spatially dense geometric morphometrics. Journal of Anatomy 221:97–114.

6. Shrimpton S. et al. (2014) A spatially-dense regression study of facial form and tissue depth: Towards an interactive tool for craniofacial reconstruction. Forensic Science International 234:103-110.

7. Walters M, Claes P, Kakulas E, Clement GJ (2013) Robust and regional 3D facial asymmetry assessment in hemimandibular hyperplasia and hemimandibular elongation anomalies. International Journal of Oral and Maxillofacial Surgery 42:36–42.

8. M. De Smet (2012) Generic 3-D Models for the Parameterization of the Human Face. Ktholieke Universiteit Leuven.

9. Rosenfeld JA, et al. (2009) Small deletions of SATB2 cause some of the clinical features of the 2q33.1 microdeletion syndrome. PLoS One 4:e6568.

10. Allanson JE, Greenberg F, Smith C. (1999) The face of Smith-Magenis syndrome: a subjective and objective study., Journal of Medical Genetics 36:394–397.

11. Dinchuk JE, et al. (2002) Absence of post-translational aspartyl beta-hydroxylation of epidermal growth factor domains in mice leads to developmental defects and an increased incidence of intestinal neoplasia. The Journal of Biological Chemistry 277:12970–12977.
